# Developing MYC Degraders Bearing the Von Hippel–Lindau Ligand to Target the “Undruggable” MYC

**DOI:** 10.1021/acsptsci.4c00452

**Published:** 2024-11-15

**Authors:** Christos Siokatas, Alexandra Lampropoulou, Alexandra Smina, Katerina Soupsana, Martha Kontostathi, Athina-Vasiliki Karra, Theodoros Karampelas, Anastasia S. Politou, Savvas Christoforidis, Constantin Tamvakopoulos, Vasiliki Sarli

**Affiliations:** † Vasiliki Sarli - Department of Chemistry, Aristotle University of Thessaloniki, University Campus, Thessaloniki 54124, Greece; ‡ Constantin Tamvakopoulos - Center of Clinical Research, Experimental Surgery and Translational Research, Division of Pharmacology-Pharmacotechnology, Biomedical Research Foundation, Academy of Athens, Soranou Ephessiou Street 4, Athens 11527, Greece; § Laboratory of Biological Chemistry, Department of Medicine, School of Health Sciences, University of Ioannina, Ioannina 45110, Greece; ∥ Biomedical Research Institute, Foundation for Research and Technology, Ioannina 45110, Greece

**Keywords:** PROTACs, MYC, biphenyl pyrazole, targeted protein degradation, **MYCi361**, **MYCi975**

## Abstract

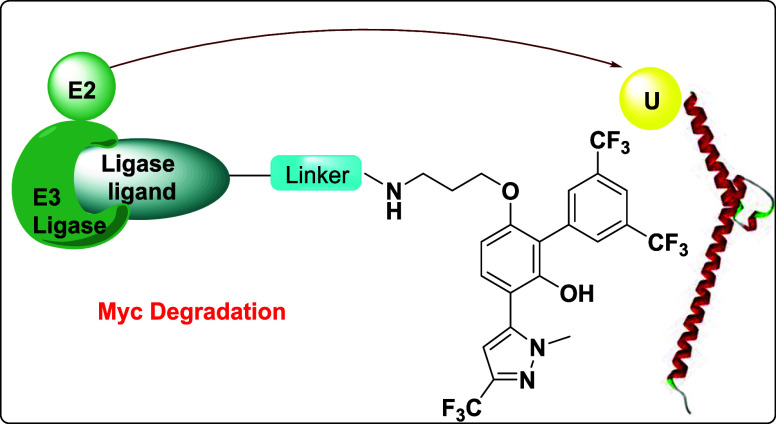

Although small-molecule inhibitors with moderate efficacy targeting MYC have been previously described, to this point, research efforts have failed to bring a suitable small-molecule MYC inhibitor to the clinic. Herein, the discovery of a series of novel MYC degraders bearing VHL to target the “undruggable” MYC is presented. The molecules are based on connecting a known MYC binder to a VHL ligand or pomalidomide to induce MYC degradation in various cancer cells known to express MYC. Representative compounds from our work induced MYC degradation in a time- and dose-dependent manner. Selected compounds, **CSI86** and **CSI107**, displayed antiproliferative activity (IC_50_ values of 13–18 μM) against breast and prostate cancer cells. The lead molecules were further evaluated in terms of cell uptake, potential to degrade MYC, and pharmacokinetics in mice. Encouraging results presented herein suggest that the presented analogs may serve as prototype structures of future therapeutic agents for the treatment of MYC-dependent tumors. MYC protein degraders can well complement the more established inhibition approaches that have been presented in the past (e.g., disruption of the MYC–MAX complex formation by small-molecule inhibitors).

The MYC family of proteins, encoded by the *MYC* oncogene, consists of four transcription factors: c-MYC, L-MYC, s-MYC, and N-MYC. MYC proteins are involved in a variety of biological processes, including cell growth and differentiation, cellular metabolism and biosynthesis, apoptosis, DNA repair, and protein translation.^1^ Among the family members, c-MYC is ubiquitous in proliferating cells and is a master regulator of cell growth and apoptosis.^2^ The c-MYC protein forms dimers with Max transcription factors to bind to the E-box sequence (CACGTG) and regulate the transcription of target genes.^3^ MYC is overexpressed and/or activated in more than half of human cancers.^4^ MYC expression is normally activated by growth factors and is strongly regulated, with short half-lives for MYC mRNA and protein levels (30 and 20 min, respectively).^5^

MYC is deregulated in more than half of human cancers and profoundly affects cancer formation, maintenance, and progression. Its overexpression is commonly associated with poor prognosis and unfavorable patient survival.^6^ Over the years, it has become increasingly clear that the inhibition of MYC function represents an attractive strategy for anticancer drug development. Several compounds that directly or indirectly inhibit MYC have exhibited anticancer activity in preclinical tumor models, but to this point, research efforts have failed to bring a suitable MYC inhibitor to the clinic.^7−9^ MYC is considered a difficult target (often the term “undruggable” is used for this protein) since it is a disordered protein.

In recent years, technology leading to the proteolysis targeting chimeras (PROTACs) has emerged as an exciting new and possibly alternative approach to traditional drug discovery. PROTACs are considered fitting solutions to tackle “undruggable’’ proteins and to overcome drug resistance caused by target mutations.^10^ PROTACs act differently than the classic inhibitors and are catalytic in their mode of action.^11^ They have unique features to target proteins since they allow efficient inactivation of the target and inhibition of its scaffolding function.^12^ PROTACs induce ubiquitination and degradation of a protein of interest (POI) by the proteasome system UPS. They are bifunctional hybrid molecules consisting of three moieties: a ligand binding to the POI, a ligand that can recruit an E3 ubiquitin ligase, and a linker. Following the PROTAC methodology, Schneider and coworkers have developed a MYC PROTAC (MDEG-541) based on the inhibitor 10058-F4 derivative with thalidomide (Figure 1).^13^ Notably, MDEG-541 inhibited the viability of HCT116 and PSN1 cells with GI_50_ values of 14.3 μM and 10.7 μM, respectively. As a proof-of-concept study, the work is noteworthy as it demonstrates how a PROTAC (MDEG-541) can bind MYC and cause cereblon (CRBN)-dependent proteasomal degradation. However, the study’s scope is somewhat limited as it does not address cell penetrability, pharmacokinetic, or toxicity properties in animal models. Moreover, Li et al. synthesized a threose nucleic acid (TNA) aptamer capable of binding to the c-Myc/Max heterodimer and appended to the E-box DNA sequence to create a high affinity, biologically stable bivalent binder. The TNA-E box-pomalidomide (TEP) conjugate specifically degrades endogenous c-Myc/Max in triple-negative breast cancer cells.^14^ However, this important approach has some limitations, commonly faced with nanomedicines, such as the dosing complexities (cell transfection of TEP seems to be essential for c-Myc degradation studies and efficacy studies in mouse models using liposome nanoparticles modified with cyclic RGDyK peptides).^14^

**Figure 1:**
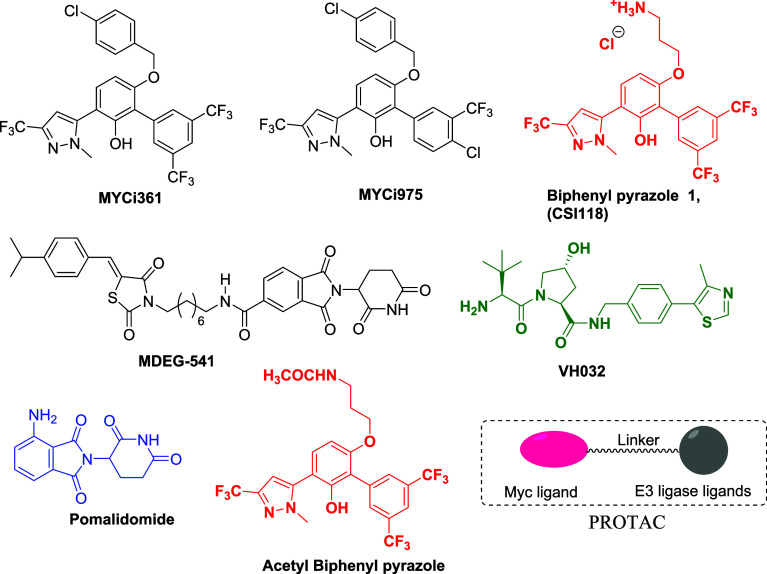
Structures of pyrazole-based MYC inhibitors and PROTAC MDEG-541.

Of significance to our work are studies by Han et al. that described biphenyl pyrazoles **MYCi361** and **MYCi975** as high-affinity MYC binders that disrupt the MYC/MAX complex formation.^15^ In addition, it was shown that the inhibitor **MYCi361** reduces MYC protein stability by promoting MYC T58 phosphorylation and increasing proteasome-mediated MYC degradation. Previous studies have shown the importance of T58 phosphorylation in the regulation of MYC function.^16^ T58 phosphorylation is recognized by E3 ubiquitin ligases such as SCFFbw7 and induces MYC degradation by the 26S proteasome.^17^**MYCi361** and **MYCi975** significantly inhibited the proliferation of tumor cells in vitro and suppressed tumor growth in vivo in a prostate cancer model. More recent studies showed that **MYCi975** decreased proliferation and induced apoptosis in breast cancer cell lines, being more efficient in triple-negative breast cancer.^18^ Furthermore, Zhao et al. recently demonstrated that a **MYCi975** analog induced dose-dependent MYC degradation in cancer cells, accompanied by enhanced antiproliferative effects in cells and therapeutic efficacy over **MYCi975** in a mouse allograft model of prostate cancer. Such studies offer critical insights into the field of targeting MYC for cancer.^19^ Following up on experiences gained from our work with small-molecule MYC inhibitors^20^ and based on accomplishments with hybrid structure bioactive molecule development,^21^ we have expanded our objectives to discover MYC-specific degraders. Herein, we present our approach to develop a new MYC degrader by connecting the known analog of **MYCi361** biphenyl pyrazole **CSI118** with either the CRBN ligand pomalidomide or the Von Hippel–Lindau (VHL) ligand VH032.^22,23^ MYC PROTACs that can efficiently induce degradation of MYC proteins in cell lines were identified and extensively evaluated by in vitro testing. In addition, following stability experiments and further work that showed that some of the described leads directly bind MYC, and effectively enter the cells, we proceeded to in vivo pharmacokinetic studies in mice, which provided further evidence that efficacy in cancer models is possible.

## Results

### Design of MYC Degraders

**Figure 2:**
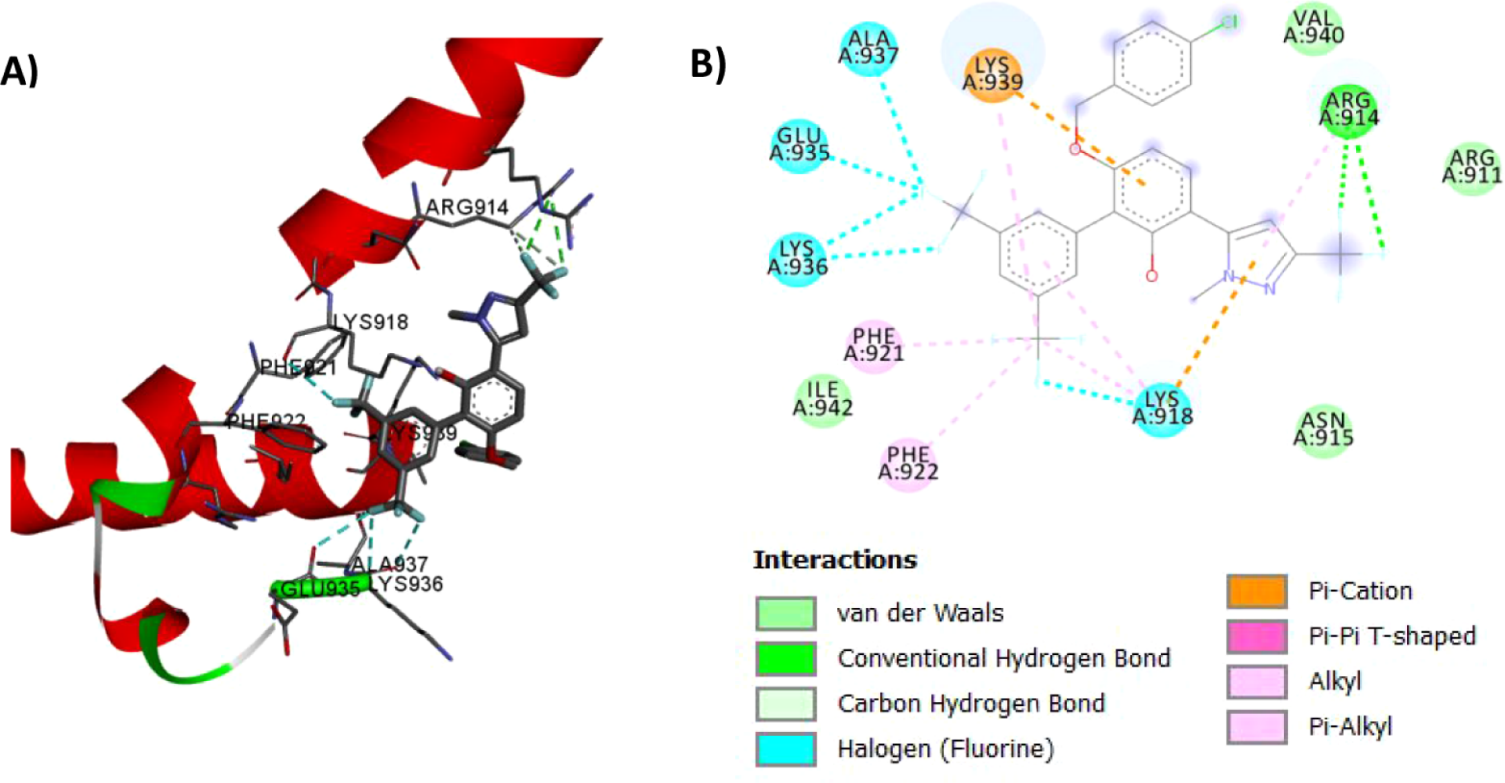
(A) 3D model of the interactions between compound **MYCi361** and MYC (PDB: 1NKP); (B) 2D molecular docking model of compound **MYCi361** and MYC. Hydrogen bonds are indicated by green lines to key amino acids.

To understand the binding mode of biphenyl pyrazoles to the MYC protein, in silico docking was conducted. The biphenyl pyrazole derivatives were energetically minimized and subjected to docking with the MYC protein (PDB:1NKP) using AutoDock Vina.^32^ Starting with **MYCi361**, the best binding pose demonstrated a binding affinity of −6.1 kcal mol^–1^ to MYC (Figure 2). This binding site was not located at the MYC–MAX interface. The compound was stabilized by various noncovalent interactions, including hydrogen and halogen bonds with residues of the MYC protein. The docking models reveal that the CF_3_ substituents of the phenyl group form halogen bonds with residues Ala937, Glu935, Lys936, and Lys918, while the CF_3_ substituent of pyrazole participates in hydrogen bonding with Arg914. A cation−π interaction between Lys939 and phenol was also observed. The acetyl derivative of biphenyl pyrazole **1** (**CSI118**) (Figure 1) was found to bind MYC with a binding affinity ranging from −6.8 kcal/mol in a similar way. The CF_3_ substituents of the phenyl group form halogen bonds with residues Ala937 and Glu935. In addition, two hydrogen bonds were formed between phenol and Glu935 as well as CF_3_ and Lys939. Based on our observations, the key interacting residues of biphenyl pyrazoles with MYC could be recognized. The *p*-chloro-benzyl and the 3-aminopropyl groups are not important for binding and are oriented toward the solvent. Therefore, these groups are suitable linker attachment points.

### Synthesis of MYC Degraders/PROTACs

**Scheme 1:**
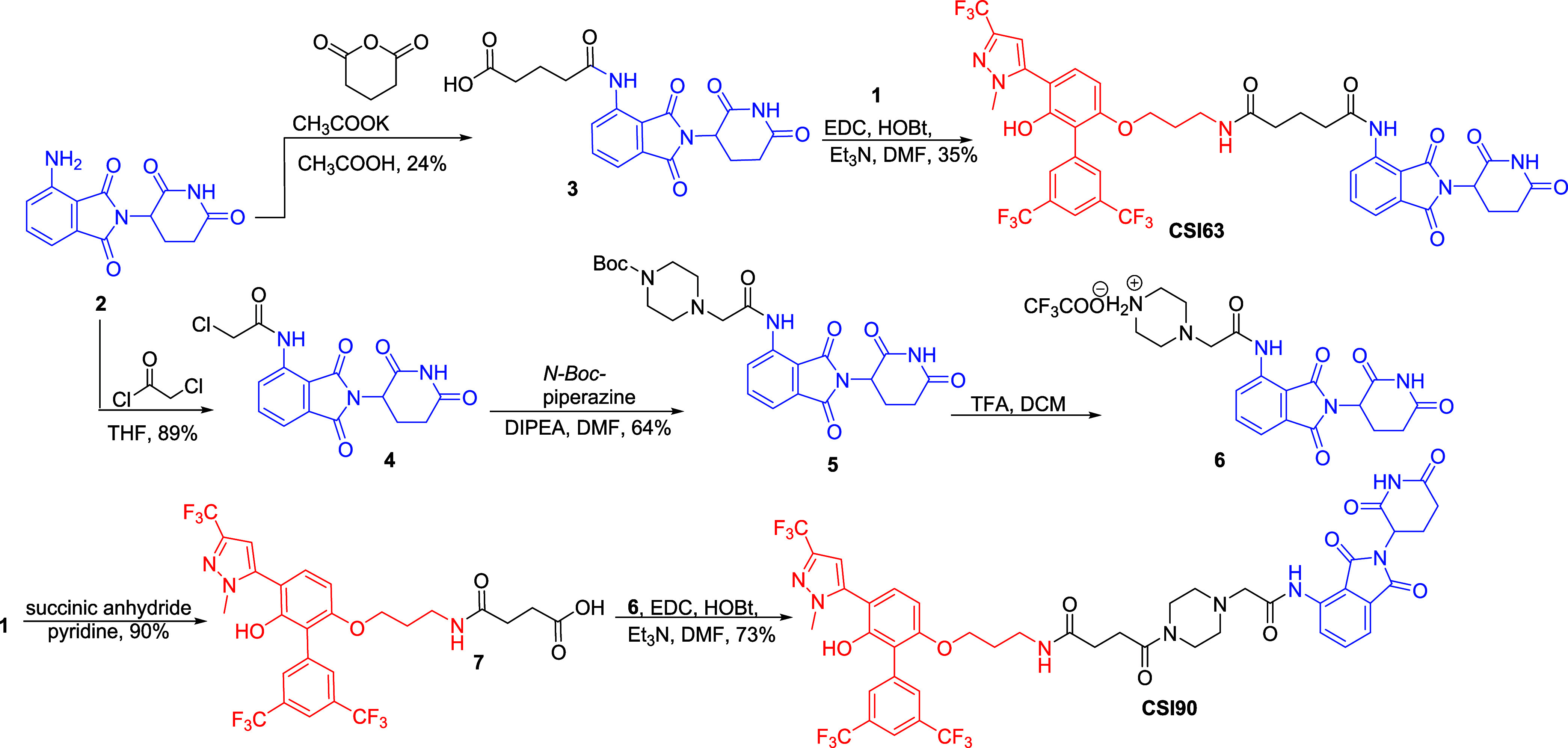
Synthesis of Pomalidomide-Based PROTACs

**Scheme 2:**
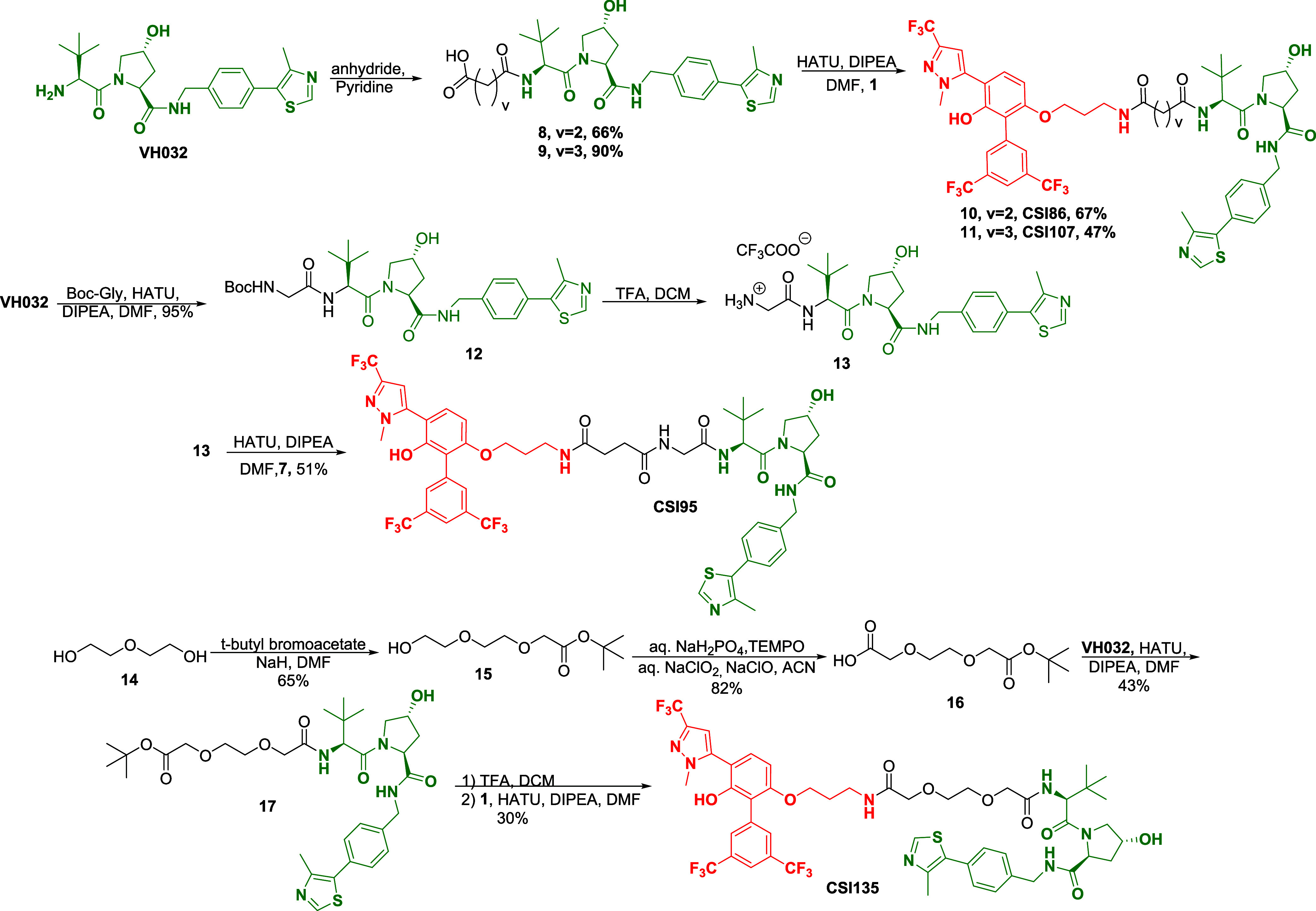
Synthesis of VHL-Based PROTACs

Our research efforts started with the synthesis of a small set of MYC degraders that vary in the linker length, bearing pomalidomide as the CRBN recruiter or the VHL ligand. The preparation of the designed compounds is depicted in Schemes 1 and 2. Biphenyl pyrazole **1** (**CSI118**), VHL ligand, and pomalidomide were synthesized by following previously reported routes.^15,25^

Two pomalidomide compounds were synthesized bearing a −CO(CH_2_)_3_CO– linker and a longer piperazine linker (Scheme 1). Pomalidomide reacted with glutaric anhydride in acetic acid and potassium acetate to give acid **3**, which was coupled with **1** using EDC as a coupling reagent and HOBt. Derivative **5**, bearing the piperazine linker, was prepared after the acylation of pomalidomide with 2-chloroacetyl chloride and substitution reaction of chlorine atom by piperazine. The reaction of biphenyl pyrazole **1** with succinic anhydride in pyridine produced derivative **7** in 90% yield. Next, deprotection of the Boc group of **5** using TFA in DCM yielded the free piperidine, which was coupled with **7** under standard coupling conditions to conjugate **CSI90** in 73% yield.

In addition, four compounds, bearing the VH032 ligand, were prepared by employing the −CO(CH_2_)_2_CO–, −CO(CH_2_)_3_CO–, −CO(CH_2_)_2_CONHCH_2_CO–, and −CONHCH_2_O(CH_2_)_2_OCH_2_CO– linkers (Scheme 2). Initially, the amino group of VH032 reacted with succinic or glutaric anhydride for the introduction of the linkers, and then, MYC inhibitor **1** was coupled with carboxylic acids **8** or **9**, producing conjugates **CSI86** and **CSI107**. To increase the length of **CSI86**, Boc**-**glycine was first coupled with VH032. The coupling product **12** was then deprotected using TFA in DCM to produce the trifluoroacetate salt **13**. The desired compound **CSI95** was obtained via standard amide coupling between acid **7** and trifluoroacetate salt **13** in 51% yield. Starting from diethylene glycol **14**, followed by a reaction with *tert*-butyl bromoacetate, oxidation and coupling with VH032, derivative **17** was synthesized. Conjugate **CSI135** was obtained after deprotection of **17** and coupling with compound **1**.

**Scheme 3:**
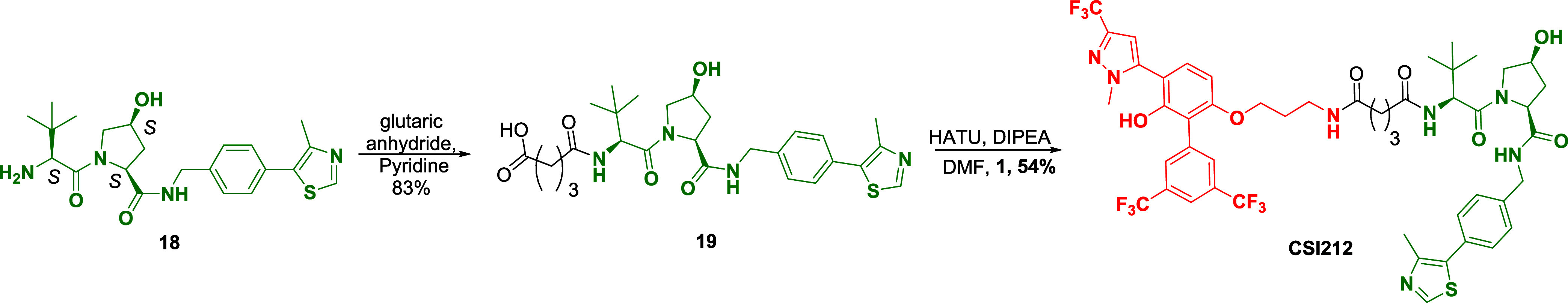
Synthesis of Negative VHL-Based PROTAC **CSI212**

In order to provide insights into the mechanism of action of the synthesized compounds, a negative control for the VHL ligand was developed. Since compound **CSI107** has been characterized as the best candidate of this work in terms of activity, cell uptake, and pharmacokinetic profile, the newly developed negative control was chosen to bear the glutaric linker. This compound (**CSI212**) has the 4-hydroxy group of 4-hydroxyproline in *S* conformation, whereas the VHL-based degraders have the 4-hydroxy group in *R* conformation. The “negative” VHL ligand ((*S*,*S*,*S*)-VH032-NH_2_, **18**) was developed via a synthetic route (Scheme 3) similar to the *trans*-VHL ligand (**VH032**),^25^ with the only significant difference being the use of the (2*S*,4*S*)-4-hydroxyproline instead of the (2*S*,4*R*)-4-hydroxyproline.

### Stability of PROTACs in Cell Medium

The stability of selected molecules, such as **CSI86**, was studied in cell medium over a time frame of 72 h at 37 °C. The conjugates were found to be stable after the 72-h period of incubation with DMEM, suggesting that any antiproliferative effects found (shown in Table 2) are due to the intact novel degrader and not to some degradant (e.g., active metabolite) forming during the experiment. Other representative conjugates (**CSI90** and **CSI95**) were evaluated in this assay with similar stability properties.

### MYC Degradation Following Treatment with PROTACs in PC3 Cell Lines

The ability of novel compounds to induce MYC degradation in the PC3 cell line was evaluated via a Western blot-based assay. The potential of the candidate PROTACs to reduce MYC levels was assessed following a 24-h incubation period of PC3 cells at a concentration of 10 μΜ. A PROTAC that effectively degrades bromodomain and extra-terminal (BET) family proteins, with indirect action on MYC levels, BETd-260, was also included in these experiments as a positive control. BETd-260 was tested at a lower concentration (1 μΜ) due to its high potency.^26^

**Figure 3:**
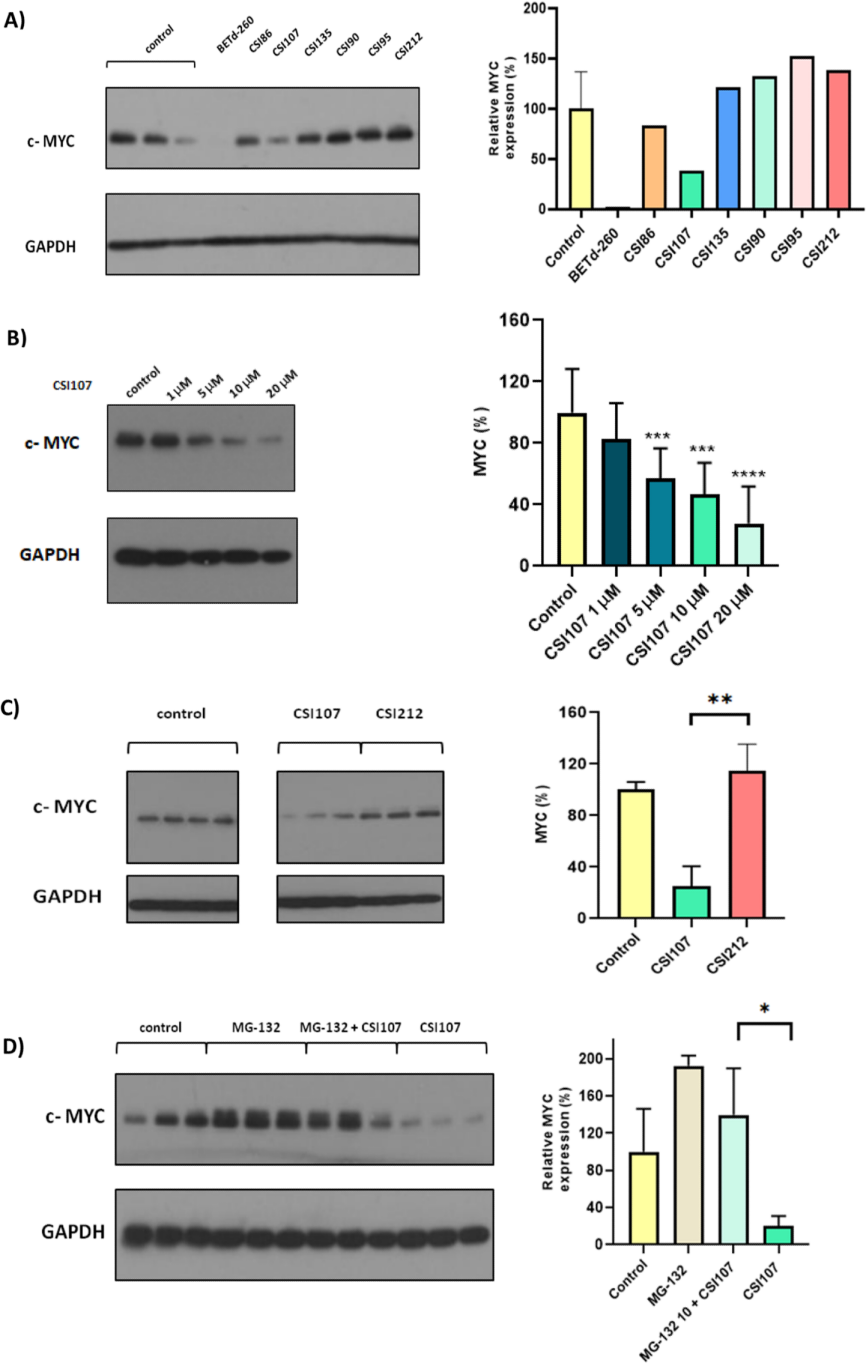
In vitro evaluation of c-MYC degradation. (A) PC3 cells were treated with the compounds of interest at 10 μΜ for 24 h, and a first screening via Western blot was carried out for the determination of MYC levels in this cellular model. BETd-260 was used at 1 μΜ as a positive control. (B) Dose-dependent degradation of c-MYC after treatment of PC3 cells with CSI107 at different concentrations for 24 h. Error bars represent mean ± SD of at least three independent representative biological experiments (**p < 0.01, ***p < 0.001, and ****p < 0.0001 versus control/cell media). (C) The lead compound in MYC degradation was evaluated at 10 μM in comparison to the negative control **CSI212** at 10 μM to demonstrate the PROTAC-mediated degradation of the POI (**p < 0.01). (D) Proteasome-dependent degradation of c-MYC is shown after pretreatment of PC3 cells with proteasome inhibitor **MG-132**, followed by incubation with the lead compound **CSI107** (*p < 0.05). Error bars represent mean ± SD, one independent representative biological experiment for (C,D) performed in triplicate versus control (the controls represent PC3 cells incubated in cell medium).

A first screening of all compounds included in the study demonstrated the degrading potential of **CSI107** and **CSI86** against MYC in PC3 cells (Figure 3A). No particular effect was observed in the degradation of MYC for **CSI135**, **CSI90**, and **CSI95** under the same experimental conditions. Additionally, we demonstrated that MYC degradation due to **CSI107** treatment is affected in a dose-dependent manner (Figure 3B) by testing the lead compound in various concentrations under the described experimental conditions (1–20 μΜ). Additional experiments were performed in order to monitor the levels of the c-MYC protein following incubation with **CSI107** for a shorter treatment period (3 h). The results showed that **CSI107** at 10 μΜ effectively mediated c-MYC degradation after 3 h of incubation in PC3 cells. This suggests that **CSI107** leads to c-MYC degradation (a protein with a known short half-life) (Figure S29). However, to ensure that the observed c-MYC degradation was indeed E3 ligase mediated, a negative control **CSI212**, a direct analog of **CSI107** with a modification in the E3 binding domain, was synthesized. The VHL-bearing MYC degrader **CSI107** was evaluated in comparison to **CSI212**. The results, shown in Figure 3C, demonstrate that **CSI212** was inactive when tested in the applied assay, whereas **CSI107** was significantly active (**p < 0.01, by unpaired *t*-test). The degradation mechanism was fully elucidated in PC3 cells by evaluation of the proteasome-mediated activity of **CSI107**. The cells were treated with the proteasome inhibitor MG-132 at 10 μΜ for 2 h, followed by the addition of **CSI107** at 10 μΜ for 24 h. The results, shown in Figure 3D, demonstrate that MYC degradation can be rescued by proteasomal inhibition in cells pretreated with **MG-132** as no reduction in MYC levels was observed compared to the control group. At least two independent experiments were performed in our studies with *n* = 2 or 3 biological replicates per experiment. As has been shown, **MYCi361** reduces MYC protein stability by promoting MYC T58 phosphorylation, leading to proteasome-mediated MYC degradation that can be rescued by MG-132.^23^ Similarly, under our experimental conditions, **MYCi361** also degraded MYC in the 5–10 μΜ range.

### PROTAC Direct Binding to MYC by Microscale Thermophoresis

**Figure 4:**
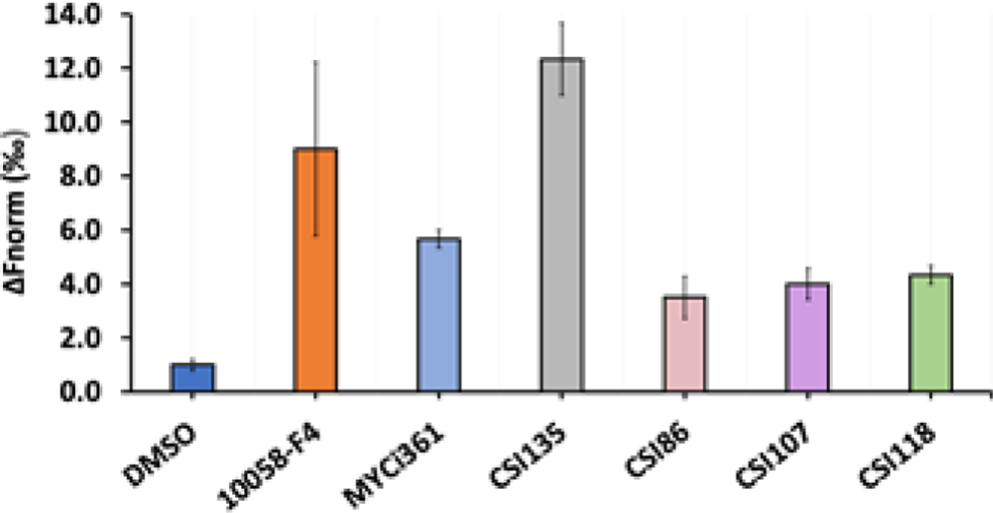
Assay of direct binding to MYC by microscale thermophoresis (MST): MST of fluorescently labeled MYC (25 nM) mixed with several MYC degraders (200 μM) or the solvent alone (DMSO). MST of MYC binders **10058-F4** and **MYCi361** used as positive controls. Data are shown as mean ± SEM.

To investigate the capacity of MYC degraders to directly bind to MYC, microscale thermophoresis (MST) experiments were performed using recombinant bHLH-MYC protein. MST is a very sensitive, recently developed method based on the shift of fluorescent molecules moving in a temperature gradient that can be perturbed by interactions with other molecules. In this binding assay, a His6-tagged bHLH-MYC protein was fluorescently labeled via its hexa-histidine tag and subjected to MST measurement in the absence and in the presence of MYC degraders. All compounds tested shifted MYC thermophoresis (Figure 4), as shown by normalized fluorescence values, a clear indication that they bound directly to MYC. MST experiments were also run under identical conditions using two reference Myc inhibitors as positive controls: the biphenyl pyrazole **MYCi361** and a thiazolidinone derivative, **10058-F4**. Both are well-characterized MYC binders: The first one has been used as a POI ligand for the PROTACs designed and synthesized in this work, and the latter is widely known to inhibit MYC by specifically disrupting the MYC–Max complex formation. As shown in Figure 4, all the compounds tested were efficient in targeting MYC by directly binding to the protein.

### PROTAC Inhibition of the Formation of the MYC:MAX Complex

**Figure 5:**
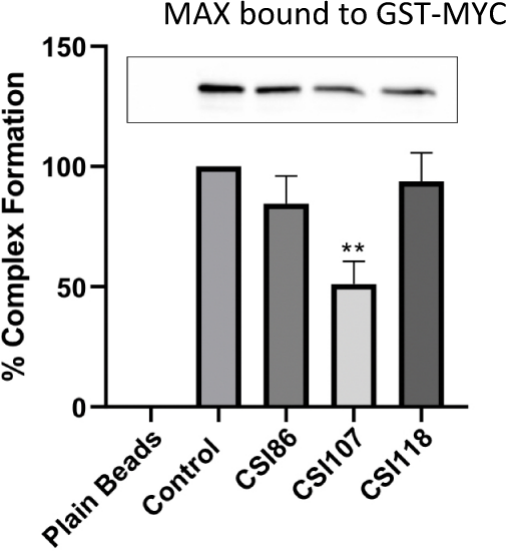
Inhibition of the formation of the MYC:MAX complex by the pyrazole compounds. The indicated compounds (150 μM), or the solvent alone (DMSO, used as a control), were incubated with GST-MYC containing glutathione beads for 10 min at 40 °C, followed by incubation with His-MAX. Plain beads do not contain GST-MYC but were incubated with His-MAX to assess the noise of the assay. The complex of GST-MYC:His-MAX was eluted by incubation of the beads with free glutathione. The amount of the eluted complex was analyzed by western blotting (presented in the inset), using anti-MAX antibodies, while the protein bands were quantified with the ImageJ software. The intensity of the bands is presented in the bar graph. **CSI86** and **CSI118** have no effect on MYC/MAX dimerization, while **CSI107** inhibits dimerization with statistical significance **p < 0.01. Statistics were based on the unpaired two-tailed student’s *t-*test. Data are shown as mean ± SEM.

To assess whether the new degraders affect direct interaction of MYC with MAX, we employed a pull-down approach using recombinant proteins that correspond to the bHLHZ regions of MYC and MAX. GST-MYC, bound to glutathione sepharose beads, was incubated with the pyrazole compounds, followed by incubation with His-MAX. Finally, the complex of GST-MYC/HisMAX was eluted by the addition of excess glutathione, while the amount of the eluted complex was assessed by western blotting using anti-MAX antibodies (Figure 5). Interestingly, **CSI107** inhibited the binding of HisMAX to MYC, suggesting that this compound interacts with MYC at domain(s) involved in its binding to MAX. The other degraders and inhibitor **CSI118** did not seem to interfere significantly with the MYC–MAX complex formation.

### Cell Uptake of Putative MYC PROTACs

**Figure 6:**
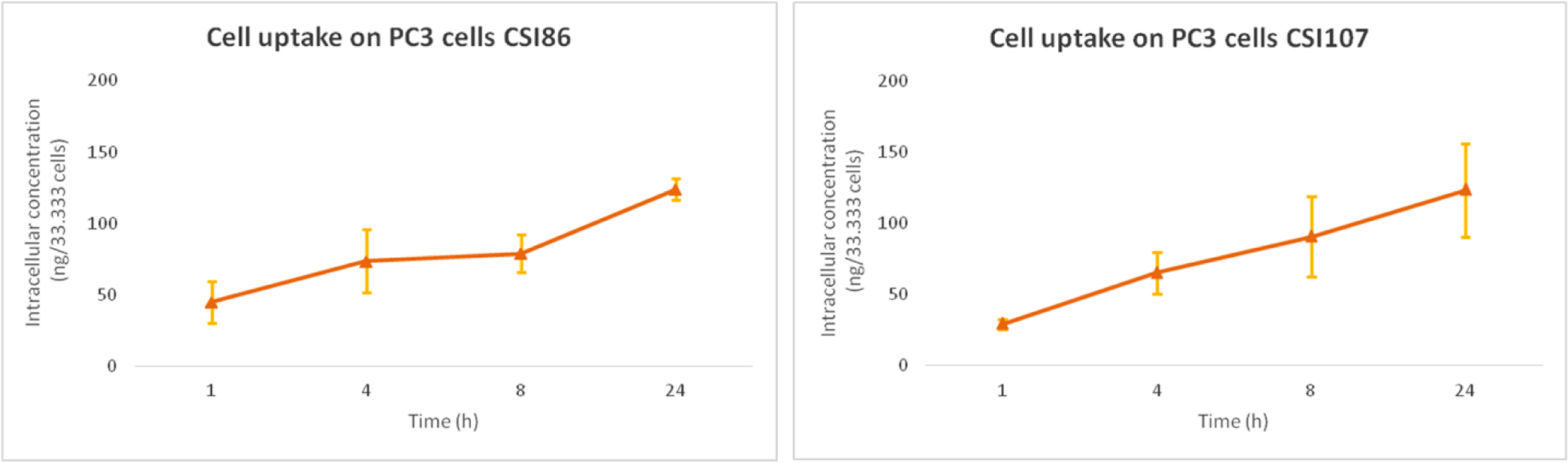
Cell uptake curve of **CSI86** and **CSI107** in PC3 cells after 24 h incubation with 10 μM.

**Figure 7:**
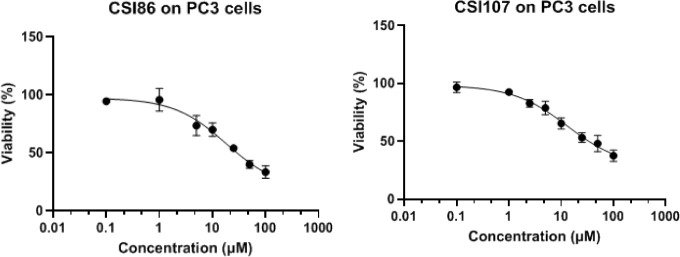
Cell viability curves assessed by the MTT assay on PC3 cells for **CSI86** and **CSI107**. Values shown represent the means of at least three experiments performed in triplicate ± SD.

Many of the synthesized derivatives and PROTACs described in the literature are lipophilic, with high MWs and a large number of rotatable bonds, and possess physicochemical properties that are beyond Lipinski’s rule of five.^27^ Therefore, in order to better comprehend the observed activities of our degraders, following incubation of each compound with cells, the intracellular concentrations of the synthesized derivatives were evaluated in different cancer cell lines, as described previously.^28,29^ SKBR3, PC3, or A549 cells were incubated with PROTACs (at a 10 μΜ concentration) for selected time points, respectively. Upon sample treatment, compounds were extracted and analyzed by LC-MS/MS. Table 1 presents the percentage of the molecule that enters the cells after 24 h incubation at the 10 μM concentration. All compounds tested generally displayed reasonable cell uptake (>10%) that encouraged further development, despite their limited drug-like properties. The highest uptake was observed in SKBR3 cells after treatment with **CSI86** (26%), corresponding to an intracellular concentration of 2.6 μM. Interestingly, **CSI86** uptake by A549 cells was lower (10%). **CSI107** also displayed good permeability, with approximately 19% uptake by SKBR3 (shown in Figure 6) and 13% by PC3 cells, respectively (Figure 7). Derivative **CSI95**, which bears the longer linker −CO(CH_2_)_2_CONHCH_2_CO–, and **CSI135**, which has the highest molecular weight, showed lower cell uptake values (less than 10%) when tested in PC3 cells, compared to **CSI86** (Figure 7). Finally, **CSI90**, which bears a long, complex linker bound to the CRBN moiety as an E3 ligand, did not have good permeability properties in PC3 cells (4% uptake). The small molecule **MYCi361**, used as a binding domain component of MYC, was also evaluated in the cell uptake assay (Table 1). As expected, due to its lower molecular weight, it displayed reasonably high cell uptake (19% and 28% by SKBR3 and PC3 cells, respectively).

**Table 1:** Percentage of the Synthesized Molecules That Enters the Cell Following 24 h Incubation with 10 μM of Each Compound^a^

Compound	Cell line	Uptake by cells
**CSI90**	SKBR3	18 ± 3%
	PC3	8 ± 6%
**CSI86**	SKBR3	26 ± 5%
	PC3	19 ± 4%
	A549	10 ± 2%
**CSI107**	SKBR3	19 ± 3%
	PC3	13 ± 3%
**CSI95**	SKBR3	16 ± 5%
	PC3	9 ± 3%
**CSI135**	PC3	7 ± 2%
**MYCi361**	SKBR3	19 ± 1%
	PC3	28 ± 5%

a The cell uptake results are derived from the mean ± SD of three independent experiments.

### Diphenyl Pyrazole-Based PROTACs Inhibit Cell Growth in Cancer Cell Lines

The antiproliferative activity of the synthesized compounds was tested in cell viability assays optimally set up for various cancer cell lines (results are depicted in Table 2, *n* = 3, average IC_50_ (μM) ± SD). Notable growth inhibition was observed after treatment of the cells with derivatives of the VHL ligand, while compounds bearing the pomalidomide ligand were found significantly less active (IC_50_ values of >50 μM). Derivative **CSI86** inhibited the cell growth of human breast (SKBR3) and prostate cancer cells (PC3) with IC_50_ values of 15 ± 4 μM and 18 ± 7 μM, respectively (Figure 7). The recorded activities were comparable with the activity of **MYCi361** with IC_50_ values of 8 ± 1 μM in PC3 cells. Compound **CSI86** was inactive in A549 lung cancer cells (inactive at concentrations as high as 100 μM). Derivative **CSI107** was equally potent with **CSI86**, with IC_50_ values of 13 ± 7 μΜ in the PC3 cell line and 14 ± 2 μΜ in the SKBR3 cell line. **CSI135** had IC_50_ values of 25 ± 2 μM in the PC3 cell line (Table 2).

**10058-F4** was also used as a positive control in the cell viability assay in PC3 cells (half maximal inhibitory concentrations *n* = 2, IC_50_ = 23 and 25 μM).

Importantly, compound **CSI212**, synthesized as a negative control having the 4-hydroxy group of 4-hydroxyproline in the *S* conformation (versus the VHL-based degraders with the 4-hydroxy group in *R* conformation), did not have any antiproliferative effect in prostate and breast cancer cell lines, further implicating the importance of the E3 ligase recruitment for potency in cell lines.

Therefore, two active degraders (**CSI186** and **CSI107**) bearing the VHL ligand bound to the MYC inhibitor **CSI118** via relatively short linkers −CO(CH_2_)_2_CO– and −CO(CH_2_)_3_CO–, and **CSI135** bearing the longer −COCH_2_O(CH_2_)_2_OCH_2_CO–, were identified for further evaluation. The observed antiproliferative effects can be explained in part due to the achieved intracellular concentration of the synthesized compounds upon incubation with cell lines. For example, **CSI86** showing high activities in SKBR3 and PC3 cell lines also has a high percentage of cell uptake, 26% and 19%, respectively. Equally interesting was analogue **CSI107**, with antiproliferative properties comparable to those found for **CSI86**. Although direct correlations between antiproliferative effects and cell uptake would not be possible before more data is obtained, the cell uptake assays are a valuable tool for making decisions on selecting the most suitable compounds for further examination.

### In Vivo Pharmacokinetic Studies

In order to assess the efficacy potential of MYC degraders in animal models, pharmacokinetic studies were carried out in mice (Figure 6A,B). Following intraperitoneal (i.p.) administration of selected compounds, **CSI86**, **CSI135**, and **CSI107** at 10 mg/kg, blood samples were collected at different time points until 24 h post-drug administration, and concentrations were determined via LC-MS/MS. Reasonably high concentrations of circulating PROTACs were detected as early as 2 h postdose (*C*_max_ = 713 ng/mL, 413 ng/mL, and 699 ng/mL for **CSI186**, **CSI107**, and **CSI135**, respectively).

**Figure 8:**
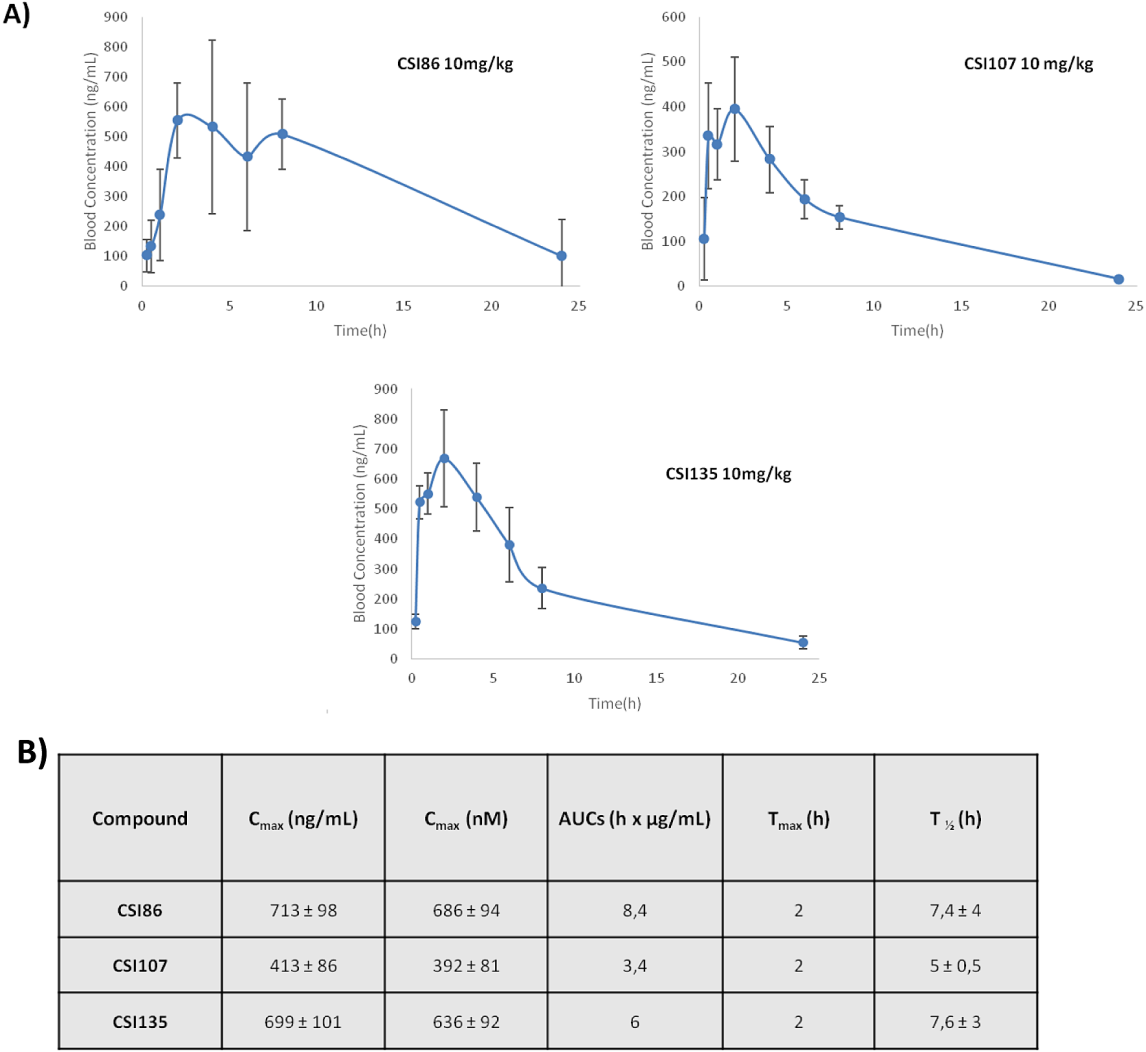
In vivo pharmacokinetic studies of **CSI86**, **CSI107**, and **CSI135**. **A)** Blood concentration–time curves after i.p. administration at 10 mg/kg in mice; **B)** pharmacokinetic parameters. The number of replicates was 4–5 mice per time point, with the error representing the standard deviation for the corresponding average values.

Pharmacokinetic studies indicated that 8 h after the administration of the compound, average concentrations were notable, ranging from approximately 150 ng/mL (∼140 nM) for **CSI107** up to 500 ng/mL (∼480 nM) for **CSI186**. This finding suggests a long half-life (ranging from 5 to 7.6 h) for the corresponding degraders, further encouraging future evaluations in mouse models. Although the calculated concentrations of degraders of interest were notably lower in comparison to the 5–10 μΜ IC_50_ values found in the antiproliferative studies, this is of no significant concern since the PK studies performed were at a single 10 mg/kg dose in C57BL/6J naïve mice. Typically, efficacy studies are performed based on multiple days of dosing at much higher doses (e.g., 50 or 100 mg/kg)^23^ in immunodeficient animal models. Moreover, the concentration at the target site, e.g., the tumor (not necessarily the values of circulating concentrations), is what is of utmost importance, something that represents the scope of our future studies (Figure 8).

Additionally, an oral gavage (PO) dose study of **CSI86** in mice (at 10 mg/kg) resulted in concentrations that were not appreciable (*C*_max_ less than 100 ng/mL). This finding is consistent with studies highlighting the low bioavailability of high molecular weight degraders;^30^ nevertheless, it remains a helpful finding for further optimization of our molecules.

## Discussion

In this manuscript, we describe the synthesis of a group of newly synthesized MYC degraders and extensively test their pharmacological activity in a comprehensive panel of assays. Initially, the stability of the new compounds was assessed in cell culture medium, confirming the stable nature of the synthesized compounds over a time frame of 72 h-incubation period. This was important as we attempted to decipher a new role for the described molecules that can be attributed to the intact molecule and not a degradant (metabolite) formed under the experimental conditions.

The candidate compounds were then evaluated for their potential to degrade the c-MYC protein. Most of the compounds tested indeed reduced MYC levels in PC3 cells, with VHL-based **CSI107** having distinguishable activity in comparison to the other candidate molecules (Figure 3A). Indeed, **CSI107** also appeared to be effective in MYC degradation in a dose-dependent manner (Figure 3B). Compound **CSI86** also displayed MYC degrading potential, while **CSI135**, **CSI90**, and **CSI95** did not show any particular effect in protein degradation in experiments performed in the same cell line. To further elucidate the involvement of VHL-mediated MYC degradation by the described compounds, a comparative study that involved treatment of PC3 cells with **CSI107** and its direct analogue **CSI212**, not bearing the E3-ligase binding features, was performed (Figure 3C). The results demonstrate how a small modification of the 4-hydroxy group from the *R* to *S* conformation (the “negative” VHL ligand, similar to the *trans*-VHL ligand) renders **CSI212** inactive toward MYC degradation. The hypothesis was that **CSI212** was unable to recruit E3-ligase in order to mark the POI and ultimately cause degradation via the proteasome, even though it also carries the same potent ligand (as **CSI107**) for targeting the MYC protein. Thus, the negative control **CSI212** confirms and strengthens our hypothesis that **CSI107** and the rest of the analogues that have been synthesized act according to the complex mechanism of E3-ligase-mediated ubiquitination. The inhibition of the molecule’s activity by the proteasome inhibitor **MG-132** further suggests that **CSI107** acts as a PROTAC, capable of targeting and degrading the MYC protein through the proteasome.

To untangle different aspects of the mechanistic potential of our new compounds, experiments were performed to investigate the capacity of MYC degraders to directly bind to MYC by microscale thermophoresis (MST) using the recombinant bHLH-MYC protein. As shown in Figure 4, all the compounds tested were found to directly bind to MYC. It is noteworthy that compounds **CSI86**, **CSI107**, and **CSI135** were equally potent in targeting MYC, with the established MYC binders **10058-F4** and **MYCi361** used as positive controls in our experiments.

To assess whether the new compounds affect MYC’s direct interaction with MAX, a pull-down approach was employed using recombinant proteins that correspond to the bHLHZ regions of MYC and MAX. Interestingly, only **CSI107** inhibited the binding of HisMAX to MYC, suggesting that this compound interacts with MYC at domain(s) involved in its binding to MAX, while the other degraders, such as **CSI86** and **CSI118**, do not significantly interfere with MYC–MAX complex formation (Figure 5). It should be noted that such an effect is not definitive, and further experiments are needed to decipher the mechanism of action.

Experiments regarding the evaluation of selectivity represent an extremely important part of drug discovery, and related studies are essential for the development of potent protein degraders. Relevant studies that elucidate the important selectivity properties of our newly synthesized molecules are currently ongoing and will constitute part of future work. Such studies will help us identify potential differences between the lead presented degraders (e.g., comparisons to **MYCi361**). Various proteomic and transcriptomic experiments in specific cell lines are in progress, aiming to clarify how our degraders might affect other proteins (except MYC). Such studies will unveil future opportunities for possible codosing schemes of the tested compounds or predict potential PROTAC-related toxic effects.

Apart from the mechanistic set of experiments, cell uptake experiments were used to investigate the ability of our compounds to enter the cell. The VHL- or pomalidomide-bearing derivatives exhibited reasonable cell uptake ranging from 4% to 24%. The data shown in Table 1 clearly indicated a significant uptake of **CSI86** by both SKBR3 and PC3 cells. Results for **CSI107** seem to be equally promising. Interestingly, **CSI135** exhibited lower penetrability compared to **CSI86** and **CSI107** (7% versus 19% and 13% in PC3 cells, respectively). The small molecule, **MYCi361**, had the relatively highest penetration in the cell lines tested (19% and 28% in the SKBR3 and PC3 cells, respectively).

The antiproliferative activity of the synthesized compounds was assessed via the MTT assay. Overall, the candidate pomalidomide-based molecule **CSI90** did not display any effect on cell proliferation in PC3 and SKBR3 cells. In contrast, the VHL-based degraders yielded pronounced effects in cell viability and proliferation inhibition, despite their “bulkier” structural features, with **CSI86**, **CSI107**, and **CSI135** showing higher potency (Table 2).

**Table 2:** Antiproliferative Effect of MYC-Targeted PROTACs in Different Cell Lines

Compound	Linker	E3 Ligase	Cell line	IC_50_ (μM)
**CSI90**	–COCH_2_N(CH_2_CH_2_)_2_N(CH_2_)_3_CO–	CRBN	SKBR3	inactive
			PC3	inactive
**CSI86**	–CO(CH_2_)_2_CO–	VHL	SKBR3	15 ± 4
			PC3	18 ± 7
			A549	inactive
**CSI107**	–CO(CH_2_)_3_CO–	VHL	SKBR3	14 ± 2
			PC3	13 ± 7
**CSI95**	–CO(CH_2_)_2_CONHCH_2_CO–	VHL	PC3	inactive
			A549	inactive
**CSI135**	–COCH_2_O(CH_2_)_2_OCH_2_CO–	VHL	PC3	25 ± 2
**CSI212**	–CO(CH_2_)_3_CO–	VHL	PC3	inactive
			SKBR3	inactive
**MYCi361**	-	-	SKBR3	11 ± 4
			PC3	8 ± 1

Based on our extensive in vitro set of experiments, we advanced our work with in vivo pharmacokinetic studies of the promising lead **CSI107** and the other degraders of interest, such as **CSI86** and **CSI135**. All three compounds showed reasonably high concentration in mouse blood after i.p. treatment (at 10 mg/kg), with evident levels of the degrader being present in blood circulation even at 24 h after treatment. Moreover, under the described dosing conditions, no adverse effects were reported in the duration of the study. The pharmacokinetic studies for the three molecules, **CSI86**, **CSI107**, and **CSI135**, suggested similar *C*_max_ and *T*_max_ values between the compounds, with some differences in AUCs (8.4, 3.4, 6 h × μg/mL, respectively), which may be attributed to the different structures of the linker and compound lipophilicity. We also proceeded with a PO dose study of **CSI86** in mice (10 mg/kg). The levels of the degrader in mouse blood that were monitored by LC-MS/MS were not appreciable (*C*_max_ less than 100 ng/mL). The findings of the study are in line with results that also indicate low bioavailability of high molecular weight degraders and provide useful guidance for further optimizations of our molecules.^31^

It should be noted that the previously described small molecules, **MYCi361** and **MYCi975**, generate high circulating levels following oral administration in mice. Our intent in this study was not to provide advantages over **MYCi361** and **MYCi975** but to use them as binding probes in novel protein degrader designs. Knowledge of larger molecules, such as those presented in this manuscript, that reduce tumorigenic activity via the ubiquitin–proteasome system is critical in the field of cancer research.

Overall, the comprehensive set of experiments employed herein revealed a valuable activity pattern of the VHL-based PROTACs and new opportunities toward the discovery of new anticancer drugs with promising pharmacological properties and biological impact on MYC elimination. Several thoughts occur with respect to the catalytic factor behind these observations. The two distinct E3 ligase ligand moieties used for the synthesis of our compounds may partly form a noticeable first answer. It is evident that the compounds bearing the CRBN recruiter have no actual effect on either in vitro experiments compared to the VHL-based ones. In the latter group, **CSI95**, bearing the −CO(CH_2_)_2_CONHCH_2_CO– linker, was also found relatively inactive when compared to the more prominent VHL-based compounds, highlighting the importance of the linker for the activity of the degrader. **CSI86**, **CSI107**, and **CSI135**, bearing −CO(CH_2_)_2_CO–, −CO(CH_2_)_3_CO–, and −COCH_2_O(CH_2_)_2_OCH_2_CO–, respectively, displayed similar antiproliferative effects, with **CSI107**, however, showing higher degrading capacity toward MYC. Further optimization of the linker substructure is clearly necessary to develop more effective MYC degraders and allow efficacy evaluation in clinically relevant animal models as the subject of future studies.

## Experimental Section

### Molecular Modeling

The crystal structure of the MYC protein (PDB ID: 1NKP)^24^ was retrieved from the Protein Data Bank. The protein and ligands were processed through AutoDockTools (version 1.5.6). Max protein, DNA, and water molecules were removed, the Gasteiger charges and polar hydrogen atoms were added, and the nonpolar hydrogens were merged. The compounds were designed and energy minimized employing the MM2 force field using the ChemBio3D Ultra 15.0. The pdbqt formats of MYC and ligands were prepared, and the molecular docking studies were performed using AutoDock Vina software.^32^ The compounds were docked to MYC via the AutoDock Vina (Vina) with exhaustiveness set to 8 or 100 and a docking grid of 74 × 74 × 38 Å for the *x*-, *y*-, and *z*-axes, respectively. The lowest binding energy was selected as the docked model. The figures were prepared using PyMOL 2.1.1 (Schrödinger, LLC) and Discovery Studio (BIOVIA).

### Compound Synthesis

The synthetic procedures for intermediate compounds and MYC degraders are reported in the Experimental Section or Supporting Information.

### Chemicals

**MG-132** (C2211) was purchased from Sigma-Aldrich (Munich, Germany), **MYCi361** (NUCC-0196361) from Selleckchem (TX, USA), and **BETd-260** from MedChemExpress (Monmouth, NJ, USA).

### Stability of PROTACs in Medium

The stability of most compounds, including **CSI86** PROTAC, was evaluated in DMEM. The drug was prepared at a concentration of 5 μM in DMEM (10% FBS, 1% pen/strep) and aliquoted at approximately 200 μL per Eppendorf. The samples were collected at appropriate time points (0, 1 h, 2 h, 4 h, 8 h, 24 h, 48 h, and 72 h) and stored at −80 °C. On the day of the LC-MS/MS analysis, samples were prepared (50 μL drug in DMEM + 50 μL ACN:H_2_O + 50 μL IS + 100 μL cold ACN), vortexed, sonicated, and centrifuged at 13 000 rpm for 15 min. Then, samples were transferred into glass tubes, diluted in the initial mobile phase (20% Α – 80% Β), centrifuged for 1 min, and added to a 96-well plate for the LC-MS/MS analysis.

### Western Blot

PC3 cells were used for the determination of MYC levels after treatment with the PROTACs under study. Cells (at a density of 250 000 per well) were seeded in 6-well plates for 24 h until 60% confluence. During the 24-h incubation period, cells were treated with the tested compounds (10 μΜ) and BETd-260 (1 μΜ) that was used as a positive control. For rescue experiments, cells were pretreated with the inhibitor **MG-132** for 2 h, and then, the medium was removed, followed by the addition of the selected compound. After the 24-h incubation period, a cell lysis protocol was performed. The medium was removed, and the cells were rinsed with cold PBS. Lysis was induced using RIPA lysis buffer (50 mM Tris-HCl at pH 7.5, 150 mM NaCl, 5 mM Na_2_EDTA, 1 mM EGTA, 0.1% SDS, 1% NP-40, 0.5% sodium deoxycholate, 8 mM sodium fluoride, and 1 mM sodium orthovanadate) and protease inhibitor cocktail (Roche, UK). Total protein concentration in lysate samples was determined via performance of the Bradford assay in bovine serum albumin (BSA). Standard and unknown samples were preformulated in Coomassie blue G-250 assay reagent (Thermo Fisher Scientific, MA, USA), followed by a readout at 595 nm. Separation of the protein isolated from PC3 lysates, respectively (10 μg), with NuPAGE sample reducing agent and NuPAGE LDS sample buffer (Invitrogen, UK), was achieved via SDS-PAGE electrophoresis at 110 V for 2 h, prior to overnight transfer to the polyvinylidene difluoride (PVDF) membrane. The membranes were then blocked in 5% nonfat dry milk in Tris-buffered saline with 0.1% Tween-20 (TBST-T) for 1 h at room temperature.

Detection of MYC in PC3 cells was accomplished via immunoblot of the POI with the indicated monoclonal antibody (Cell Signaling Technology, MA, USA, catalog number #5605) at 1:1000 in TBST at 2 °C overnight. The secondary anti-rabbit peroxidase-conjugate antibody (Cell Signaling Technology, MA, USA, catalog number #7074) was further added at 1:2000 dilution, followed by 1-h incubation at room temperature. Chemiluminescent visualization of the MYC protein bands was performed after the addition of Immobilon Crescendo Western HRP substrate (Sigma-Aldrich, Munich, Germany). For normalization of MYC concentration, GAPDH was labeled using the specific rabbit monoclonal antibody (Cell Signaling Technology, MA, USA, catalog number #5174) at 1:20000 in 5% nonfat dry milk. Protein bands were quantified with the ImageJ software.

### Statistical Analysis

The data from each independent experiment were analyzed via GraphPad Prism 6.01 (GraphPad Software, Inc.). Data are shown as the mean value ± SD of three biological replicates per experiment. Statistical significance was determined by the implementation of an unpaired *t*-test.

### Microscale Thermophoresis Assay

Microscale thermophoresis (MST) assay was used to determine whether PROTACs bind directly to MYC. The recombinant bHLHZ-Myc protein carrying an aminoterminal His_6_-tag was expressed in bacteria from a plasmid kindly provided by George Sianidis (BRFAA, Greece) and purified, as previously described,^33^ with minor modifications. It was subsequently labeled with a fluorescent dye using the protein labeling kit, His-Tag Labeling Kit RED-Tris-NTA Second Generation (NanoTemper Technologies, Germany). After a 30-min incubation followed by centrifugation, the protein samples were loaded into premium glass capillaries in triplicate (NanoTemper). PROTAC compounds, as well as **Myci361** and **10058-F4**, were dissolved in 100% DMSO, mixed with the labeled protein in the assay reaction buffer (PBS supplemented with 0.05% Tween-20 and DMSO at a final concentration of 2%), and the mixtures were loaded into capillaries in triplicates. Final concentrations were 25 nM for the protein and 200 μM for the compounds tested. MST was measured using a Monolith NT.115Pico with a red filter at an ambient temperature of 25 °C. The MST power and excitation power were adjusted to medium and 20%, respectively. Three experiments were carried out for each compound tested. Results were analyzed using the software provided with the Monolith instrument. Data are reported as ΔFnorm, which results from subtracting the normalized fluorescence value (Fnorm) of MYC in the absence of PROTAC from Fnorm in excess of PROTAC (bound state).

### In Vitro MYC–MAX Binding Assay

MYC–MAX complex formation assays were performed using recombinantly produced GST-MYC and His-MAX proteins, following the glutathione GST pull-down principle (Lentzaris et al., in preparation). In this assay, recombinant GST-MYC bound to glutathione beads was preincubated with the indicated compound or vehicle in the presence of His-MAX. As a negative control, empty beads, i.e., not loaded with GST-MYC, were used. The formed complex was eluted with glutathione, while the amount of bound MAX (indicating the amount of the generated complex) was assessed by Western blotting using anti-MAX antibodies (Abcam) and quantified based on the image J free software. All assays were carried out at least three independent times.

### Determination of Intracellular Concentrations of PROTACs

Cells were plated in 24-well plates at a density of 2 × 10^5^ cells/well. 24 h after seeding, cells were incubated with each PROTAC (10 μΜ) for certain time points (1 h, 4 h, 8 h, and 24 h). The incubations were terminated at the selected time points by removing the medium and washing the cells twice with 1 mL cold PBS (1×) in each well in order to remove unbound PROTAC. Subsequently, the cells were lysed by adding a cold solution of ACN:H_2_O (3:2) and scraping off the cell monolayer. This process was done in two steps: first, 200 μL of ACN:H_2_O was added, the cells were scraped, and the cell lysate was collected. The second step involved the addition of an extra 100 μL of ACN:H_2_O, scraping, and collection of the remaining lysate. Samples were subsequently vortexed, sonicated, and centrifuged for 10 min at 13 000 rpm. The supernatants were collected, evaporated for 1 h at 55 °C, and stored at −20 °C until the day of analysis. Samples were then reconstituted in 200 μL of initial mobile phase (100% A), and the intracellular accumulation of PROTACs was determined by LC-MS/MS analysis using Warfarin as a stable internal standard and PROTAC standards for the construction of analytical standard curves.

The percentage of cell uptake was calculated as follows:
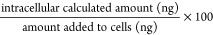


### Cell Cultures

In this study, three different cancer cell lines were used. The human lung adenocarcinoma cell line A549 (CRM-CCL-185) was obtained from the American Type Culture Collection (ATCC; VA, USA), and the human breast cancer cell line SKBR3 (CRM-HTB-30) and human prostate adenocarcinoma cell line PC3 (CRL-1435) were purchased from Cytion, Cell Line Service (CLS; Eppelheim, Germany). SKBR3 and A549 were cultured in Dulbecco’s modified Eagle’s medium (DMEM; Sigma-Aldrich, MA, USA), while the PC3 cell line was cultured in Dulbecco’s modified Eagle’s medium/Nutrient Mixture F-12 (DMEM/F12 1:1; PAN-Biotech, Germany). Both media were supplemented with 10% and 5% fetal bovine serum (FBS), respectively, and 1% antibiotics (penicillin/streptomycin 10 000 U/mL; Thermo Fisher Scientific, MA, USA) for the prevention of bacterial and mold growth. All cell lines were grown on T75 tissue culture flasks, incubated under a humidified atmosphere at 37 °C, 5% CO_2_. Passages for all cell lines were carried out using trypsin-EDTA (1×) solution (Thermo Fischer Scientific, MA, USA) to dissociate adherent cells, and passaging was performed every 3–4 days after approximately 80% cell confluence. All experimental parts were carried out with cells passaged no more than 8 times from frozen cell batches of passage 27 for all cell lines.

### Cell Viability Assay

Cells were plated at a density of 5 × 10^3^ cells per well on 96-well plates. After 24 h of incubation (37 °C, 5% CO_2_), the cell medium was removed, and compounds were added at selected concentrations (0.1–100 μM), followed by incubation for 72 h. Control cells were treated with 0.4% DMSO medium to match the conditions in the wells where drugs were added. After 72 h, the medium was removed, and the 3-(4,5-dimethylthiazol-2-yl)-2,5-diphenyltetrazolium bromide (MTT) solution (0.3 mg/mL in PBS) was added to cells for 3 h under no light conditions. The MTT solution was removed, and the formazan crystals were dissolved in 100 μL of DMSO. The optical density was measured at 570 nm with a reference wavelength of 690 nm using an absorbance microplate reader (SpectraMax 190, Molecular Devices, Sunnyvale, CA, USA). The half-maximal inhibitory concentration (IC_50_) was calculated based on a four-parameter logistic equation, generating a cell viability curve, using GraphPad Prism 6.01 (GraphPad Software, Inc.). Two concentrations of an established drug, Docetaxel (100 nM and 1 nM), were used in each experiment as a positive control. The resulting absorbance values were used instead of IC_50_ calculation. Each point was the result of three independent experiments performed in triplicate.

### Pharmacokinetic Study in Mice

Animals that were used for the study were male C57BL/6J (*n* = 7) at the age of 6–8 weeks (average weight = 20–25 g). Each PROTAC was administered at 10 mg/kg, and the dosing volume per animal was 200 μL. The vehicle for **CSI86**, **CSI107**, and **CSI35**, respectively, was corn oil/10% DMSO, and the administration was i.p. **CSI86** was also administered PO at the same dose using 0.5% carboxymethyl cellulose (CMC) as a vehicle in water. Whole blood from two control mice was used for the preparation of standard solutions.

A serial tail bleeding protocol was performed for the collection of blood samples. Blood samples (10 μL) were collected at selected time points (15 min, 30 min, 1 h, 2 h, 4 h, 6 h, 8 h, and 24 h) in tubes containing 40 μL of sodium citrate (0.1 M, pH 4.5) and stored at −80 °C until the day of the analysis.

Animal experimentation was approved by the competent Veterinary Service of the Prefecture of Athens in accordance with the Presidential Decree 56/2013, harmonizing the national legislation with the European Directive 2010/63 for the protection of animals used for scientific purposes (authorization protocol number: 1385926/27-12-2022).

For the calculation of *T*_1/2_, a noncompartmental pharmacokinetics analysis was used via the online application “Dash App Gallery Pharmacokinetics Calculator.”

## Supporting Information Available

Synthetic procedure of all compounds; ^1^H NMR, ^13^C NMR spectra, and HPLC analysis of intermediate compounds and PROTACs; Western blots (PDF)
