# Supplementary material for: Developing MYC Degraders Bearing the Von Hippel–Lindau Ligand to Target the “Undruggable” MYC
Source: ACS Pharmacol Transl Sci. 2024 Nov 15;7(12):3955–68. doi: 10.1021/acsptsci.4c00452 (PMC11650737; doi:10.1021/acsptsci.4c00452)
Supplement: Supplementary file 1 — pt4c00452_si_001.pdf [file pt4c00452_si_001.pdf]

SUPPORTING INFO

Developing Myc Degraders Bearing Von Hippel-Lindau Ligand to Target the "Undruggable" Myc

Christos Siokatas,[1#] Alexandra Lampropoulou,[2#] Alexandra Smina,[2#] Katerina Soupsana,[3] Martha Kontostathi,[3,4] Athina Vasiliki Karra,[3,4] Theodoros Karampelas,[2] Anastasia S. Politou,[3,4] Savvas Christoforidis,[3,4] Constantin Tamvakopoulos,[2]* and Vasiliki Sarli[1]*

Corresponding Authors

[1] Vasiliki Sarli - Department of Chemistry, Aristotle University of Thessaloniki, University Campus, 54124 Thessaloniki, Greece; Orcid https://orcid.org/0000-0002-6128-8277; Email: sarli@chem.auth.gr

[2] Constantin Tamvakopoulos - Center of Clinical Research, Experimental Surgery and Translational Research, Division of Pharmacology-Pharmacotechnology, Biomedical Research Foundation, Academy of Athens, Soranou Ephessiou Street 4, Athens GR-11527, Greece;
Orcid https://orcid.org/0000-0001-9627-4812; Email: ctamvakop@bioacademy.gr

[3] Laboratory of Biological Chemistry, Department of Medicine, School of Health Sciences, University of Ioannina, 45110 Ioannina, Greece.

[4] Biomedical Research Institute, Foundation for Research and Technology, 45110 Ioannina, Greece.

[#] Authors contributed equally to this manuscript.

Supporting Info		
1	Compound Synthesis	S-2
2	^{1}H-NMR and ^{13}C-NMR spectra for compound **S6**	S-13
3	HMBC spectra for compound **S6**	S-14
4	^{1}H-NMR and ^{13}C-NMR spectra for compound **S7**	S-15
5	^{1}H-NMR and ^{13}C-NMR spectra for compound **CSI63**	S-16
6	^{1}H-NMR and ^{13}C-NMR spectra for compound **CSI90**	S-17
7	^{1}H-NMR and ^{13}C-NMR spectra for compound **8**	S-18
8	^{1}H-NMR and ^{13}C-NMR spectra for compound **CSI86**	S-19
9	^{1}H-NMR and ^{13}C-NMR spectra for compound **9**	S-20
10	^{1}H-NMR and ^{13}C-NMR spectra for compound **11**	S-21
11	^{1}H-NMR and ^{13}C-NMR spectra for compound **12**	S-22
12	^{1}H-NMR and ^{13}C-NMR spectra for compound **CSI95**	S-23
13	^{1}H-NMR and ^{13}C-NMR spectra for compound **15**	S-24
14	^{1}H-NMR and ^{13}C-NMR spectra for compound **16**	S-25
15	^{1}H-NMR and ^{13}C-NMR spectra for compound **17**	S-26
16	^{1}H-NMR and ^{13}C-NMR spectra for compound **CSI35**	S-27
17	^{1}H-NMR and ^{13}C-NMR spectra for compound **19**	S-28
18	^{1}H-NMR and ^{13}C-NMR spectra for compound **CSI212**	S-29
19	LC-MS Data of the synthesized compounds	S-30
20	Characterization and Quantitative Analysis of PROTACs by (LC-MS/MS)	S-41
21	Detection protein degraders in mouse blood by LC-MS/MS analysis	S-41
22	MYC Degradation following treatment with PROTACs in PC3 Cell Lines	S-42

Experimental Section

Compound Synthesis

Chemicals and solvents were purchased and used without further purification unless stated. Reactions were monitored by thin layer chromatography on silica gel plates and the visualization of the plates was carried out using a UV lamp. Ethanolic *p*-anisaldehyde solution, aqueous ceric sulfate/phosphomolybdic acid and heat were used as developing agents. NMR spectra were recorded on an Agilent 500 spectrometer with ^{1}H at 500 MHz and ^{13}C at 126 MHz, using the TMS internal standard. Chemical shifts are given in δ values (ppm) referenced to $CDCl_3$ with 7.26 for ^{1}H and 77.10 for ^{13}C, and DMSO-$d_6$with 2.50 for ^{1}H and 39.51 for ^{13}C, acetone-d_6 with 2.05 for ^{1}H and 206.26 for ^{13}C. Coupling constants (*J*) are reported in Hz. HPLC purification using C18 analytical Reprospher 100 (C18-DE, 5 µm, 250 × 10 mm, Dr Maisch GmbH) column was performed with Scientific Systems, Inc instrumentation comprising of 4-Q Grad Pumps connected to diode array (UV-Vis Thermo Finnigan Spectra system UV6000LP Detector, Lab Alliance [NY, USA]). High-resolution mass spectra (HRMS) spectra were recorded on a Bruker® Maxis Impact QTOF spectrometer.

- ***Synthesis of MYC binder***

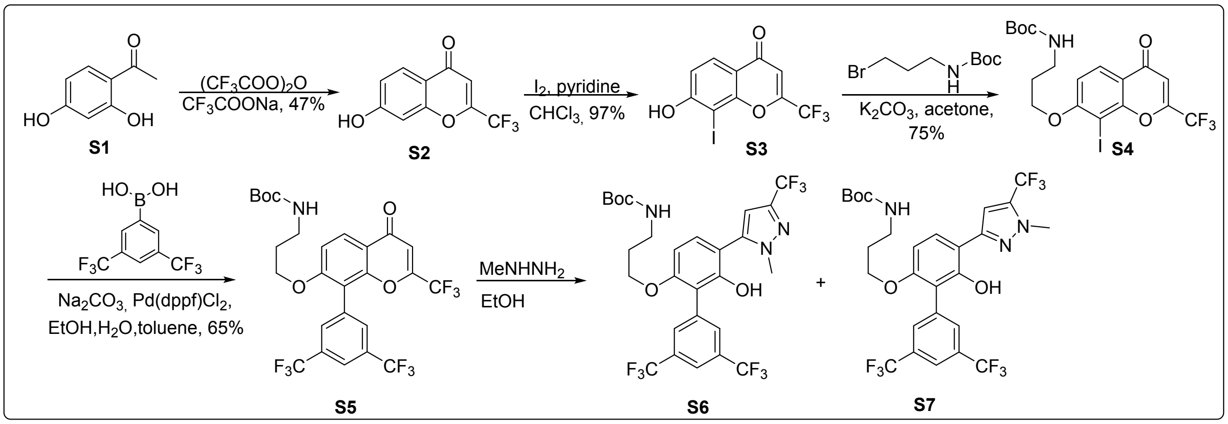

Scheme S1

Synthesis of 7-hydroxy-2-(trifluoromethyl)-4*H*-chromen-4-one, S2: To a stirred solution of 2,4-dihydroxy acetophenone (2.1 g, 13.8 mmol) in trifluoroacetic anhydride (6.73 mL, 48.4 mmol), dry pyridine (1.12 mL, 13.8 mmol) was added at 0° C under inert atmosphere. The mixture was allowed to warm up to room temperature and then stirred overnight at 80° C. The reaction was cooled down to room temperature, treated with H_2O and extracted with EtOAc. The combined organic phases were dried over Na_2SO_4 and solvent was removed under reduced pressure. The resulting residue was purified by silica gel chromatography (toluene: EtOAc=6:1 to 100% EtOAc) to give the desired product as a yellow solid (1.5 g, 47%).[1]

Synthesis of 7-hydroxy-8-iodo-2-(trifluoromethyl)-4*H*-chromen-4-one, S3: To a solution 7-hydroxy-2-(trifluoromethyl)-4*H*-chromen-4-one (1.2 g, 5.21 mmol) in $CHCl_3$ (20 mL), dry pyridine (1.69 mL, 20.9 mmol) was added, followed by the addition of iodine (5.3g, 20.9 mmol) and the reaction mixture was

stirred overnight at room temperature under inert atmosphere. The completion of the reaction was confirmed via TLC and the excess iodine was quenched with saturated aq. $Na_2S_2O_3$. The organic layer was separated, and the aqueous layer was washed with DCM. The combined organic layers were dried over anhydrous Na_2SO_4, filtered and the solvents were removed to give the desired product as an off-white solid (1.8 g, 97%). The spectral data were in accordance with those reported in the literature.[1] ^{1}H NMR (500 MHz, $CDCl_3$) δ 8.12 (d, *J* = 8.8 Hz, 1H), 7.14 (d, *J* = 8.8 Hz, 1H), 6.73 (s, 1H), 5.30 (s, 1H).

Synthesis of *tert*-butyl (3-((8-iodo-4-oxo-2-(trifluoromethyl)-4*H*-chromen-7-yl)oxy)propyl)carbamate, S4: In a self-sealing tube containing 7-hydroxy-8-iodo-2-(trifluoromethyl)-4*H*-chromen-4-one (1.8 g, 5.06 mmol) as a suspension in dry acetone (25 mL), K_2CO_3 (1.26 g, 9.1 mmol) was added under inert atmosphere, followed by the addition of 3-(boc-amino)propyl bromide (2.17 g, 9.1 mmol). The reaction was stirred under reflux conditions overnight. Water was added and the reaction mixture was extracted with EtOAc. The combined organic layers were dried over Na_2SO_4, filtered and solvents were removed under reduced pressure. The crude residue was purified by silica gel chromatography (*n*-hexanes: EtOAc= 10:1 to 100% EtOAc) to give the desired product as a yellow oil (1.95 g, yield 75%). The spectral data were in accordance with those reported in the literature.[1] ^{1}H NMR (500 MHz, $CDCl_3$) δ 8.17 (d, *J* = 8.9 Hz, 1H), 6.98 (d, *J* = 8.9 Hz, 1H), 6.71 (s, 1H), 5.02 (s, 1H), 4.26 (t, *J* = 5.8 Hz, 2H), 3.44 (dt, *J* = 12.2, 5.9 Hz, 4H), 1.44 (s, 9H); ^{13}C NMR (126 MHz, $CDCl_3$) δ 176.2, 163.4, 156.4, 156.2, 152.9, 128.0, 119.7, 119.1, 110.8, 110.6, 68.7, 29.8, 28.6, 28.6.

Synthesis of *tert*-butyl (3-((8-(3,5-bis(trifluoromethyl)phenyl)-4-oxo-2-(trifluoromethyl)-4*H*-chromen-7-yl)oxy)propyl)carbamate, S5: In a flask containing *tert*-butyl (3-((8-iodo-4-oxo-2-(trifluoromethyl)-4*H*-chromen-7-yl)oxy)propyl)carbamate (1.2 g, 2.34 mmol) as a solution in a 1:2:6 mixture of EtOH:H_2O:toluene (18 mL), Na_2CO_3 (496 mg, 4.68 mmol) was added, followed by the addition of 3,5-bis(trifluoromethyl)phenyl boronic acid (722 mg, 2.99 mmol) and Pd(dppf)Cl_2 and the resulting suspension was bubbled with Ar for 10 minutes. The reaction mixture was heated at 100° C overnight, under an atmosphere of Ar. Upon completion it was allowed to cool down to room temperature, and the mixture was diluted in EtOAc and washed with H_2O. The combined organic phases were filtered through celite and the crude dark-brown residue was purified by silica gel chromatography (n-hexanes: EtOAc=8:1 to 100% EtOAc) to give the desired product as a light-orange oil (910 mg, yield 65%).

Synthesis of *tert*-butyl (3-((6-hydroxy-5-(1-methyl-3-(trifluoromethyl)-1*H*-pyrazol-5-yl)-3',5'-bis(trifluoromethyl)-[1,1'-biphenyl]-2-yl)oxy)propyl)carbamate, S6: In a self-sealing tube containing tert-butyl (3-((8-(3,5-bis(trifluoromethyl)phenyl)-4-oxo-2-(trifluoromethyl)-4*H*-chromen-7-yl)oxy)propyl)carbamate (643 mg, 1.07 mmol) as a suspension in absolute ethanol (9.00 mL), methylhydrazine (170 µL, 3.23 mmol) was added and the reaction was stirred at 80° C for 45 minutes. The reaction was monitored via TLC. Upon completion, solvent was evaporated, and the crude residue was purified by silica gel chromatography (n-hexanes: EtOAc=8:1 gradient to n-hexanes: EtOAc=1:1). **S6:** ^{1}H NMR (500 MHz, $CDCl_3$): δ 7.93 (s, 2H), 7.87 (s, 1H), 7.22 (d, *J* = 8.6 Hz, 1H), 6.70 (d, *J* = 8.6 Hz, 1H), 6.59 (s, 1H), 4.50 (broad s, 1H), 4.03 (t, *J* = 6.0 Hz, 2H), 3.83 (s, 3H), 3.10 (t, *J* = 4.7 Hz, 2H), 1.85 (q, 2H), 1.39 (s, 9H). 1 proton missing (-OH)**;** ^{13}C NMR (126 MHz, $CDCl_3$): δ 158.1, 156.1, 151.6, 142.6, 142.1, 141.6, 139.7, 134.9, 131.9, 131.7, 131.6, 131.4, 131.2, 123.4, 121.5, 121.1, 115.5, 109.6, 105.6, 104.9, 79.6, 66.5, 37.9, 37.5, 29.7, 28.4 (1 carbon missing due to overlapping). **S7**: ^{1}H NMR (500 MHz, $CDCl_3$): δ 10.68 (brs, 1H),

7.98 (s, 2H), 7.82 (s, 1H), 7.53 (d, *J* = 8.7 Hz, 2H), 6.94 (s, 1H), 6.62 (d, *J* = 8.7 Hz, 1H), 4.50 (brs, 1H), 4.05 – 3.98 (m, 5H), 3.12 (d, *J* = 6.0 Hz, 2H), 1.90 – 1.82 (m, 2H), 1.41 (s, 9H); ^{13}C NMR (126 MHz, $CDCl_3$): δ 156.9, 156.1, 154.1, 150.3, 136.1, 133.5, 133.2, 131.8, 130.8, 127.5, 123.4, 121.4, 120.6, 109.9, 103.8, 103.7, 79.4, 66.4, 38.2, 37.7, 29.7, 28.5.

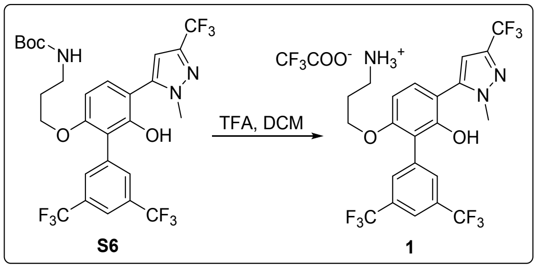

Scheme S2

Synthesis of 3-((6-hydroxy-5-(1-methyl-3-(trifluoromethyl)-1*H*-pyrazol-5-yl)-3',5'-bis(trifluoromethyl)-[1,1'-biphenyl]-2-yl)oxy)propan-1-aminium 2,2,2-trifluoroacetate, 1: S6 (30 mg, 0.048 mmol) was dissolved in DCM (0.8 mL) and TFA (80 µL) was added. The reaction stirred for 4h at room temperature and upon completion, monitored via TLC, the volatiles were removed under reduced pressure and the crude product was used into the next step without any further purification.

- **Synthesis of CSI63:**

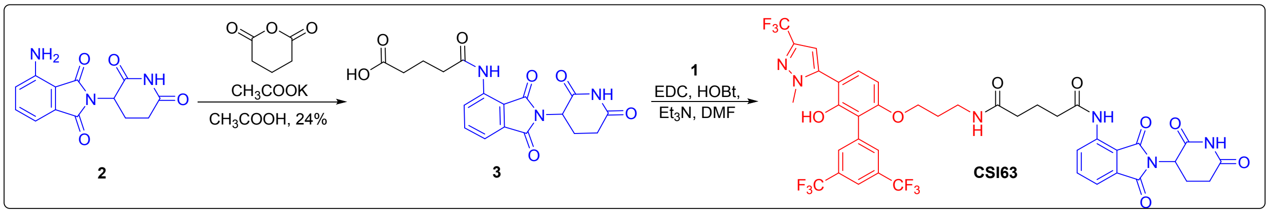

Scheme S3

Synthesis of 5-((2-(2,6-dioxopiperidin-3-yl)-1,3-dioxoisoindolin-4-yl)amino)-5-oxopentanoic acid, 3: In a flask containing 4-amino-2-(2,6-dioxopiperidin-3-yl)isoindoline-1,3-dione (50 mg, 0.183 mmol) as a suspension in acetic acid (1.44 mL), potassium acetate (71.8 mg, 0.732 mmol) was added followed by the addition of glutaric anhydride (80.8 mg, 0.708 mmol) and the reaction mixture was stirred under reflux for 3h. After cooling at room temperature, acetic acid was removed under reduced pressure and the residue was extracted with (EtOAc – H_2O). The organic phases were collected, dried with $MgSO_4$, filtered and solvents were removed under reduced pressure. The crude product was purified by silica gel column chromatography (2.5-10% MeOH in EtOAc) to give the desire product as an off-white solid (17mg, yield=24%). The spectral data were in accordance with those reported in the literature.[2] **3**: ^{1}H NMR (500 MHz, DMSO-d_6) δ 12.09 (s, 1H), 11.14 (s, 1H), 9.72 (s, 1H), 8.44 (d, *J* = 8.4 Hz, 1H), 7.99 – 7.75 (m, 1H), 7.62 (d, *J* = 7.2 Hz, 1H), 5.14 (dd, *J* = 12.8, 5.4 Hz, 1H), 2.89 (ddd, *J* = 17.3, 14.1, 5.5 Hz, 1H), 2.65 – 2.52 (m, 4H), 2.31 (t, *J* = 7.3 Hz, 2H), 2.10 – 2.02 (m, 1H), 1.89 – 1.80 (m, 2H); ^{13}C NMR (126 MHz, DMSO-d_6) δ 174.2,

172.7, 171.6, 169.8, 167.6, 166.6, 136.4, 136.1, 131.5, 126.5, 118.4, 117.2, 48.9, 35.6, 32.9, 30.9, 22.0, 20.2.

Synthesis of *N*1-(2-(2,6-dioxopiperidin-3-yl)-1,3-dioxoisoindolin-4-yl)-*N*5-(3-((6-hydroxy-5-(1-methyl-3-(trifluoromethyl)-1*H*-pyrazol-5-yl)-3',5'-bis(trifluoromethyl)-[1,1'-biphenyl]-2-yl)oxy)propyl) glutaramide, CSI63: In a flask containing **1** (17.8 mg, 0.0284 mmol) as a solution in dry DMF (700 µL) and under inert atmosphere, triethylamine (22 µL, 0.158 mmol) was added followed by the addition of EDC hydrochloride (7.42 mg, 0.0387 mmol), HOBt (5.23 mg, 0.0387 mmol) and finally **3** (10 mg, 0.0258 mmol). The reaction was stirred overnight at room temperature. Volatiles were removed under reduced pressure and the crude residue was purified with silica gel chromatography (2-8% MeOH in DCM) to give the desired product as a white powder (8 mg, yield 35%). **CSI63:** ^{1}H NMR (500 MHz, acetone-d_6) δ 9.99 (s, 1H), 9.49 (s, 1H), 8.77 (d, *J* = 8.5 Hz, 1H), 8.15 (s, 2H), 7.96 (d, *J* = 20.3 Hz, 1H), 7.81 (t, *J* = 7.9 Hz, 1H), 7.55 (d, *J* = 7.3 Hz, 1H), 7.32 (d, *J* = 8.6 Hz, 1H), 7.19 (t, *J* = 5.1 Hz, 1H), 6.84 (d, *J* = 8.6 Hz, 1H), 6.62 (s, 1H), 5.15 (dd, *J* = 12.5, 5.3 Hz, 1H), 4.10 (t, *J* = 5.9 Hz, 2H), 3.82 (s, 3H), 3.25 (dd, *J* = 12.4, 6.2 Hz, 2H), 2.94 (s, 1H), 2.77 (s, 1H), 2.57 (t, *J* = 7.3 Hz, 3H), 2.25 (t, *J* = 10.0 Hz, 3H), 2.03 – 1.95 (m, 2H), 1.85 – 1.78 (m, 2H); ^{13}C NMR (126 MHz, acetone-d_6) δ 172.5, 172.5, 169.9, 169.8, 167.5, 162.8, 159.0, 153.7, 142.3, 141.2, 138.6, 137.5, 136.9, 133.3, 133.1, 133.0, 132.5, 131.6, 123.6, 121.7, 121.5, 118.5, 116.9, 116.7, 111.6, 106.2, 106.2, 105.3, 79.2, 66.7, 50.3, 38.1, 37.2, 36.2, 36.1, 35.5, 31.9, 31.0, 23.3, 21.9; ESI-HRMS m/z for $C_{40}H_{33}F_9N_6NaO_8^+$ [M + Na]$^+$ calculated 919.2108; found 919.2099.

➢ Synthesis of CSI90

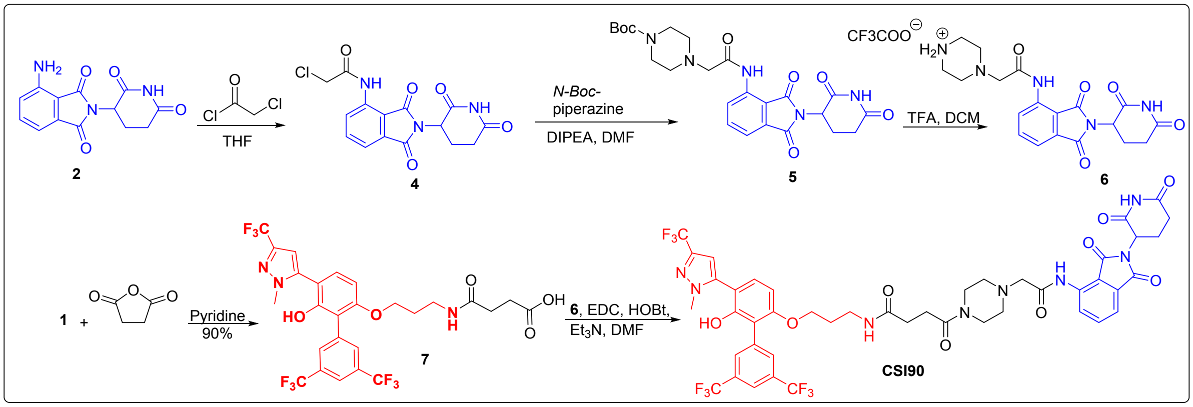

Scheme S4

Synthesis of 2-chloro-*N*-(2-(2,6-dioxopiperidin-3-yl)-1,3-dioxoisoindolin-4-yl)acetamide, 4: In a self-sealing tube containing 4-amino-2-(2,6-dioxopiperidin-3-yl)isoindoline-1,3-dione (160 mg, 0.589 mmol) as a suspension in THF (3 mL), chloroacetyl chloride (52 µL, 0.648 mmol) was added under inert atmosphere and the reaction mixture was heated under reflux conditions for 30 minutes. Solvent was removed under reduced pressure and the obtained solid was suspended in Et_2O and filtered to afford the desired product as a yellowish solid which was used onto the next step without any further purification (183 mg, yield 89%). The spectral data were in accordance with those reported in the literature.[2] **4**: ^{1}H NMR (500 MHz, DMSO-d$_6$) δ 11.15 (s, 1H), 10.31 (s, 1H), 8.54 (d, *J* = 8.4 Hz, 1H), 7.88 (t, *J* = 7.8 Hz, 1H), 7.68 (d, J = 7.2 Hz,

1H), 5.17 (dd, J = 12.8, 5.3 Hz, 1H), 4.53 (s, 2H), 2.94 – 2.85 (m, 1H), 2.59 (dd, J = 23.9, 14.1 Hz, 2H), 2.11 – 2.03 (m, 1H); ^{13}C NMR (126 MHz, DMSO-d_6) δ 172.7, 169.7, 167.8, 166.6, 165.7, 136.4, 135.6, 131.5, 125.6, 119.0, 117.3, 48.9, 43.1, 30.9, 21.9.

Synthesis of *tert*-butyl 4-(2-((2-(2,6-dioxopiperidin-3-yl)-1,3-dioxoisoindolin-4-yl)amino)-2-oxoethyl)piperazine-1-carboxylate, 5: In a flask containing 2-chloro-*N*-(2-(2,6-dioxopiperidin-3-yl)-1,3-dioxoisoindolin-4-yl)acetamide (20 mg, 0.057 mmol) as a solution in dry DMF (800 µL) and under inert atmosphere, DIPEA (27 µL, 0.157 mmol) was added followed by the addition of 1-Boc-piperazine (9.77 mg, 0.052 mmol). The reaction was stirred overnight at 60° C. Solvent was removed under reduced pressure and the crude material was purified via silica gel chromatography (DCM 12:1 Acetone gradient to 6:1) to give the desired product as off-white solid (18.2 mg, yield 64%).[3] **5**: ^{1}H NMR (500 MHz, Acetone-d_6) δ 11.20 (broad s, 1H), 9.95 (broad s, 1H), 8.88 (d, J = 8.5 Hz, 1H), 7.83 (t, J = 7.9 Hz, 1H), 7.56 (d, J = 7.3 Hz, 1H), 5.19 (dd, J = 12.8, 5.4 Hz, 1H), 3.59 (s, 2H), 3.05 – 2.96 (m, 2H), 2.86 – 2.77 (m, 4H), 2.63 (m, 4H), 2.27 (m, 2H), 1.46 (s, 9H).

Synthesis of *N*-(2-(2,6-dioxopiperidin-3-yl)-1,3-dioxoisoindolin-4-yl)-2-(piperazin-1-yl)acetamide, 6: Compound **5** (18.7 mg, 0.037 mmol) was treated with a 1:3 mixture of TFA:DCM (0.8 mL) for 2 hours. Upon completion of the deprotection step (confirmed via TLC), the volatiles were removed under reduced pressure and compound **6** was used to the next step without further purification.

Synthesis of 4-((3-((6-hydroxy-5-(1-methyl-3-(trifluoromethyl)-1*H*-pyrazol-5-yl)-3',5'-bis(trifluoromethyl)-[1,1'-biphenyl]-2-yl)oxy)propyl)amino)-4-oxobutanoic acid, 7: In a flask containing compound **1** (0.048 mmol), dry pyridine (300 µL) was added under inert atmosphere, followed by the addition of succinic anhydride (9.7 mg, 0.097 mmol) and the reaction stirred overnight at room temperature. Solvent was removed and the crude mixture was purified via silica gel chromatography (2-5% MeOH in EA) to give the desired product as a white solid (27.4 mg, 90%). **7:** ^{1}H NMR (500 MHz, acetone-d_6) δ 8.13 (s, 2H), 7.99 (s, 1H), 7.34 (d, J = 8.6 Hz, 1H), 7.26 (broad s, 1H), 6.86 (d, J = 8.6 Hz, 1H), 6.64 (s, 1H), 4.11 (t, J = 6.1 Hz, 2H), 3.84 (s, 3H), 3.24 (q, J = 6.3 Hz, 2H), 2.55 (t, J = 6.8 Hz, 2H), 2.42 (t, J = 6.8 Hz, 2H), 2.05 (m, 2H); ^{13}C NMR (126 MHz, acetone-d_6) δ 173.9, 172.5, 159.0, 153.7, 150.5, 142.3, 137.5, 133.3, 133.0, 131.6, 125.8, 123.6, 121.5, 116.9, 111.6, 106.2, 105.4, 66.7, 38.1, 36.3, 31.1 (2 carbons are missing due to overlapping with solvent); ESI-MS m/z for $C_{26}H_{22}F_9N_3O_5$ $[M+H]^+$, calculated 628.15; found 627.8**.**

Synthesis of 4-(4-(2-((2-(2,6-dioxopiperidin-3-yl)-1,3-dioxoisoindolin-4-yl)amino)-2-oxoethyl)piperazin-1-yl)-*N*-(3-((6-hydroxy-5-(1-methyl-3-(trifluoromethyl)-1*H*-pyrazol-5-yl)-3',5'-bis(trifluoromethyl)-[1,1'-biphenyl]-2-yl)oxy)propyl)butanamide, CSI90: Compound **6** (0.037 mmol) was dissolved in dry DMF (1mL). Then, triethylamine (24 µL, 0.17 mmol) was added, followed by the addition of EDC hydrochloride (9.79 mg, 0.051 mmol), HOBt (7.82 mg, 0.051 mmol) and compound **7** (21.4 mg, 0.034 mmol) and the reaction was stirred under inert atmosphere overnight at room temperature. Volatiles were evaporated and the crude residue was purified by silica gel chromatography (2-10% MeOH in CH_2Cl_2) to give the desired product as a white powder (25 mg, yield 73%). **CSI90:** ^{1}H NMR (500 MHz, acetone-d_6) δ 11.24 (broad s, 1H), 9.99 (broad s, 1H), 8.87 (d, J = 8.4 Hz, 1H), 8.14 (s, 2H), 8.00 (d, J = 9.9 Hz, 1H), 7.83 (t, J = 7.9 Hz, 1H), 7.57 (d, J = 7.2 Hz, 1H), 7.35 (d, J = 8.5 Hz, 1H), 7.22-7.17 (m, 1H), 6.88 (d, J = 8.6 Hz, 1H), 6.64 (s, 1H), 5.18 (dd, J = 12.7, 5.3 Hz, 1H), 4.12 (t, J = 6.1 Hz, 2H), 3.83 (s, 3H), 3.72 (s, 4H), 3.25 (d, J = 7.4 Hz,

4H), 2.86 – 2.73 (m, 3H), 2.63 (d, *J* = 6.4 Hz, 3H), 2.41 (t, *J* = 6.6 Hz, 2H), 1.96 (s, 4H), 1.83 (dd, *J* = 12.4, 6.2 Hz, 2H); ^{13}C NMR (126 MHz, acetone-d_6) δ 172.8, 172.6, 172.1, 170.9, 170.6, 169.9, 169.4, 167.7, 159.0, 153.6, 142.3, 141.3, 138.0, 137.5, 136.9, 133.3, 133.0, 132.7, 131.6, 125.1, 124.7, 124.1, 121.5, 118.5, 117.0, 116.9, 111.5, 106.2, 105.5, 79.2, 66.7, 62.3, 54.1, 53.8, 50.3, 45.9, 42.3, 38.2, 36.2, 32.0, 31.9, 31.6, 23.3, 20.5; ESI-HRMS m/z for $C_{45}H_{41}F_9N_8NaO_9^+$ $[M+Na]^+$ calculated 1031.2745; found: 1031.2732.

➢ Synthesis of CSI86

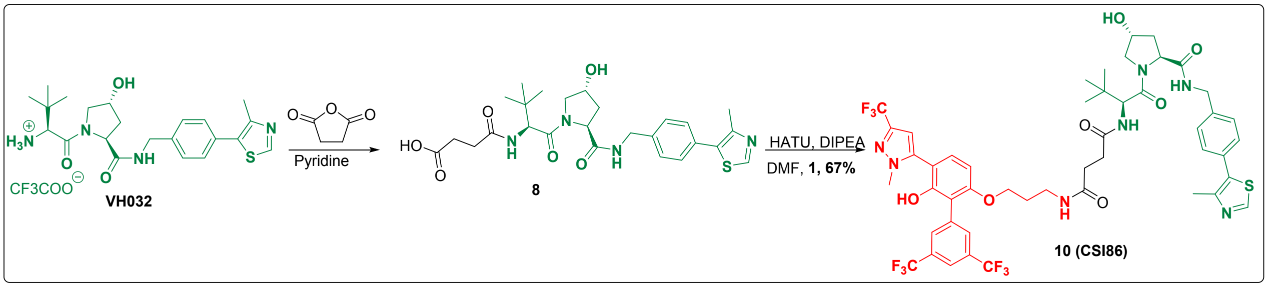

Scheme S5

Synthesis of 4-(((S)-1-((*2S,4R*)-4-hydroxy-2-((4-(4-methylthiazol-5-yl)benzyl)carbamoyl)pyrrolidin-1-yl)-3,3-dimethyl-1-oxobutan-2-yl)amino)-4-oxobutanoic acid, 8: In a flask containing (*S*)-1-((2*S*,4*R*)-4-hydroxy-2-((4-(4-methylthiazol-5-yl)benzyl)carbamoyl)pyrrolidin-1-yl)-3,3-dimethyl-1-oxobutan-2-aminium 2,2,2-trifluoroacetate (50 mg, 0.092 mmol) as a solution in dry pyridine (200 µL) and under inert atmosphere, succinic anhydride (18 mg, 0.184 mmol) was added and the reaction stirred overnight at room temperature. Pyridine was removed under reduced pressure and the crude mixture was purified via silica gel chromatography (5-20% MeOH in EtOAc) to give the desire product as a white solid (32 mg, yield 66%). **8:** ^{1}H NMR (500 MHz, acetone-d_6) δ 8.85 (s, 1H), 7.89 (t, *J* = 6.0 Hz, 1H), 7.47 (d, *J* = 8.2 Hz, 2H), 7.41 (d, *J* = 8.3 Hz, 2H), 7.37 (d, *J* = 9.0 Hz, 1H), 4.67 – 4.61 (m, 3H), 4.59 – 4.53 (m, 2H), 4.35 (dd, *J* = 15.5, 5.4 Hz, 1H), 3.89 (d, *J* = 10.3 Hz, 1H), 3.75 (dd, *J* = 10.8, 4.1 Hz, 1H), 2.65 – 2.49 (m, 5H), 2.47 (s, 3H), 2.17 (dd, *J* = 8.1, 3.9 Hz, 2H), 1.00 (s, 9H); ^{13}C NMR (126 MHz, acetone-d_6) δ 173.6, 171.7, 171.5, 170.6, 150.5, 148.2, 139.5, 131.5, 130.4, 128.9, 128.3, 127.8, 69.8, 59.2, 57.2, 56.6, 42.3, 37.5, 35.4, 30.1, 25.9, 15.4.

Synthesis of *N*1-((*S*)-1-((*2S,4R*)-4-hydroxy-2-((4-(4-methylthiazol-5-yl)benzyl)carbamoyl)pyrrolidin-1-yl)-3,3-dimethyl-1-oxobutan-2-yl)-*N*4-(3-((6-hydroxy-5-(1-methyl-3-(trifluoromethyl)-1*H*-pyrazol-5-yl)-3',5'-bis(trifluoromethyl)-[1,1'-biphenyl]-2-yl)oxy)propyl)succinimide, CSI86: To a stirred solution of compound **1** (29.3 mg, 0.046 mmol), in dry DMF (700 µL), DIPEA (43 µL, 0.249 mmol) was added, followed by the addition of HATU (23.6 mg, 0.0622 mmol) and compound **8** (22 mg, 0.0415 mmol). The reaction was stirred overnight at room temperature. Volatiles were removed and the crude residue was purified by silica gel chromatography (2-20% MeOH in EtOAc) to give the desire product as a white powder (29 mg, yield 67%). **CSI86:** ^{1}H NMR (500 MHz, acetone-d_6) δ 8.82 (s, 1H), 8.23 (broad s, 1H), 8.09 (s, 2H), 8.02 (d, *J* = 5.5 Hz, 1H), 7.97 (s, 1H), 7.79 (broad s, 1H), 7.42 (d, *J* = 8.2 Hz, 2H), 7.39 (d, *J* = 8.3 Hz, 2H), 7.33 (d, *J* = 8.6 Hz, 1H), 6.85 (d, *J* = 8.6 Hz, 1H), 6.63 (broad s, 1H), 4.81 (t, *J* = 7.9 Hz, 1H), 4.61 – 4.59 (m, 1H), 4.53 (s, 1H), 4.43 (d, *J* = 4.5 Hz, 2H), 4.08 (t, *J* = 6.1 Hz, 3H), 3.82 (s, 3H), 3.72 (dd, *J* = 11.0, 3.6 Hz, 2H), 3.32 (dd, *J* = 13.8, 5.2 Hz, 2H), 3.22 – 3.16 (m, 2H), 2.64 (t, *J* = 10.6 Hz, 2H), 2.45 (s, 3H), 2.16 (d, *J* = 7.6 Hz, 2H), 1.96 (s, 3H), 1.84 – 1.80 (m, 2H), 1.01 (s, 9H); ^{13}C NMR (126 MHz, acetone-d_6) δ 174.3, 174.0, 172.5, 172.3,

161.8, 158.9, 153.6, 151.3, 149.1, 142.3, 141.3, 140.4, 137.5, 133.3, 132.9, 132.3, 131.7, 131.5, 131.3, 129.9, 128.7, 127.9, 125.9, 121.7, 121.5, 118.8, 116.9, 116.5, 111.6, 106.2, 105.4, 79.2, 70.8, 66.7, 60.0, 59.9, 59.1, 59.0, 57.7, 57.5, 43.2, 38.3, 38.1, 36.6, 35.6, 26.9, 16.3 ESI-HRMS m/z for $C_{48}H_{51}F_9N_7O_7S$ $[M+H]^+$, calculated 1040.3427; found 1040.3412.

- **Synthesis of CSI107**

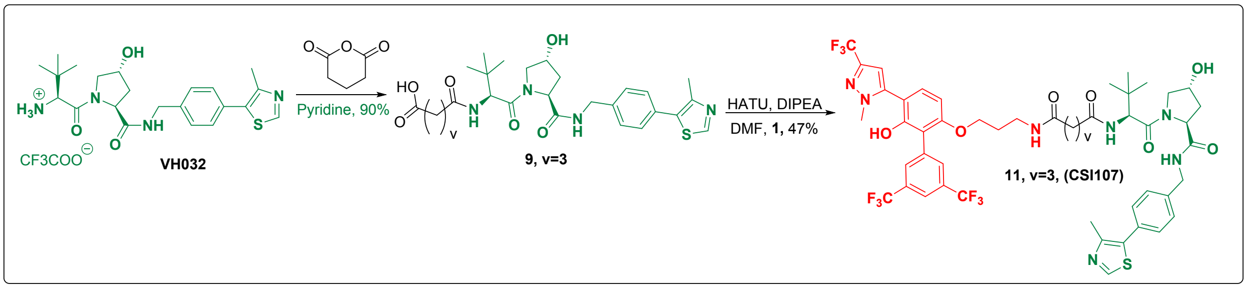

Scheme S6

Synthesis of 5-(((*S*)-1-((*2S,4R*)-4-hydroxy-2-((4-(4-methylthiazol-5-yl)benzyl)carbamoyl)pyrrolidin-1-yl)-3,3-dimethyl-1-oxobutan-2-yl)amino)-5-oxopentanoic acid, 9: In a flask containing compound **VH032** (18.6 mg, 0.034 mmol) as a solution in dry pyridine (300 µL) and under inert atmosphere, glutaric anhydride (7.79 mg, 0.068 mmol) was added and the reaction stirred overnight at room temperature. Pyridine was removed under reduced pressure and the crude mixture was purified via silica gel chromatography (2-10% MeOH in EtOAc) to give the desire product as a white solid (16.7 mg, yield 90%). **9**: ^{1}H NMR (500 MHz, acetone-d$_6$) δ 8.85 (s, 1H), 7.87 (t, *J* = 5.9 Hz, 1H), 7.47 (d, *J* = 8.0 Hz, 2H), 7.41 (d, *J* = 7.9 Hz, 2H), 7.28 (d, *J* = 8.9 Hz, 1H), 4.66-4.62 (m, 3H), 4.59 – 4.53 (m, 2H), 4.37-4.33 (m, 1H), 3.93 (d, *J* = 10.7 Hz, 1H), 3.76 (dd, *J* = 10.8, 3.9 Hz, 1H), 2.47 (s, 3H), 2.38 – 2.28 (m, 5H), 2.18– 2.16 (m, 2H), 1.91 – 1.84 (m, 2H), 1.00 (s, 9H); ^{13}C NMR (126 MHz, acetone-d$_6$) δ 174.1, 172.1, 171.7, 170.8, 150.4, 148.2, 139.6, 131.4, 130.4, 128.9, 127.8, 69.8, 59.1, 57.1, 56.6, 42.2, 37.5, 35.2, 34.2, 32.8, 26.0, 20.9, 15.4.

Synthesis of *N*1-((*S*)-1-((*2S,4R)*-4-hydroxy-2-((4-(4-methylthiazol-5-yl)benzyl)carbamoyl)pyrrolidin-1-yl)-3,3-dimethyl-1-oxobutan-2-yl)-*N*5-(3-((6-hydroxy-5-(1-methyl-3-(trifluoromethyl)-1*H*-pyrazol-5-yl)-3',5'-bis(trifluoromethyl)-[1,1'-biphenyl]-2-yl)oxy)propyl)glutaramide, CSI107: To a stirred solution of compound **1** (13 mg, 0.020 mmol) in dry DMF (700 µL) and under inert atmosphere, DIPEA (19.2 µL, 0.110 mmol) was added, followed by the addition of HATU (8.38 mg, 0.022 mmol) and finally of compound **9** (10 mg, 0.0184 mmol). The reaction stirred at room temperature overnight. The volatiles were removed under reduced pressure and the crude residue was purified via silica gel chromatography (2-10% MeOH in EtOAc) to give the desired product as an off-white powder (9 mg, 47%). **CSI107:** ^{1}H NMR (500 MHz, acetone-*d$_6$*) δ 8.83 (s, 1H), 8.31 (broad s, 1H), 8.12 (s, 2H), 8.01 (s, 1H), 7.98 (s, 1H), 7.91 (s, 2H), 7.45 (d, *J* = 8.1 Hz, 2H), 7.40 (d, *J* = 8.1 Hz, 2H), 7.33 (d, *J* = 8.6 Hz, 1H), 6.85 (d, *J* = 8.6 Hz, 1H), 6.63 (s, 1H), 4.66 (t, *J* = 8.0 Hz, 1H), 4.62 (d, *J* = 8.8 Hz, 1H), 4.53 (dd, *J* = 15.1, 6.7 Hz, 2H), 4.36 (dd, *J* = 15.4, 5.6 Hz, 1H), 4.09 (t, *J* = 6.1 Hz, 2H), 4.00 (d, *J* = 10.9 Hz, 1H), 3.83 (s, 3H), 3.72 (dd, *J* = 10.9, 3.9 Hz, 1H), 3.28 – 3.20 (m, 2H), 2.92 (s, 4H), 2.46 (s, 3H), 2.28 (t, *J* = 7.1 Hz, 2H), 2.17 – 2.14 (m, 2H), 1.88 – 1.80 (m, 4H), 0.99 (s, 9H); ^{13}C NMR (126 MHz, acetone-*d$_6$*) δ 173.5, 173.4, 172.5, 171.8, 158.9, 153.7, 151.2, 149.1, 142.3, 141.1, 141.1, 140.5, 137.5, 133.3, 133.1, 132.3, 131.7, 131.4, 131.3, 129.9, 128.7, 125.7, 123.6, 121.7, 121.5, 111.6,

106.2, 105.4, 70.7, 66.7, 59.9, 58.3, 57.4, 55.5, 43.1, 38.3, 38.2, 35.8, 35.7, 35.3, 31.9, 26.9, 23.3, 22.8, 16.3; ESI-HRMS m/z for $C_{49}H_{53}F_9N_7O_7S$ $[M+H]^+$, calculated 1054.3578; found 1054.3564.

➢ Synthesis of CSI95

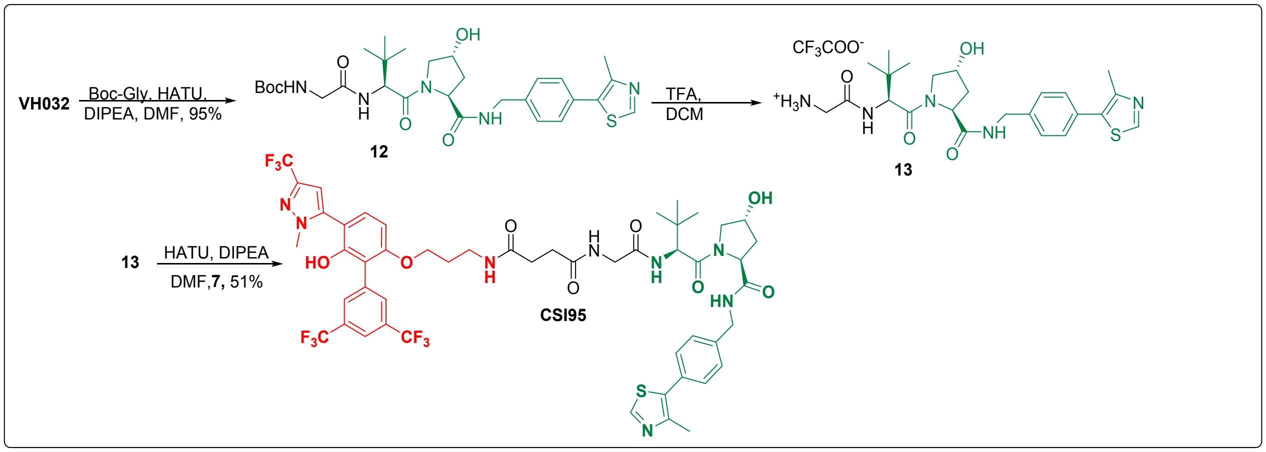

Scheme S7

Synthesis of *tert*-butyl (2-(((S)-1-((*2S,4R*)-4-hydroxy-2-((4-(4-methylthiazol-5-yl)benzyl) carbamoyl)pyrrolidin-1-yl)-3,3-dimethyl-1-oxobutan-2-yl)amino)-2-oxoethyl)carbamate, 12: In a flask containing compound **VH032** (30 mg, 0.055 mmol) as a solution in dry DMF (700 µL) and under inert atmosphere, DIPEA (8.72 µL, 0.0501 mmol) and HATU (19 mg, 0.05 mmol) were added followed by the addition of Boc-glycine (8.8 mg, 0.05 mmol). The reaction stirred overnight at room temperature. Solvent was removed and the crude residue was purified via silica gel chromatography (2%-5% MeOH in EA) to give the desired product as a white solid (29.4 mg, yield 95%). **12:** ^{1}H NMR (500 MHz, acetone-d_6) δ 8.85 (s, 1H), 7.98 (broad s, 1H), 7.47 (d, *J* = 8.1 Hz, 2H), 7.40 (d, *J* = 8.2 Hz, 2H), 6.55 (broad s, 1H), 4.71-4.64 (m, 2H), 4.60 – 4.51 (m, 2H), 4.37 (dd, *J* = 15.5, 5.4 Hz, 1H), 3.93 (d, *J* = 10.6 Hz, 1H), 3.85 – 3.73 (m, 4H), 2.47 (s, 3H), 2.18 (dd, *J* = 7.7, 2.7 Hz, 2H), 1.42 (s, 9H), 0.99 (s, 9H); ^{13}C NMR (126 MHz, acetone-d_6) δ 172.6, 171.2, 170.7, 157.2, 151.3, 149.1, 140.3, 132.3, 131.3, 129.8, 128.7, 79.9, 79.2, 70.8, 60.1, 57.9, 57.5, 44.7, 43.2, 38.4, 28.6, 26.8, 16.3.

Synthesis of 2-(((*S*)-1-((*2S,4R*)-4-hydroxy-2-((4-(4-methylthiazol-5-yl)benzyl)carbamoyl)pyrrolidin-1-yl)-3,3-dimethyl-1-oxobutan-2-yl)amino)-2-oxoethan-1-aminium 2,2,2-trifluoroacetate, 13: In a flask containing compound **12** (28 mg, 0.048 mmol), DCM (400 µL) was added followed by the addition of TFA (40 µL). The resulting solution was stirred at room temperature for 4 hours and upon completion of the reaction (confirmed via TLC), the volatiles were removed, and the resulting residue was used onto the next step without any further purification.

Synthesis of *N*1-(2-(((*S*)-1-((*2S,4R*)-4-hydroxy-2-((4-(4-methylthiazol-5-yl)benzyl)carbamoyl)pyrrolidin-1-yl)-3,3-dimethyl-1-oxobutan-2-yl)amino)-2-oxoethyl)-*N*4-(3-((6-hydroxy-5-(1-methyl-3-(trifluoro methyl)-1*H*-pyrazol-5-yl)-3',5'-bis(trifluoromethyl)-[1,1'-biphenyl]-2-yl)oxy)propyl) succinimide, CSI95: To a stirred solution of compound **13** (31.6 mg, 0.053 mmol), in dry DMF (700 µL), DIPEA (50 µL, 0.287 mmol) was added, followed by the addition of HATU (21.8 mg, 0.0574 mmol) and compound **7** (30 mg,

0.0478 mmol). The reaction was stirred overnight at room temperature. Volatiles were removed and the crude residue was purified with silica gel chromatography (2-10% MeOH in EtOAc) to give the desired product as a white powder (26.5 mg, yield=51%). **CSI95:** ^{1}H NMR (500 MHz, acetone-d_6) δ 8.82 (s, 1H), 8.10 (s, 2H), 8.01 (s, 1H), 7.97 (s, 1H), 7.45 (d, J = 8.1 Hz, 2H), 7.40 (d, J = 8.1 Hz, 2H), 7.33 (d, J = 8.6 Hz, 1H), 6.85 (d, J = 8.6 Hz, 1H), 6.64 (s, 1H), 4.69 (t, J = 8.2 Hz, 2H), 4.63 – 4.58 (m, 2H), 4.52 (s,2H), 4.37 (dd, J = 15.4, 5.4 Hz, 2H), 4.08 (t, J = 6.3 Hz, 2H), 3.83 (s, 3H), 3.70 (dd, J = 11.0, 3.8 Hz, 2H), 3.24 (m, 3H) 2.93 (s, 8H), 2.50 (s, 3H), 2.46 (s, 2H), 0.99 (s, 9H); ^{13}C NMR (126 MHz, acetone-d_6) δ 174.1, 173.4, 172.5, 171.5, 171.1, 161.6, 158.9, 153.7, 151.2, 151.2, 149.1, 142.4, 141.3, 140.5, 137.6, 133.3, 133.0, 133.0, 131.8, 131.3, 129.9, 128.7, 125.8, 123.9, 123.6, 122.9, 121.5, 119.3, 116.9, 111.6, 106.2, 105.5, 70.8, 66.8, 60.1, 58.7, 57.5, 43.4, 43.1, 38.4, 38.2, 36.4, 36.1, 31.5, 31.4, 26.9, 19.2, 16.3; ESI-HRMS m/z for $C_{50}H_{54}F_9N_8O_8S$ $[M+H]^+$, calculated 1097.3636; found 1097.3640.

➢ Synthesis of CSI135

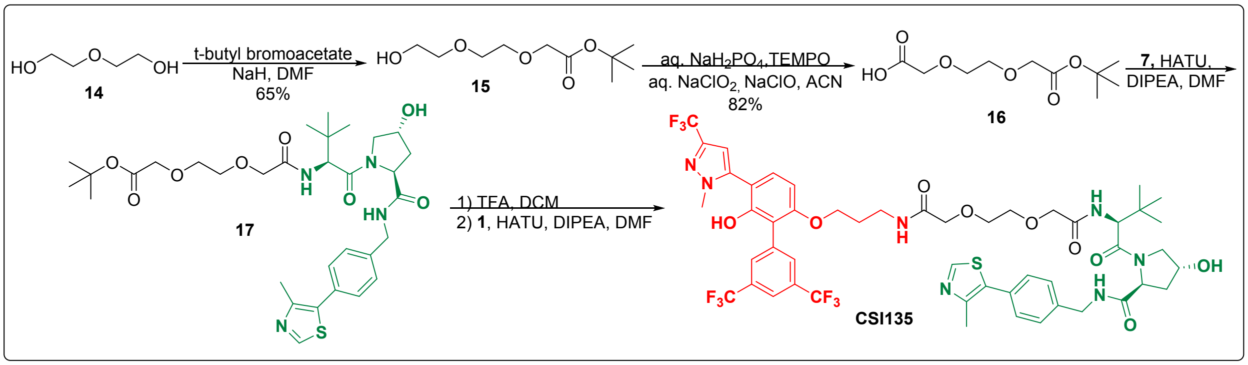

Scheme S8

Synthesis of *tert*-butyl 2-(2-(2-hydroxyethoxy)ethoxy)acetate, 15: To a stirred solution of diethylene glycol (500 mg, 4.71 mmol) in dry DMF (1.45 mL) at 0° C, 60% NaH (45.3 mg, 1.89 mmol) was added under inert atmosphere. The mixture was allowed to warm up to room temperature and stirred for 1 hour. After 1h, the mixture was cooled to 0° C and *t*-butyl bromoacetate (306 mg, 0.57 mmol) was added dropwise and the reaction stirred at room temperature for 2 hours. Upon completion of reaction, water was slowly added, and the mixture was extracted with CH_2Cl_2. The combined organic layers were dried over anhydrous Na_2SO_4 and the volatiles were removed under reduced pressure. The crude mixture was purified via silica gel chromatography (eluent: 1-10% MeOH in DCM) to give the desired product as a yellow oil (yield =65%). The spectral data were in accordance with those reported in the literature.[4] **15:**^{1}H NMR (500 MHz, $CDCl_3$) δ 4.01 (t, J = 4.7 Hz, 2H), 3.76 – 3.68 (m, 6H), 3.64 – 3.59 (m, 2H), 1.47 (s, 9H); ^{13}C NMR (126 MHz, $CDCl_3$) δ 169.7, 81.9, 72.7, 71.0, 70.5, 69.1, 61.9, 28.3.

Synthesis of 2-(2-(2-(*tert*-butoxy)-2-oxoethoxy)ethoxy)acetic acid, 16: In a round bottom flask containing tert-butyl 2-(2-(2-hydroxyethoxy)ethoxy)acetate **15** (91.4 mg, 0.42 mmol) as a solution in ACN (2.3 mL), buffer solution of NaH_2PO_4 (pH 6.5, 1.9 mL) was added, followed by the addition of TEMPO (28.2 mg, 0.18 mmol), aq. $NaClO_2$ (301.6 mg in 3.8 mL of water) and NaClO (0.16 mL). The reaction mixture was stirred at 50° C for 5 hours. Saturated Na_2SO_3 was added until solution discoloration is observed and then ACN is removed under reduced pressure. The mixture was acidified with 2M HCl to pH 2 and extracted with

EtOAc. The combined organic layers were collected, dried over anhydrous Na_2SO_4, filtered and the volatiles were removed under reduced pressure to give the desired product a yellow oil (yield 82%). **16:** ^{1}H NMR (500 MHz, $CDCl_3$) δ 4.19 (s, 2H), 4.03 (s, 2H), 3.79 (dd, *J* = 6.0, 2.8 Hz, 2H), 3.75 (dd, *J* = 6.0, 2.7 Hz, 2H), 1.47 (s, 9H).

Synthesis of *tert*-butyl 2-(2-(2-(((*S*)-1-((2S,4R)-4-hydroxy-2-((4-(4-methylthiazol-5-yl)benzyl)carbamoyl)pyrrolidin-1-yl)-3,3-dimethyl-1-oxobutan-2-yl)amino)-2-oxoethoxy)ethoxy) acetate, 17: To a stirred solution of 2-(((*S*)-1-((2*S*,4*R*)-4-hydroxy-2-((4-(4-methylthiazol-5-yl)benzyl) carbamoyl)pyrrolidin-1-yl)-3,3-dimethyl-1-oxobutan-2-yl)amino)-2-oxoethan-1-aminium2,2,2-trifluoro acetate (128 mg, 0.235 mmol) as a solution in dry DMF (0.7 mL), DIPEA (186 uL, 1.07 mmol) and HATU (122 mg, 0.32 mmol) were added followed by the addition of 2-(2-(2-(*tert*-butoxy)-2-oxoethoxy)ethoxy)acetic acid **14** (50 mg, 0.213 mmol) and the reaction stirred at room temperature overnight. The reaction was diluted in ethyl acetate, washed with 5% aq. solution of citric acid and saturated $NaHCO_3$ and the organic layer was concentrated under reduced pressure. The crude product was purified with silica gel chromatography (5% MeOH in EtOAc to 15% MeOH in EtOAc) to afford the desired product as a yellow-white solid (45 mg, yield= 43%). **17:** ^{1}H NMR (500 MHz, $CDCl_3$) δ 8.67 (s, 1H), 7.53 (s, 1H), 7.42 (d, *J* = 8.4 Hz, 1H), 7.34 (q, *J* = 8.3 Hz, 4H), 4.73 (t, *J* = 8.0 Hz, 1H), 4.52 (dd, *J* = 14.8, 6.3 Hz, 3H), 4.46 (d, *J* = 8.3 Hz, 1H), 4.34 (dd, *J* = 15.0, 5.4 Hz, 2H), 4.13 – 4.06 (m, 2H), 4.04 (s, 1H), 4.02 – 3.99 (m, 3H), 3.97 (s, 1H), 3.71 (s, 3H), 3.59 (dd, *J* = 11.3, 3.3 Hz, 2H), 2.51 (s, 3H), 2.13 (dd, *J* = 13.5, 8.1 Hz, 2H), 1.45 (s, 9H), 0.95 (s, 9H); ^{13}C NMR (126 MHz, $CDCl_3$) δ 171.5, 170.9, 170.9, 169.8, 150.4, 148.6, 138.3, 131.8, 130.9, 129.6, 128.2, 82.3, 71.7, 71.0, 70.9, 70.9, 70.4, 70.3, 70.2, 68.9, 58.6, 57.7, 56.9, 43.3, 36.1, 34.9, 29.8, 28.2, 26.5, 16.2; ESI-MS m/z for $C_{32}H_{47}N_4O_8S^+$ $[M+H]^+$ calculated 647.31; found 646.95.

Synthesis of (2*S*,4*R*)-1-((S)-2-(tert-butyl)-15-((6-hydroxy-5-(1-methyl-3-(trifluoromethyl)-1*H*-pyrazol-5-yl)-3',5'-bis(trifluoromethyl)-[1,1'-biphenyl]-2-yl)oxy)-4,11-dioxo-6,9-dioxa-3,12-diazapentadecanoyl)-4-hydroxy-*N*-(4-(4-methylthiazol-5-yl)benzyl)pyrrolidine-2-carboxamide, CSI135: To a solution of **17** (17 mg, 0.027 mmol) in CH_2Cl_2 (0.4 mL), TFA (40 µL) was added and the reaction mixture stirred for 3 hours at room temperature. Upon completion of the reaction, monitored by TLC, the volatiles were removed. DMF (0.8 mL) was added, followed by the addition of DIPEA (45.7 µL, 0.262 mmol) and HATU (15.0 mg, 0.0394 mmol) under inert atmosphere. Finally, **1** (20.2 mg, 0.0315 mmol) was added and the reaction stirred at room temperature overnight. The reaction mixture was diluted in EtOAc and was sequentially with 5% citric acid, sat. $NaHCO_3$ and brine. The organic layer was dried over Na_2SO_4 and the volatiles were removed under reduced pressure. The crude product was purified via silica gel chromatography (3% MeOH in EtOAc to 15%) to give the desired product as a white solid (8 mg, yield 30%). **CSI135**: ^{1}H NMR (500 MHz, acetone-*d$_6$*) δ 8.83 (s, 1H), 8.16 (s, 1H), 7.98 (s, 1H), 7.84 (t, *J* = 6.5 Hz, 1H), 7.52 – 7.48 (m, 1H), 7.43 (q, *J* = 8.7 Hz, 4H), 7.32 (d, *J* = 8.6 Hz, 1H), 6.83 (d, *J* = 8.6 Hz, 1H), 6.65 (s, 1H), 4.71 (d, *J* = 9.5 Hz, 1H), 4.65 (t, *J* = 8.3 Hz, 1H), 4.57 – 4.47 (m, 3H), 4.39 (dd, *J* = 15.0, 5.3 Hz, 2H), 4.08 (t, *J* = 6.1 Hz, 2H), 4.00 (s, 3H), 3.97 (s, 1H), 3.91 (s, 1H), 3.83 (s, 3H), 3.80 (dd, *J* = 13.5, 6.6 Hz, 4H), 3.72 (d, *J* = 9.4 Hz, 4H), 2.85 (s, 4H), 2.46 (s, 3H), 1.85 – 1.81 (m, 2H), 1.01 (s, 9H); ^{13}C NMR (126 MHz, acetone-*d$_6$*) δ 173.4, 172.4, 171.2, 170.1, 169.7, 158.9, 153.6, 151.2, 149.1, 142.3, 140.3, 137.5, 133.5, 133.1, 132.2, 131.7, 131.4, 131.4, 129.9, 128.6, 121.5, 116.8, 111.5, 110.9, 106.2, 105.5, 71.7, 71.3, 71.3, 70.9, 70.6, 66.8, 60.2, 57.8, 57.1, 43.1, 38.7, 38.2, 36.9, 35.5, 32.6, 32.3, 26.8, 23.3, 16.3, 14.4; ESI-HRMS m/z for $C_{50}H_{55}F_9N_7O_9S^+$ $[M+H]^+$ calculated 1100.3633; found 1100.3625.

➢ *Synthesis of CSI212*

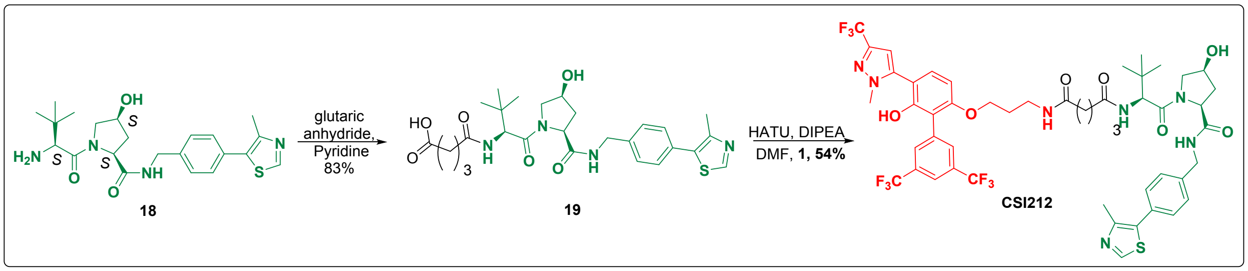

Scheme S9

Synthesis of 5-(((*S*)-1-((2*S*,4*S*)-4-hydroxy-2-((4-(4-methylthiazol-5-yl)benzyl)carbamoyl)pyrrolidin-1-yl)-3,3-dimethyl-1-oxobutan-2-yl)amino)-5-oxopentanoic acid, 19: In a flask containing **18** (23.1 mg, 0.042 mmol) as a solution in dry pyridine (0.4 mL), glutaric anhydride (10 mg, 0.084 mmol) was added and the reaction stirred overnight at room temperature. Upon completion of the reaction monitored via TLC, solvent was removed under reduced pressure and the crude residue was purified via silica gel chromatography (eluents 5-15% MeOH in EtOAc) to give the desired product as an off-white solid (19 mg, yield 83%). **19:** ^{1}H NMR (500 MHz, acetone-d_6) δ 8.86 (s, 1H), 8.41 (s, 1H), 8.02 (s, 0H), 7.46 (d, *J* = 8.1 Hz, 4H), 7.41 (d, *J* = 8.0 Hz, 3H), 7.36 (d, *J* = 8.8 Hz, 2H), 4.65 (q, *J* = 9.9, 8.1 Hz, 7H), 4.60 (d, *J* = 6.8 Hz, 1H), 4.39 – 4.34 (m, 5H), 3.92 (dd, *J* = 10.7, 4.2 Hz, 3H), 3.82 (d, *J* = 10.7 Hz, 3H), 2.47 (s, 4H), 2.34 (dq, *J* = 20.6, 7.0 Hz, 20H), 1.88 – 1.83 (m, 7H), 1.00 (s, 16H); ^{13}C NMR (126 MHz, acetone-d_6**)** δ 175.6, 174.4, 173.2, 172.2, 151.39, 149.1, 139.8, 132.3, 131.4, 129.9, 128.6, 79.2, 71.6, 60.6, 58.6, 57.7, 43.4, 37.1, 35.75, 35.2, 33.8, 26.9, 21.8, 16.3; ESI-MS m/z for $C_{27}H_{37}N_4O_6S$ calculated 545.2434, found 545.05.

Synthesis of *N*[1]-((S)-1-((2S,4S)-4-hydroxy-2-((4-(4-methylthiazol-5-yl)benzyl)carbamoyl)pyrrolidin-1-yl)-3,3-dimethyl-1-oxobutan-2-yl)-*N*5-(3-((6-hydroxy-5-(1-methyl-3-(trifluoromethyl)-1H-pyrazol-5-yl)-3',5'-bis(trifluoromethyl)-[1,1'-biphenyl]-2-yl)oxy)propyl)glutaramide, CSI212: To a stirred solution of **1** (26 mg, 0.0404 mmol) in dry DMF (0.2 mL) and under inert atmosphere, dry DIPEA (35 uL, 0.22 mmol) was added and the solution was stirred at room temperature for 15 minutes. Then, HATU (17 mg, 0.044 mmol) was added, followed by the addition of **19** (20 mg, 0.0367 mmol) as a solution in dry DMF (0.5 mL) and the reaction stirred overnight at room temperature. Solvent was removed under reduced pressure and the crude residue was purified via HPLC (H_2O + formic acid 0.1% / ACN + formic acid 0.1% 90% to 5%, Rt: 32 mins) to give the desired product as an off-white solid (21 mg, yield 54%). **CSI212:** ^{1}H NMR (300 MHz, acetone-d_6) δ 8.84 (s, 1H), 8.14 (s, 1H), 7.99 (s, 1H), 7.47 (d, *J* = 8.1 Hz, 2H), 7.41 (d, *J* = 8.2 Hz, 2H), 7.34 (dd, *J* = 8.5, 3.0 Hz, 2H), 7.29 – 7.14 (m, 3H), 6.84 (d, *J* = 8.6 Hz, 1H), 6.64 (s, 1H), 4.71 – 4.61 (m, 2H), 4.57 – 4.52 (m, 1H), 4.39 – 4.31 (m, 2H), 4.09 (t, *J* = 5.8 Hz, 2H), 3.90 (dd, *J* = 10.5, 4.4 Hz, 1H), 3.83 (s, 3H), 3.23 (dq, *J* = 7.4, 5.0, 3.6 Hz, 3H), 2.93 (dd, *J* = 9.5, 1.1 Hz, 2H), 2.46 (s, 3H), 2.15 (dd, *J* = 11.3, 4.3 Hz, 4H), 1.84 – 1.78 (m, 4H), 1.00 (t, *J* = 1.4 Hz, 9H); ^{13}C NMR (75 MHz, acetone-d_6) δ 174.5, 173.0, 172.9, 172.2, 159.0, 151.3, 147.9, 147.1, 142.3, 139.9, 139.8, 133.3, 133.1, 133.1, 133.0, 131.8, 131.5, 131.4, 129.9, 128.7, 124.2, 124.1, 116.6, 114.7, 106.2, 105.4, 105.4, 71.7, 66.8, 60.6, 58.6, 58.0, 43.4, 38.2, 37.2, 36.1, 35.7, 35.5, 35.4, 32.6, 32.0, 26.9, 23.3, 22.9, 16.3, 14.3; HRMS m/z calculated for $C_{49}H_{53}F_9N_7O_7S$ $[M+H]^+$ calculated: 1054.3578, found: 1054.3542.

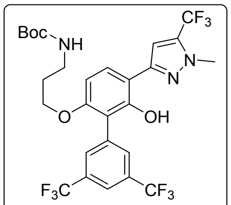
Boc
NH
CF3
N
N
O
OH
F3C
CF3
S6

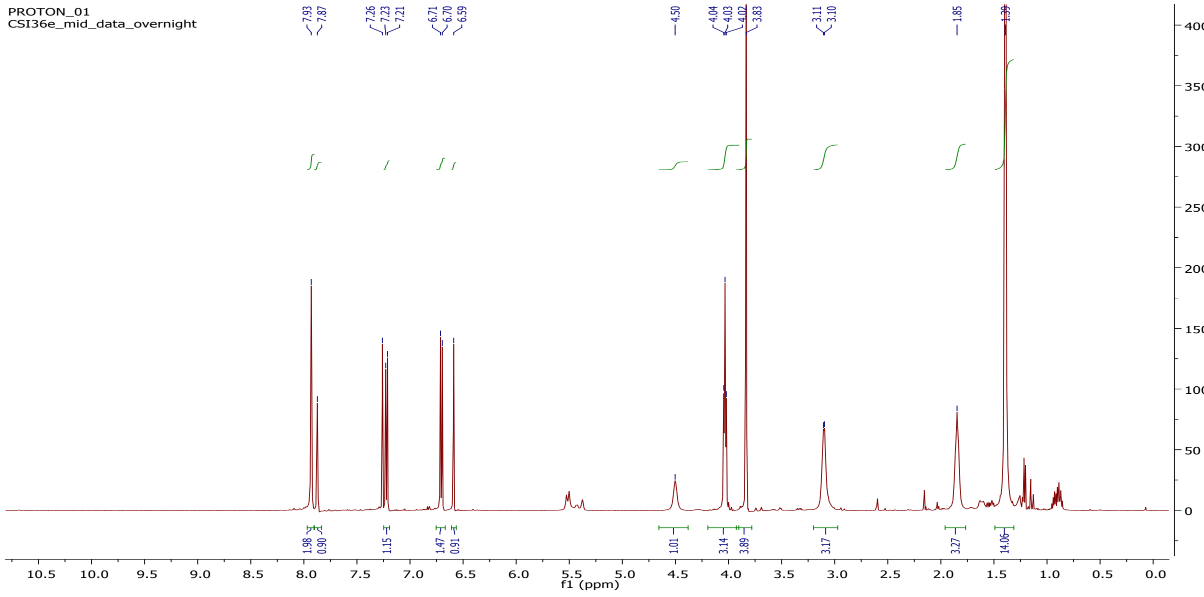
PROTON_01
CSI36e_mid_data_overnight
f1 (ppm)

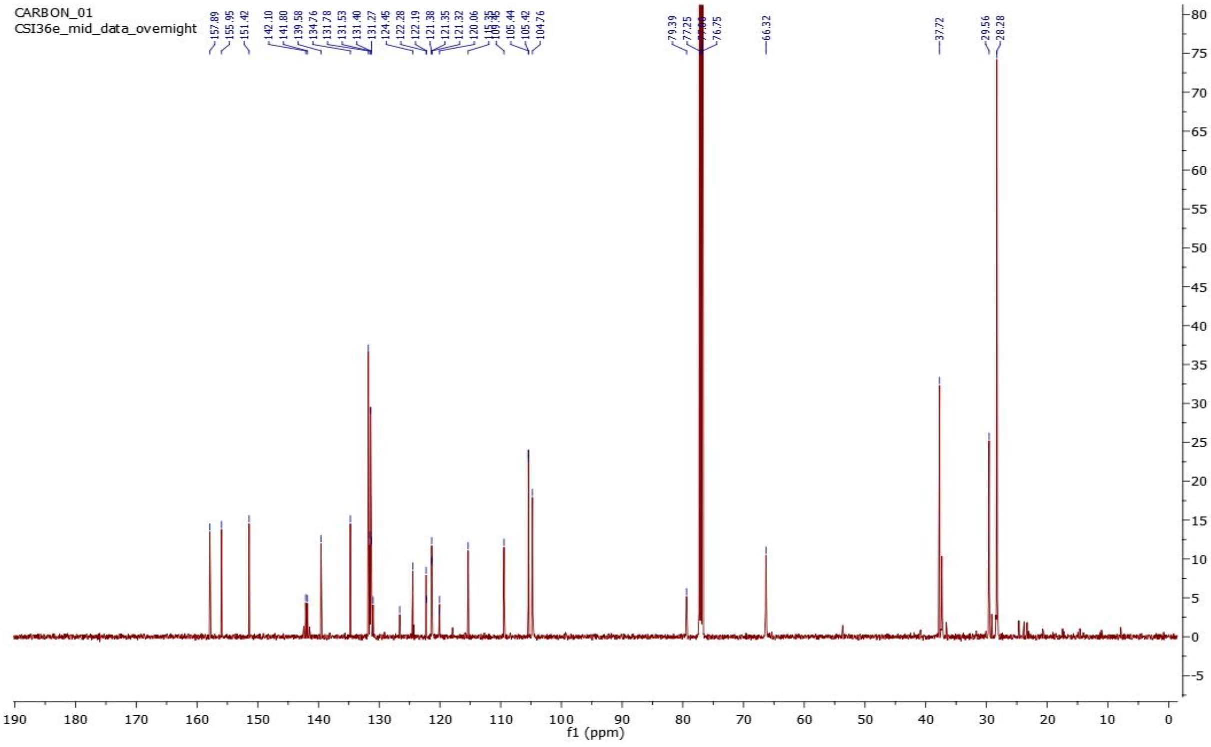
CARBON_01
CSI36e_mid_data_overnight
f1 (ppm)

Figure S1

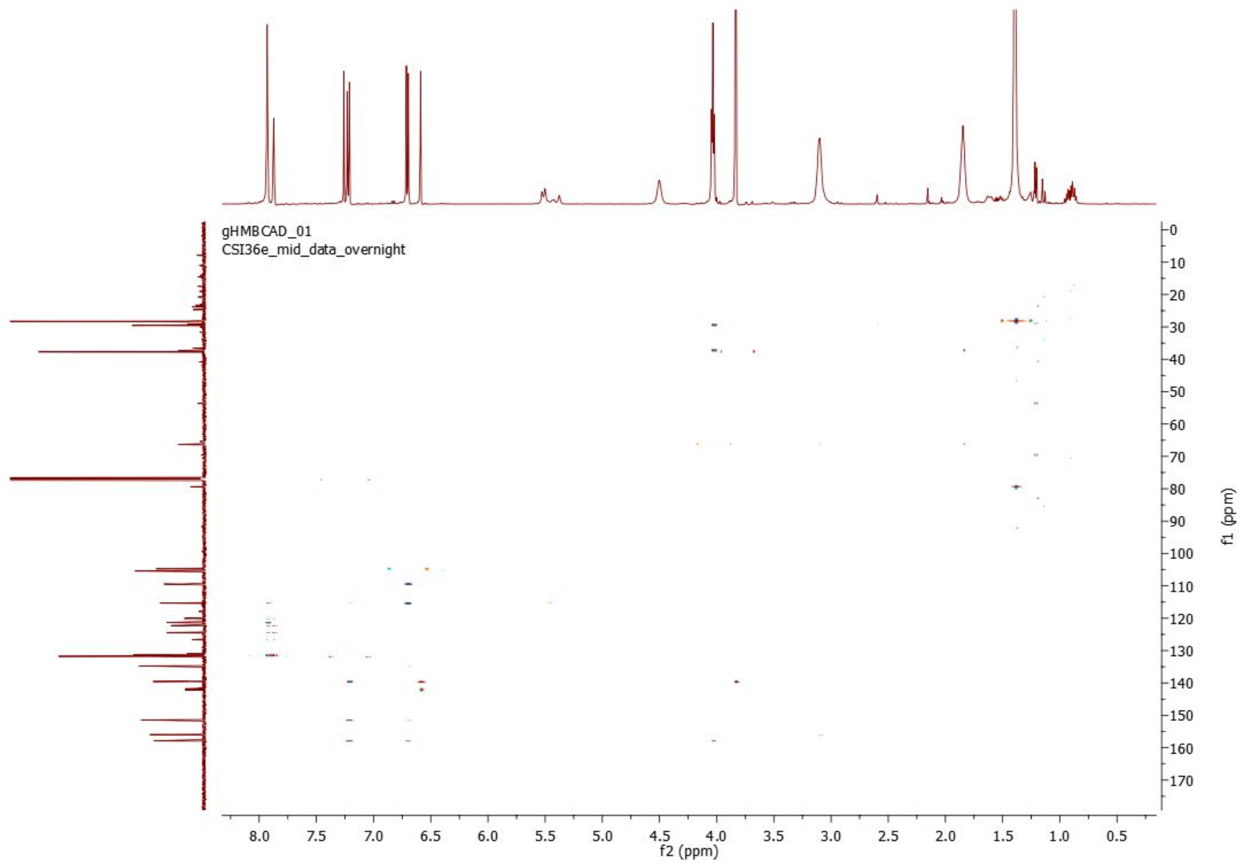

Figure S2

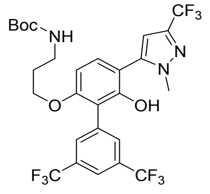
Boc
NH
CF3
N
N
O
OH
F3C
CF3

S7

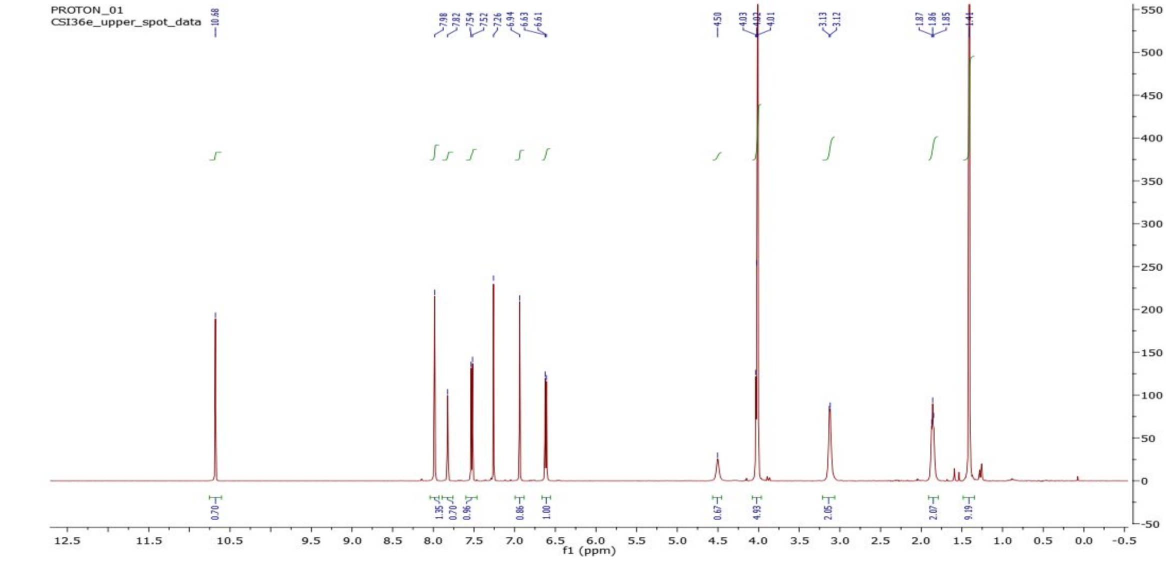
PROTON_01
CSI36e_upper_spot_data
f1 (ppm)

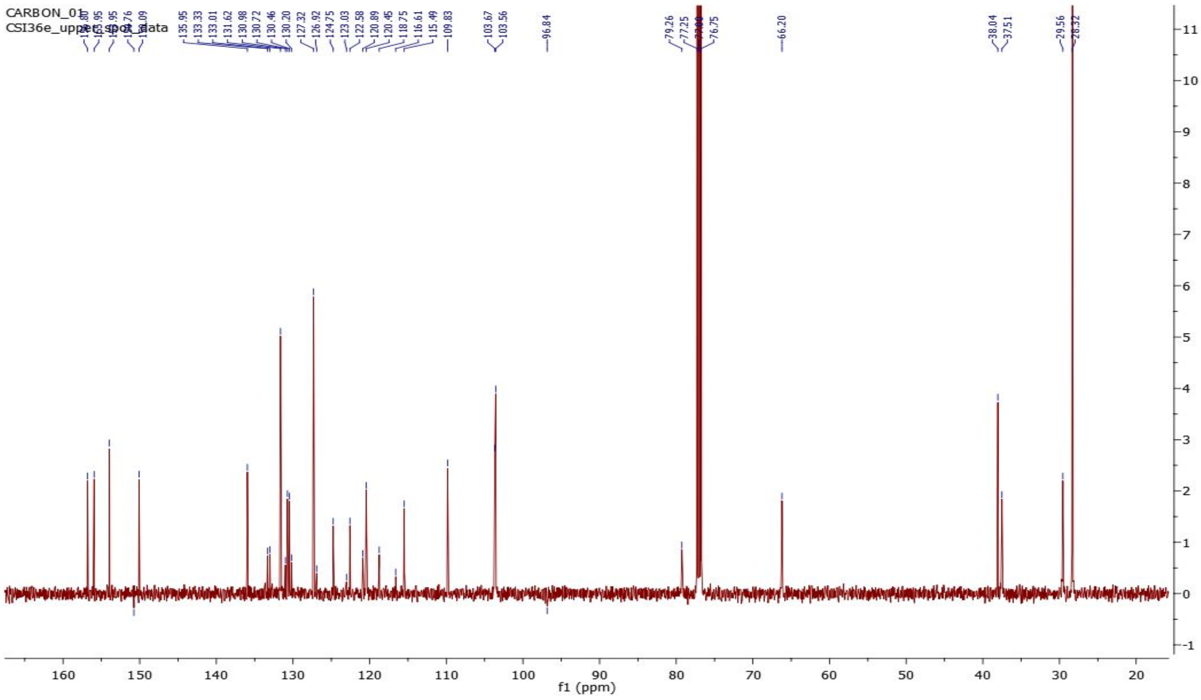
CARBON_01
CSI36e_upper_spot_data
f1 (ppm)

Figure S3

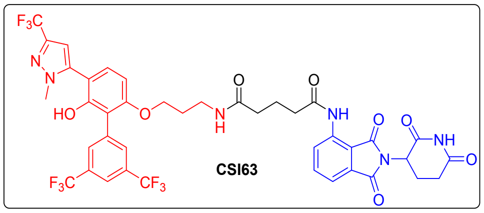

F3C
N
N
HO
O
N
H
O
O
NH
O
N
O
NH
O
O
F3C
CF3
CSI63

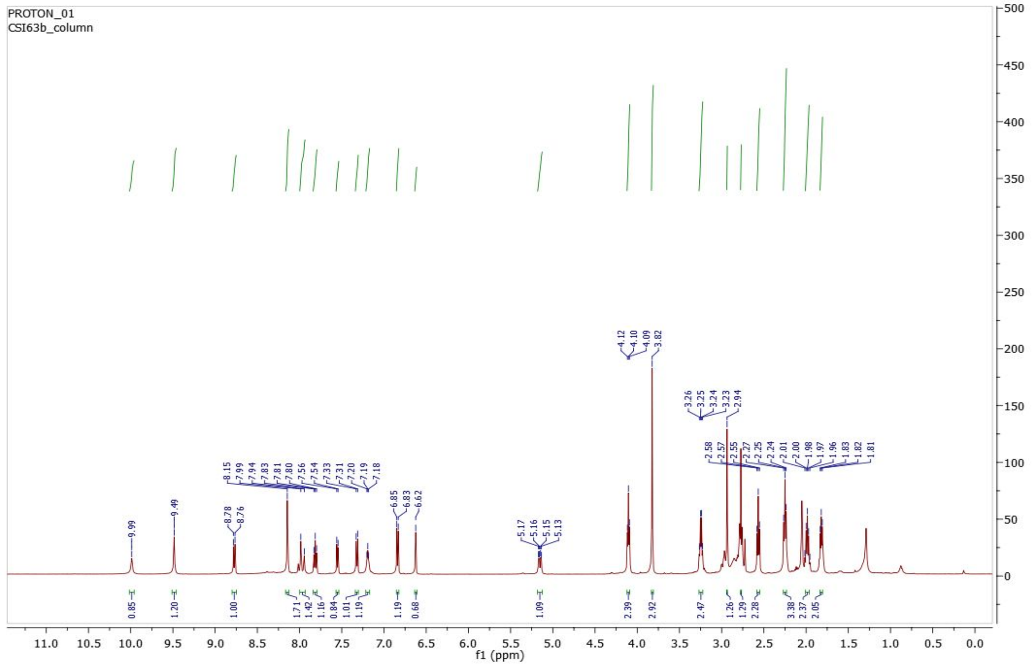

PROTON_01
CSI63b_column
f1 (ppm)

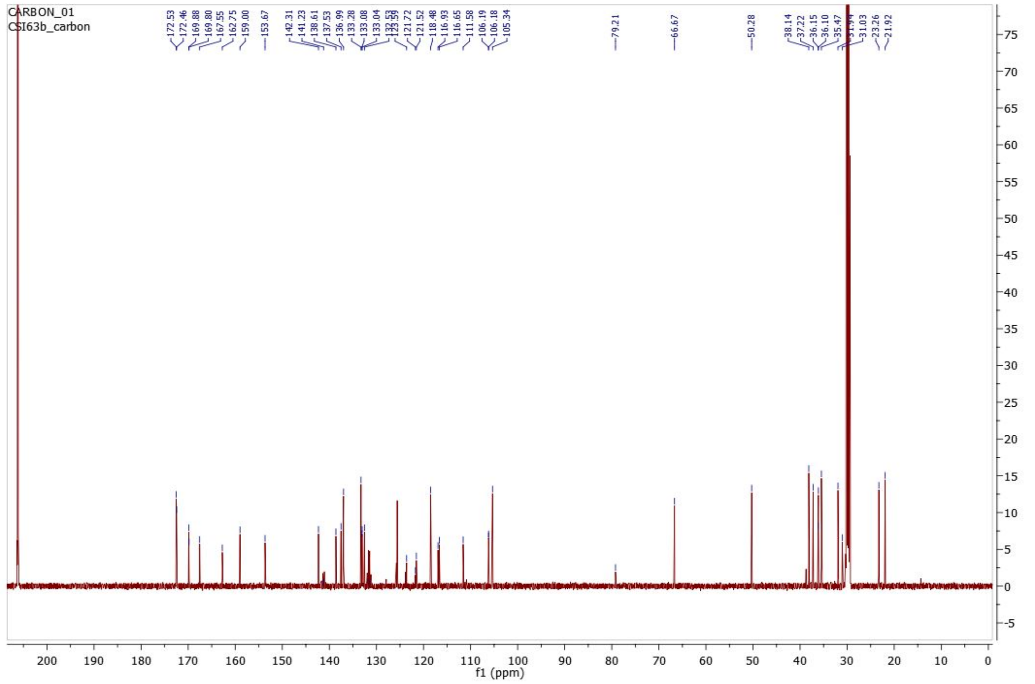

CARBON_01
CSI63b_carbon
f1 (ppm)

Figure S4

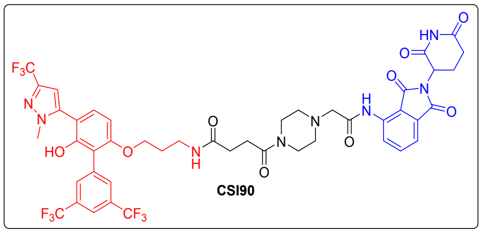

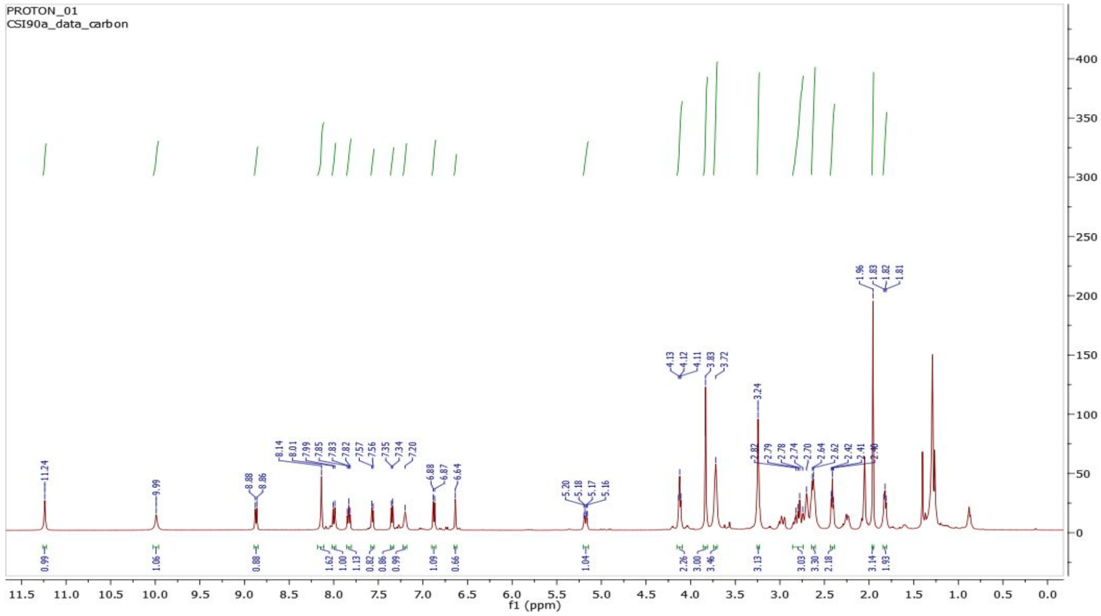

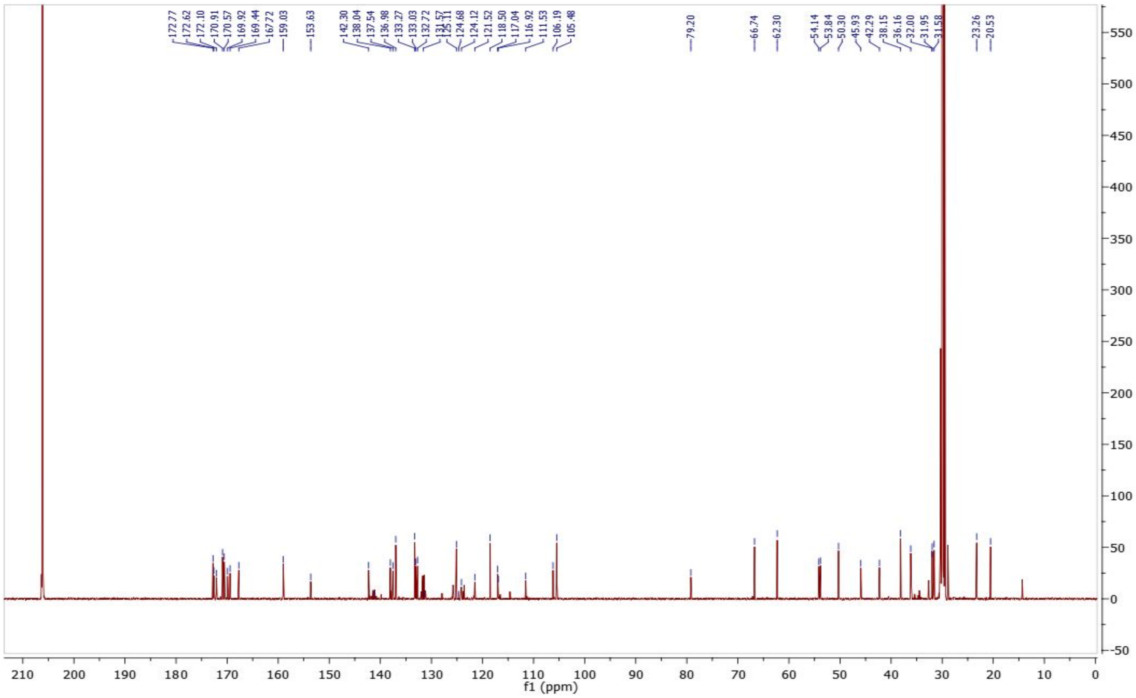

Figure S5

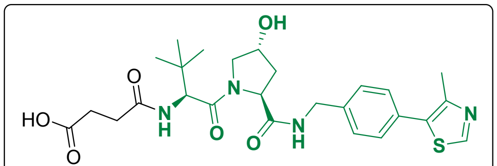

8

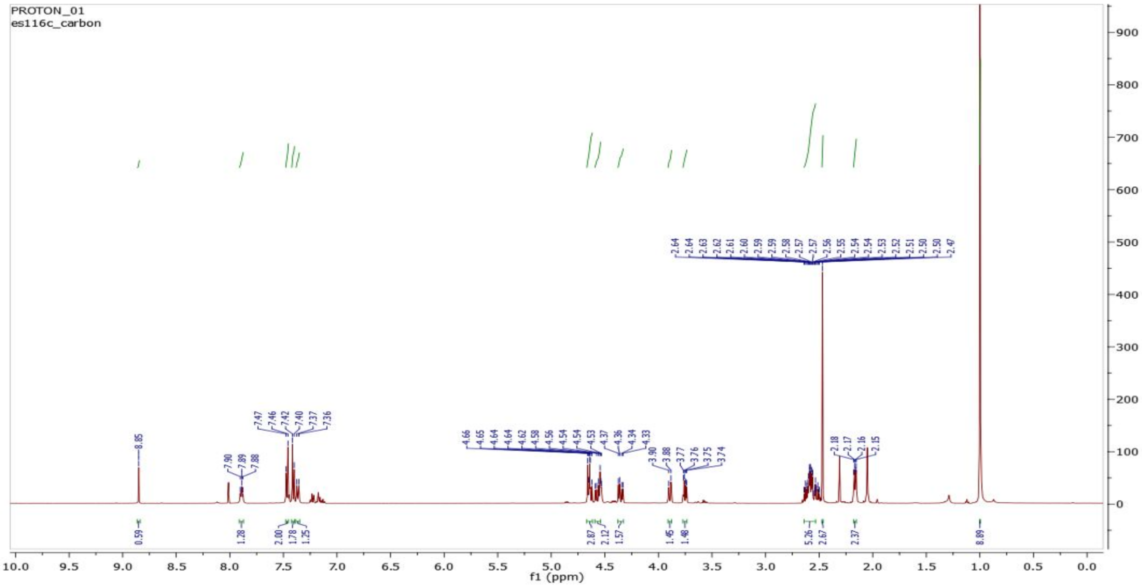

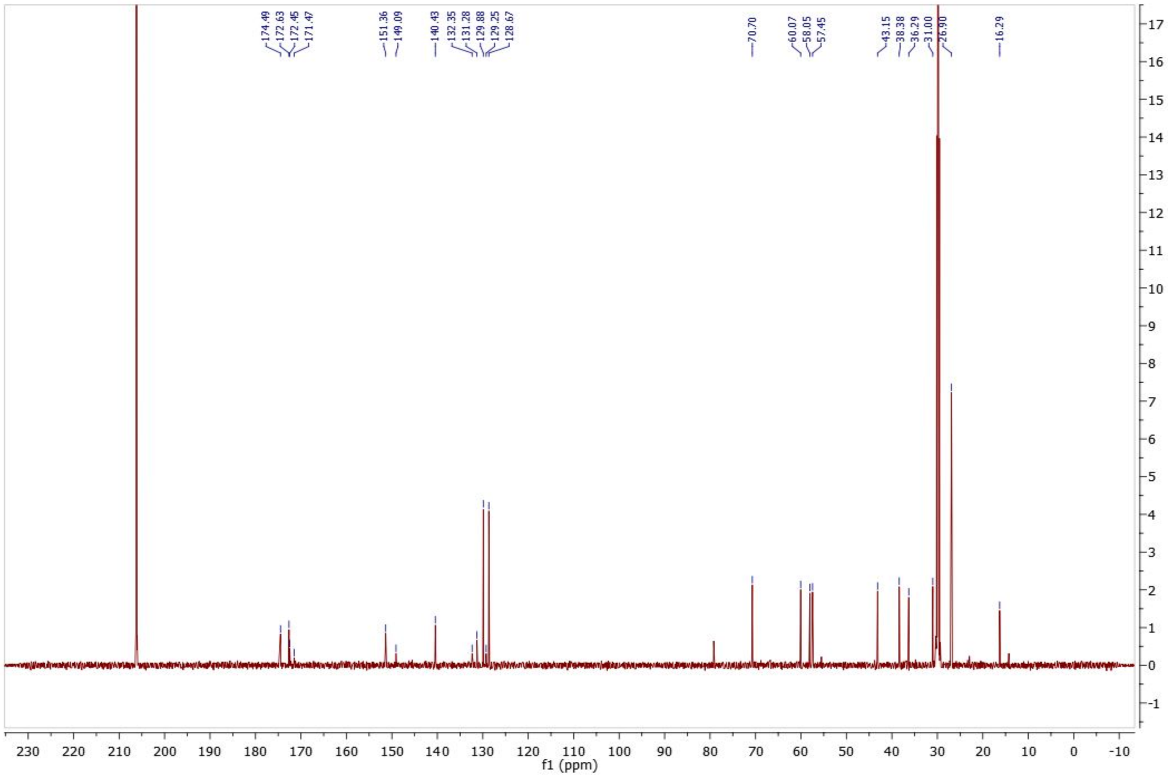

Figure S6

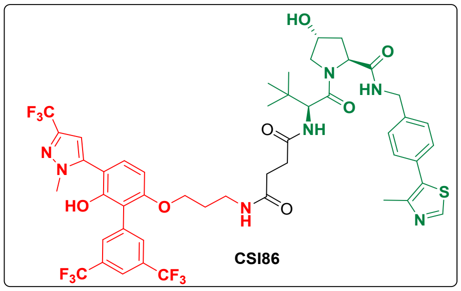
HO
O
N
HN
O
NH
O
F3C
N
N
HO
O
N
H
O
S
N
F3C
CF3
CSI86

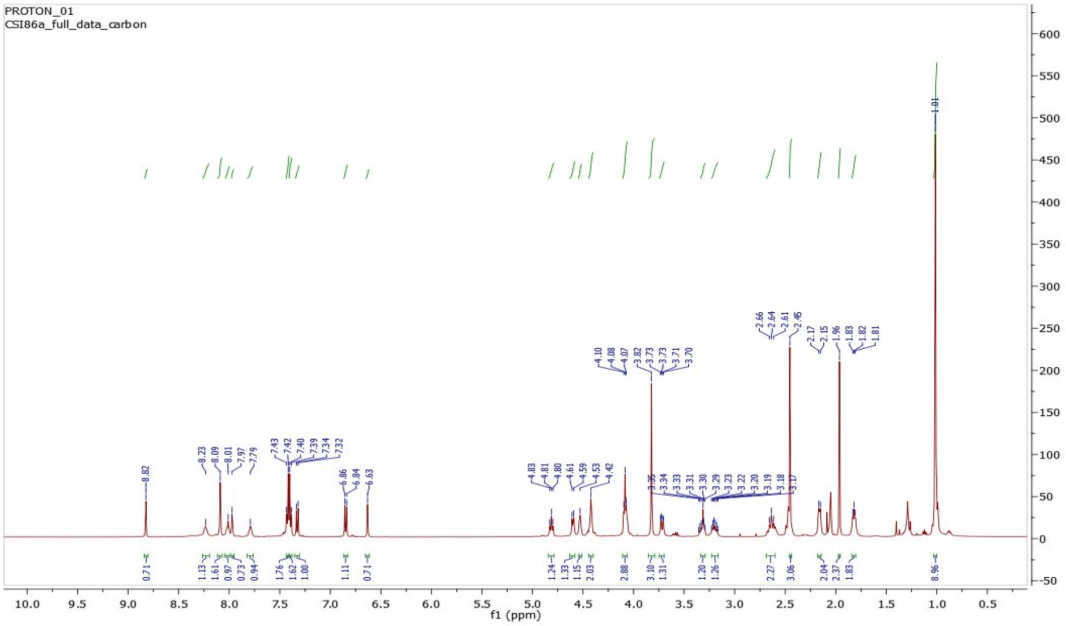
PROTON_01
CSI86a_full_data_carbon
f1 (ppm)

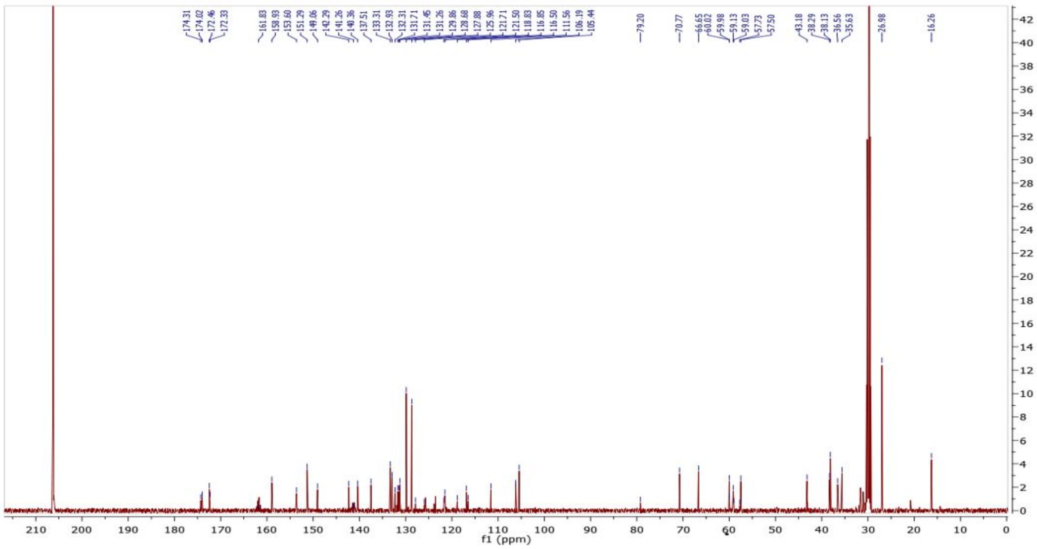
f1 (ppm)

Figure S7

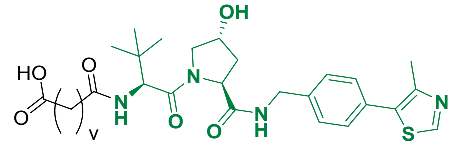

9, v=3

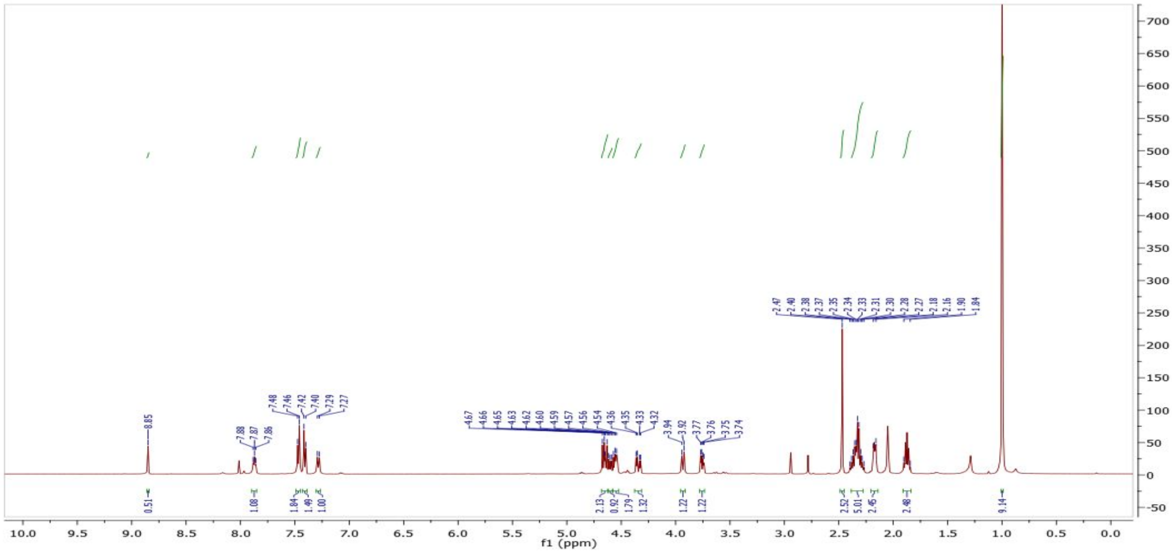

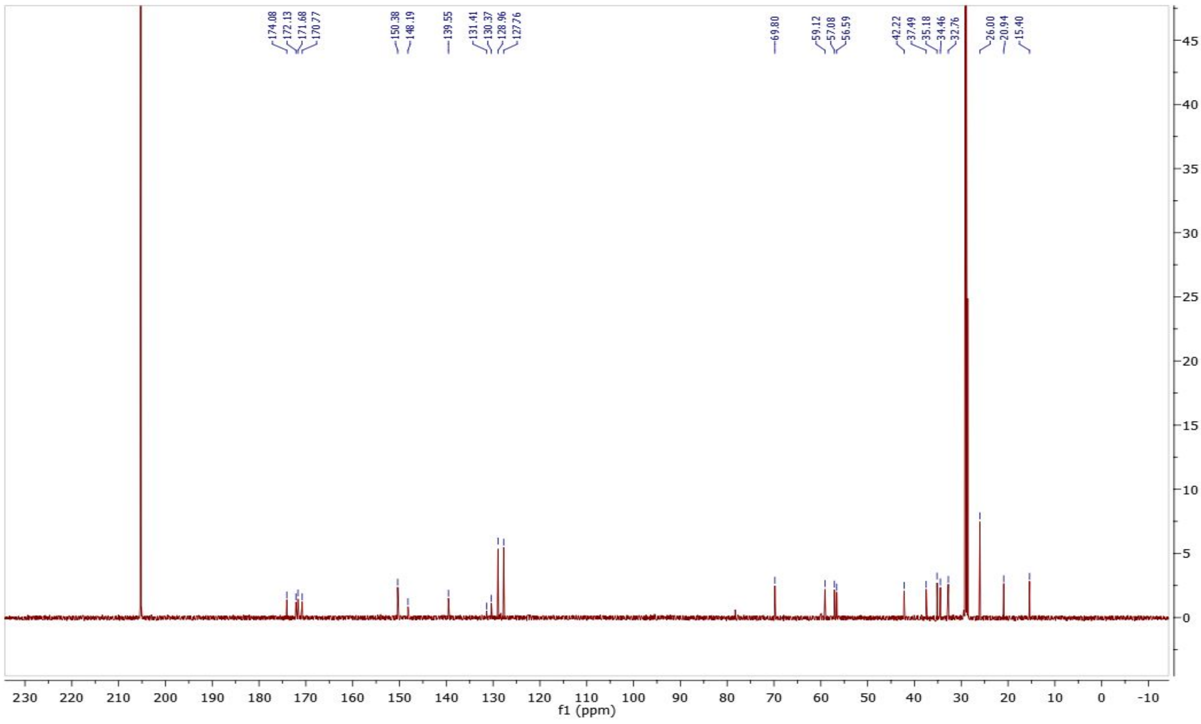

Figure S8

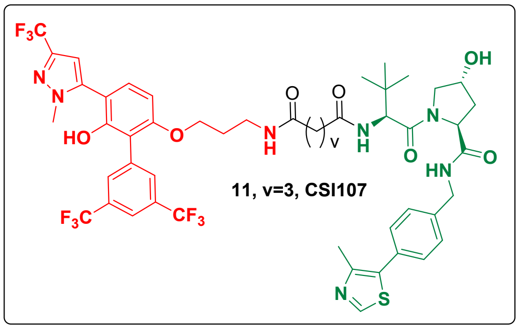
F3C
N
N
HO
O
N
H
O
O
( )v
N
H
O
N
OH
O
HN
N
S
F3C
CF3
11, v=3, CSI107

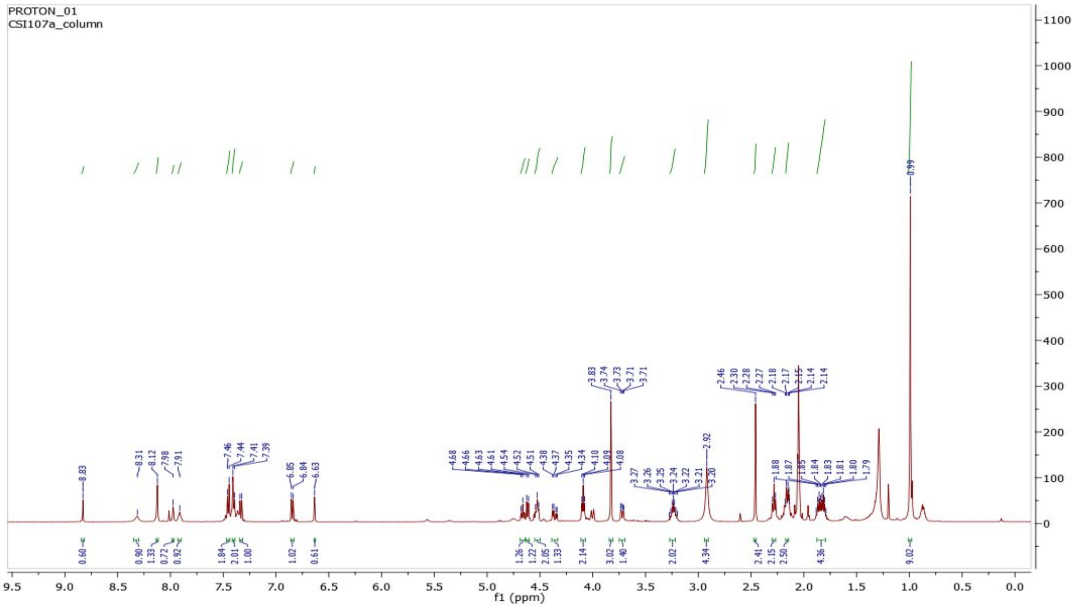
PROTON_01
CSI107a_column
f1 (ppm)

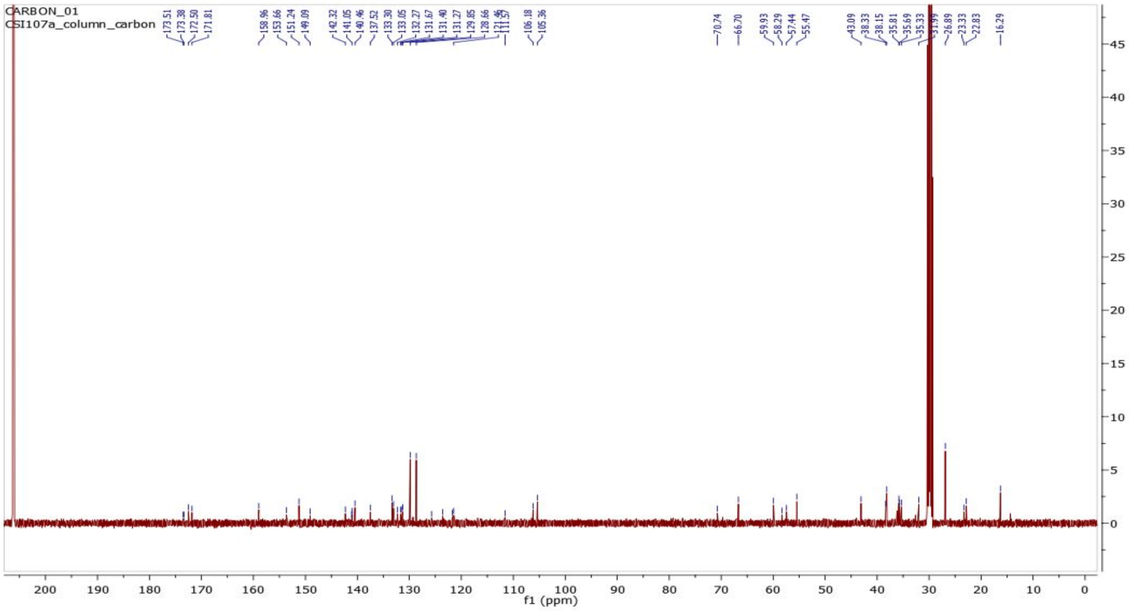
CARBON_01
CSI107a_column_carbon
f1 (ppm)

Figure S9

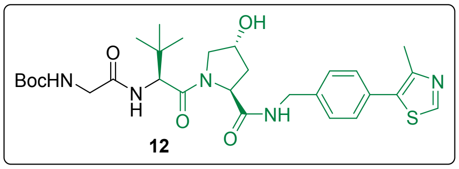
OH
BocHN
O
N
H
N
O
NH
O
N
S
12

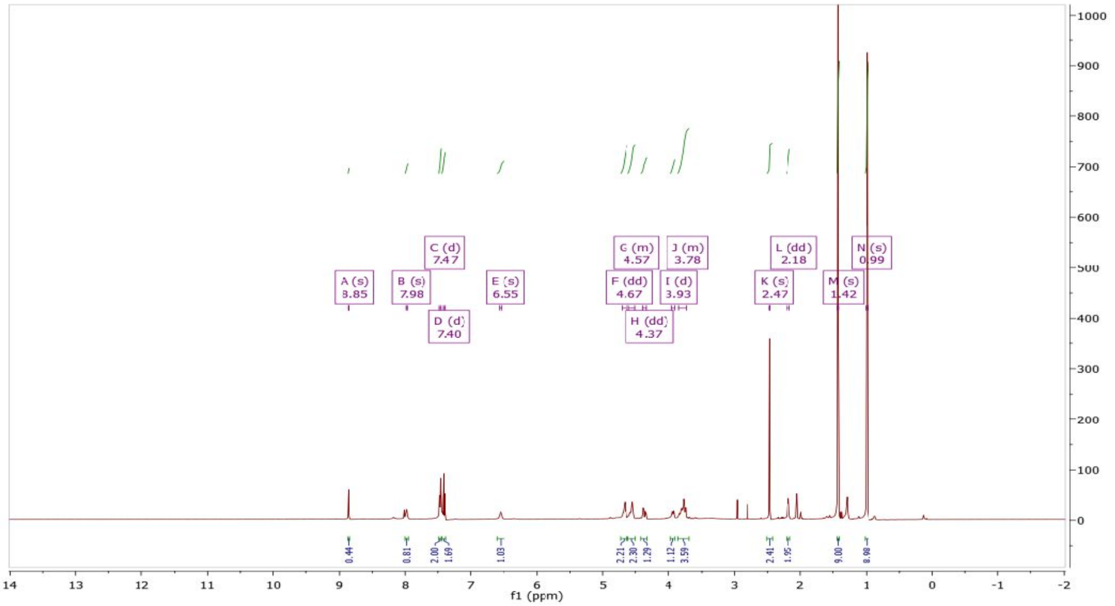
A (s) 8.85
B (s) 7.98
C (d) 7.47
D (d) 7.40
E (s) 6.55
F (dd) 4.67
G (m) 4.57
H (dd) 4.37
I (d) 3.93
J (m) 3.78
K (s) 2.47
L (dd) 2.18
M (s) 1.42
N (s) 0.99
0.44
0.81
2.00
1.69
1.03
2.21
2.30
1.29
1.12
3.59
2.41
1.95
9.00
8.98
f1 (ppm)

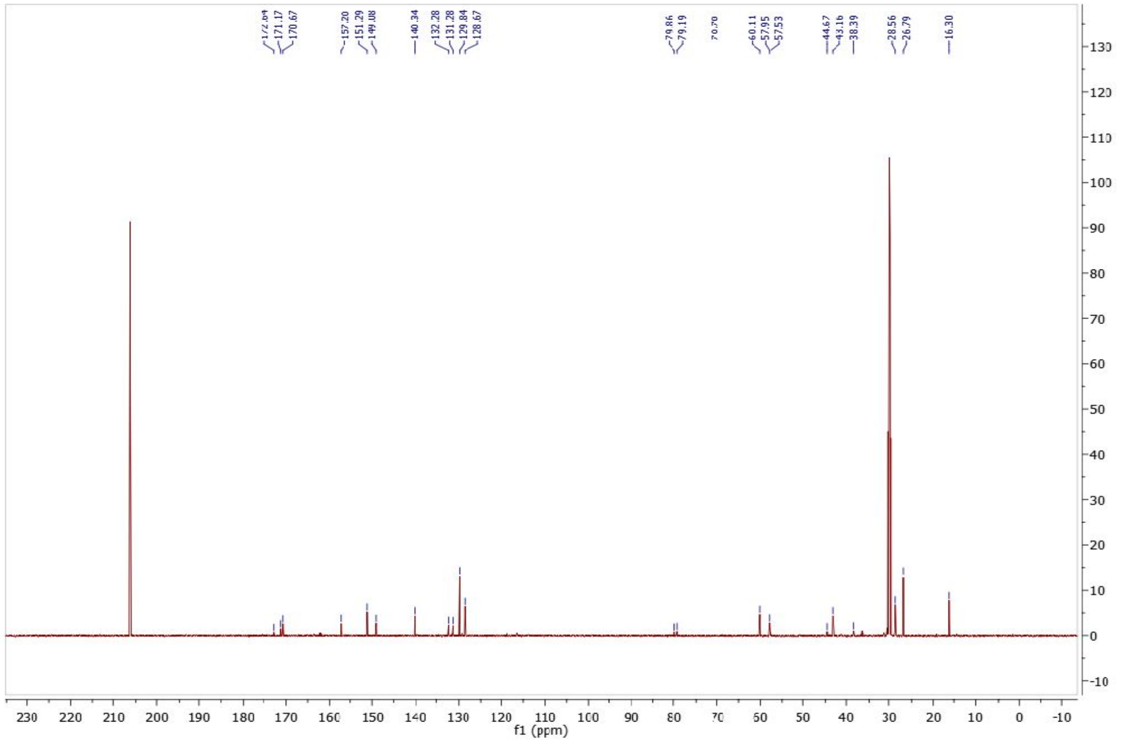
172.64
171.17
170.67
157.20
151.29
149.08
140.34
132.28
131.28
129.84
128.67
79.86
79.19
70.70
60.11
57.95
57.53
44.67
43.16
38.39
28.56
26.79
16.30
f1 (ppm)

Figure S10

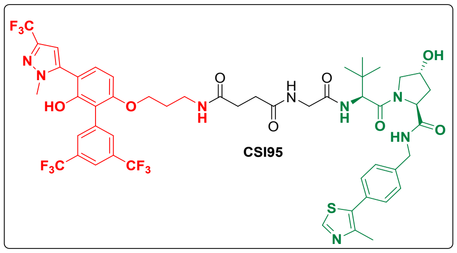

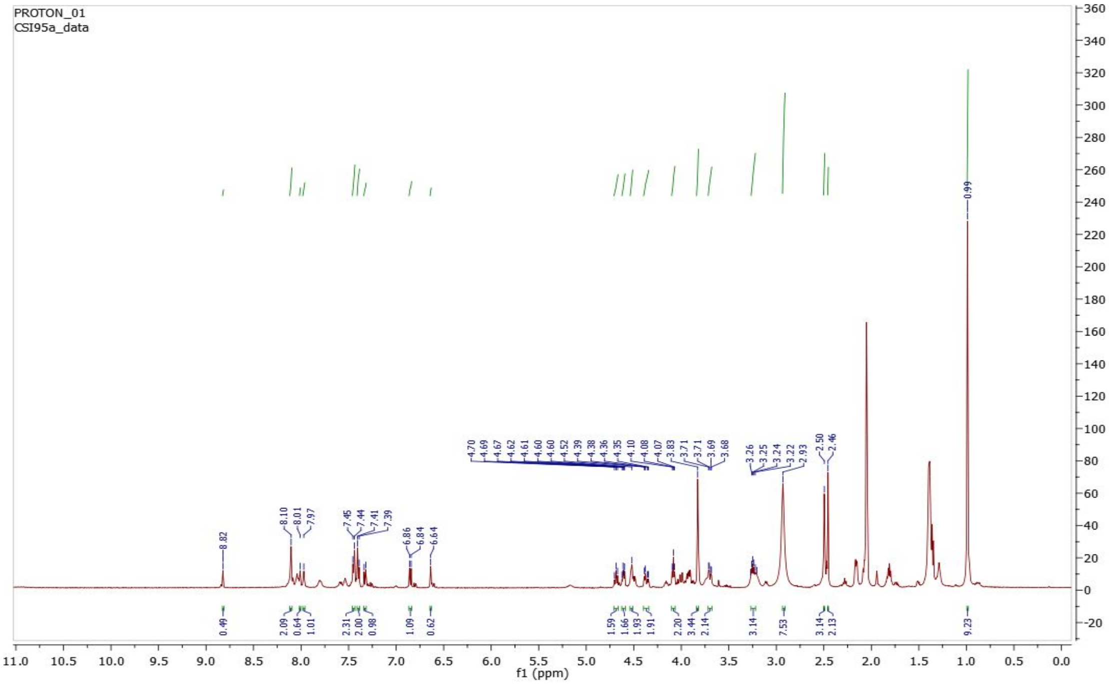

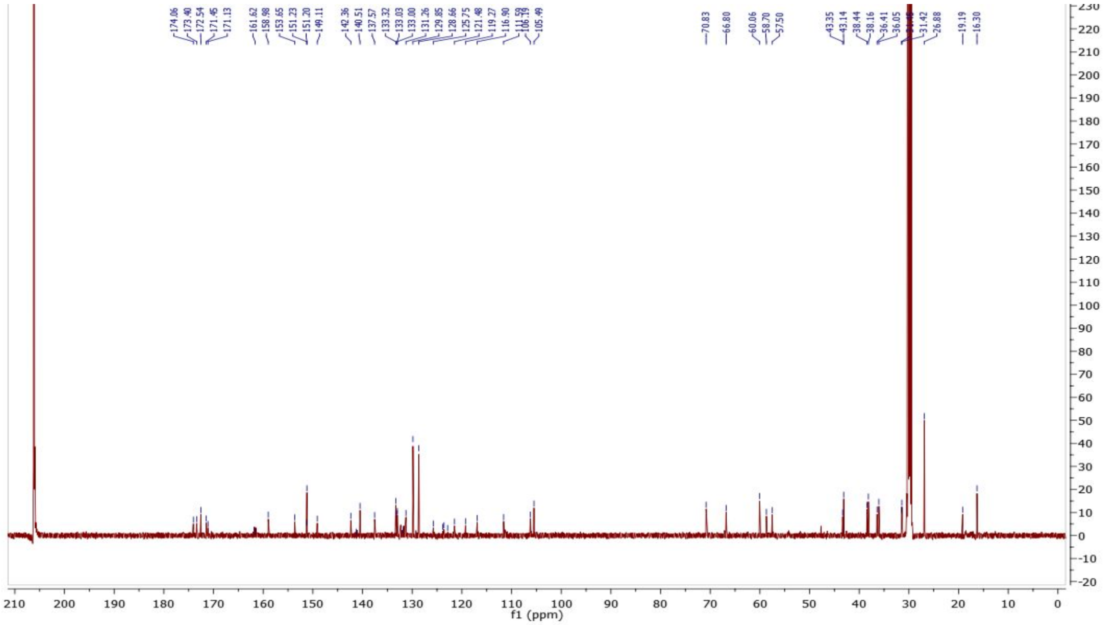

Figure S11

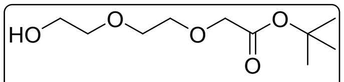

15

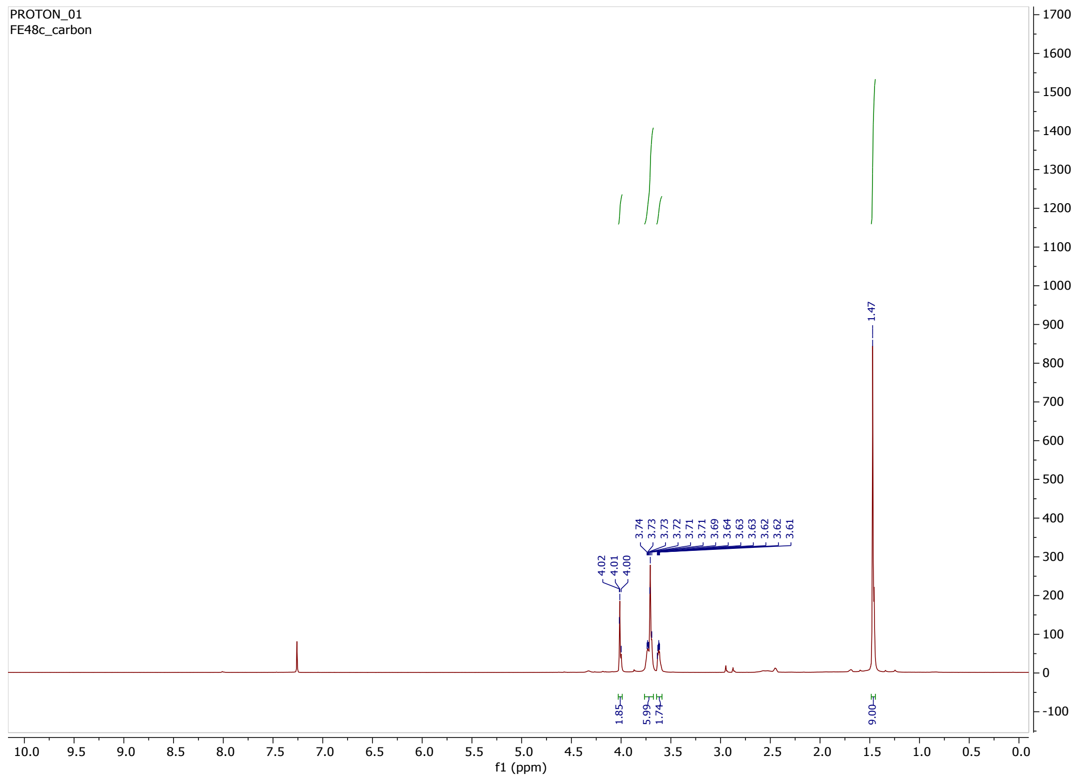

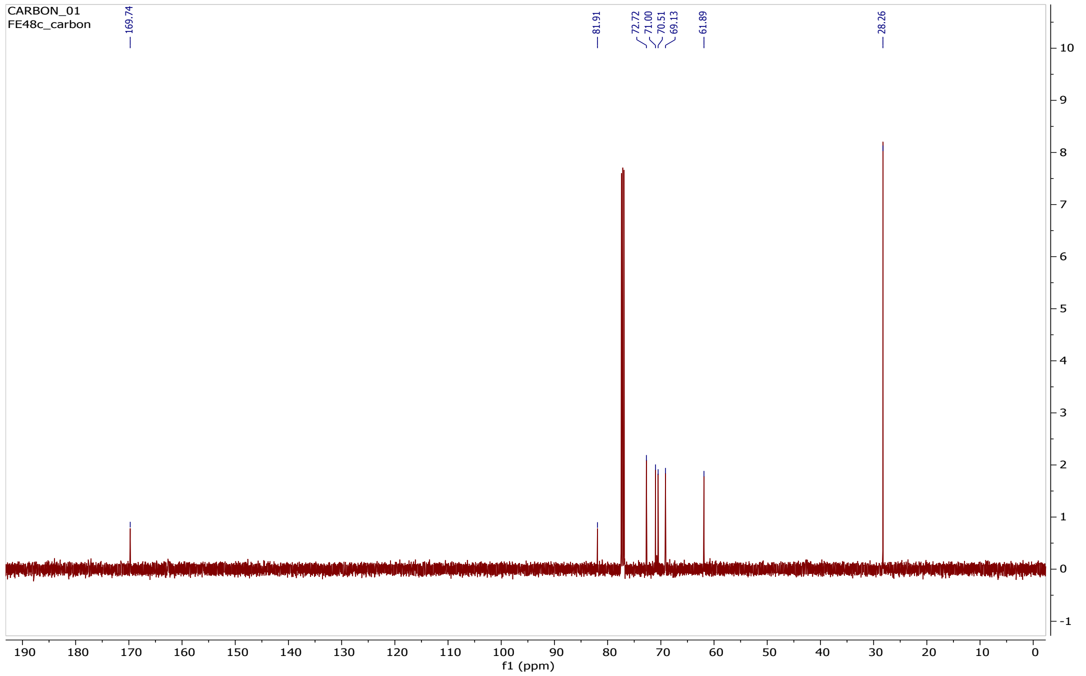

Figure S12

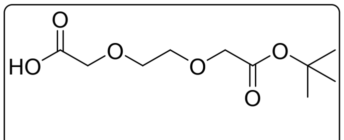

16

EtOAc is observed in the spectra

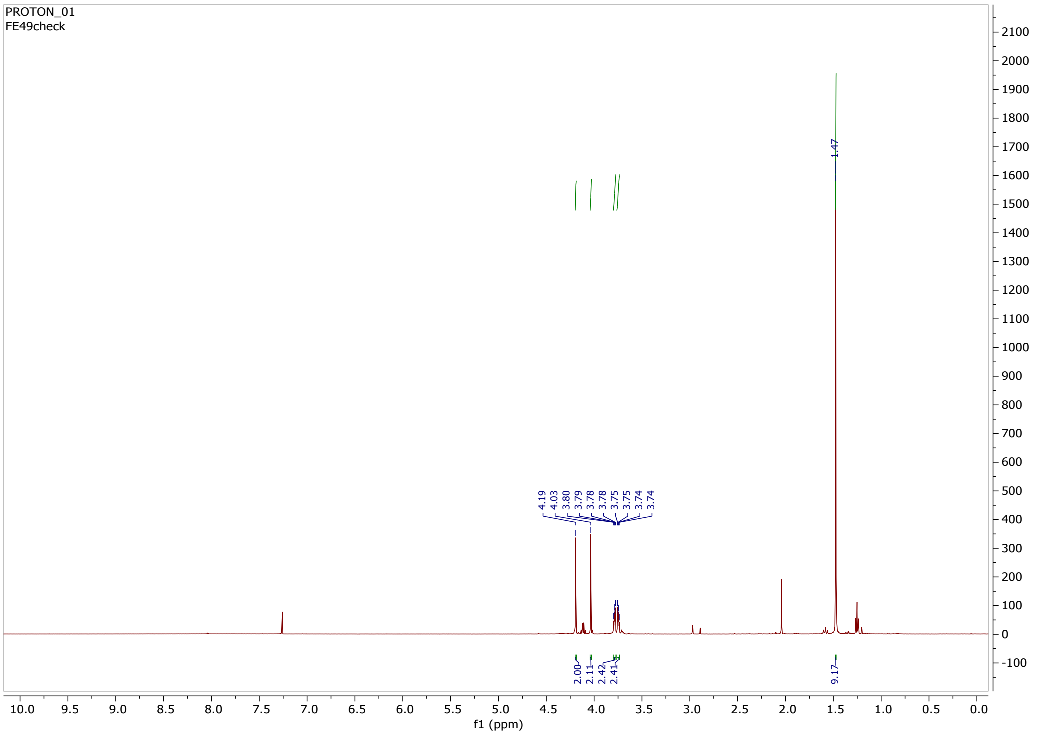

Figure S13

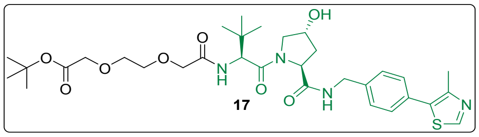

OH
O
N
H
O
N
O
N
H
S
N
O
O
O
O
17

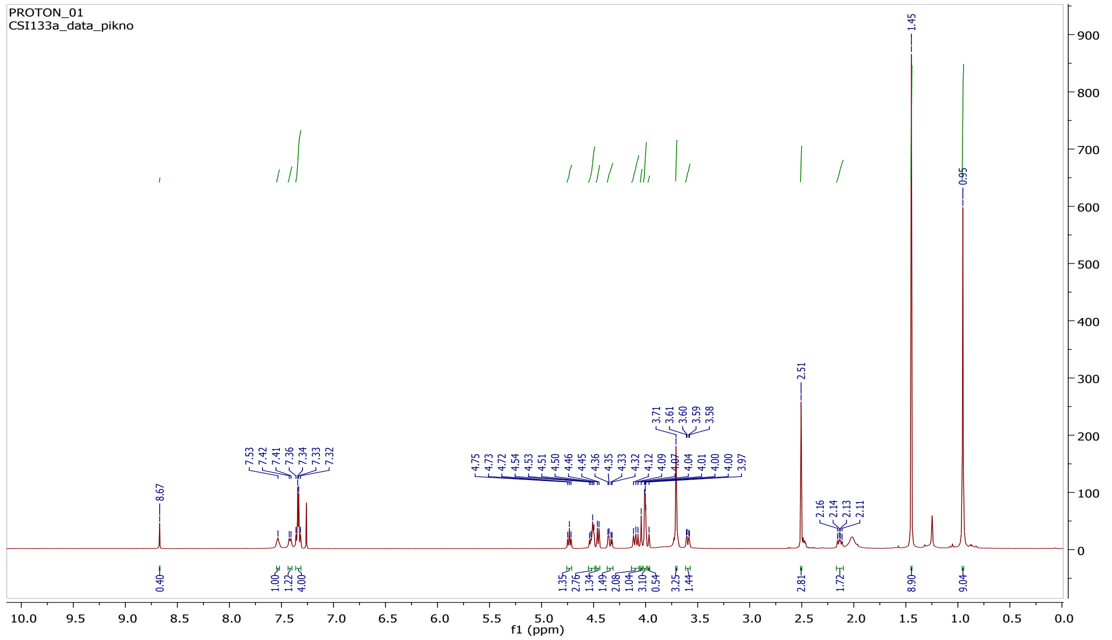

PROTON_01
CSI133a_data_pikno
1.45
0.95
2.51
8.67
7.53 7.42 7.41 7.36 7.34 7.33 7.32
4.75 4.73 4.72 4.54 4.53 4.51 4.50 4.46 4.45 4.36 4.35 4.33 4.32 4.12 4.09 4.07 4.04 4.01 4.00 4.00 3.97
3.71 3.61 3.60 3.59 3.58
2.16 2.14 2.13 2.11
0.40 1.00 1.22 4.00 1.35 2.76 1.34 1.49 2.08 1.04 3.10 0.54 3.25 1.44 2.81 1.72 8.90 9.04
f1 (ppm)

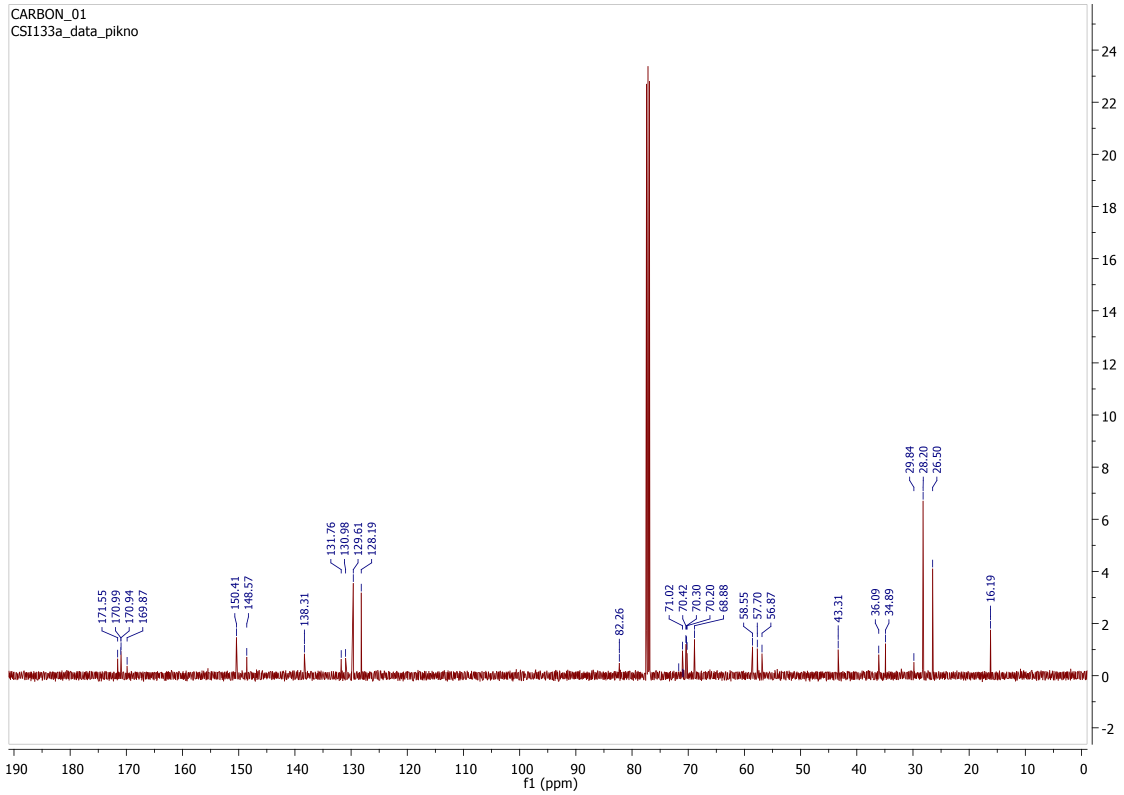

CARBON_01
CSI133a_data_pikno
171.55 170.99 170.94 169.87
150.41 148.57
138.31
131.76 130.98 129.61 128.19
82.26
71.02 70.42 70.30 70.20 68.88
58.55 57.70 56.87
43.31
36.09 34.89
29.84 28.20 26.50
16.19
f1 (ppm)

Figure S14

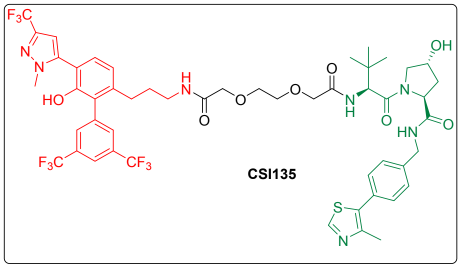
F3C
N
N
HO
F3C
CF3
H
N
O
O
O
O
N
H
O
N
OH
HN
O
S
N
CSI135

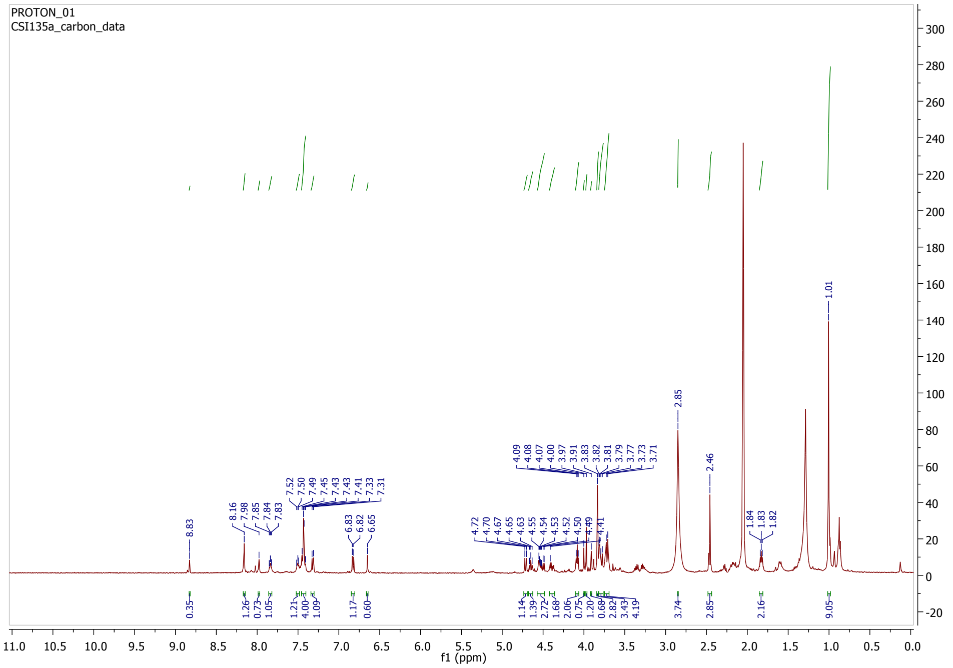
PROTON_01
CSI135a_carbon_data
8.83
8.16 7.98 7.85 7.84 7.83
7.52 7.50 7.49 7.45 7.43 7.43 7.41 7.33 7.31
6.83 6.82 6.65
4.72 4.70 4.67 4.65 4.63 4.55 4.54 4.53 4.52 4.50 4.49 4.41
4.09 4.08 4.07 4.00 3.97 3.91 3.83 3.82 3.81 3.79 3.77 3.73 3.71
2.85
2.46
1.84 1.83 1.82
1.01
0.35 1.26 0.73 1.05 1.21 4.00 1.09 1.17 0.60 1.14 1.39 2.72 1.68 2.06 0.75 1.20 0.68 2.82 3.43 4.19 3.74 2.85 2.16 9.05
f1 (ppm)

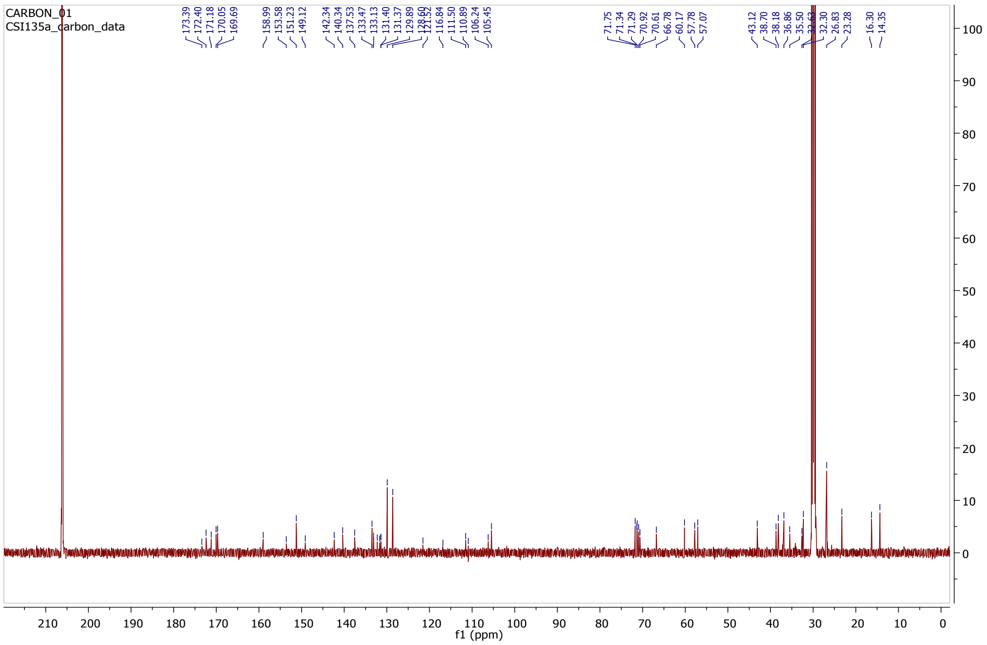
CARBON_01
CSI135a_carbon_data
173.39 172.40 171.18 170.05 169.69
158.99 153.58 151.23 149.12
142.34 140.34 137.53 133.47 133.13 131.40 131.37 129.89 128.60 128.52 121.52 116.84 111.50 110.89 106.24 105.45
71.75 71.34 71.29 70.92 70.61 66.78 60.17 57.78 57.07
43.12 38.70 38.18 36.86 35.50 33.62 32.30 26.83 23.28
16.30 14.35
f1 (ppm)

Figure S15

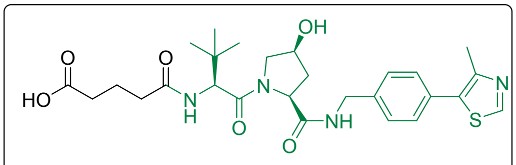

19

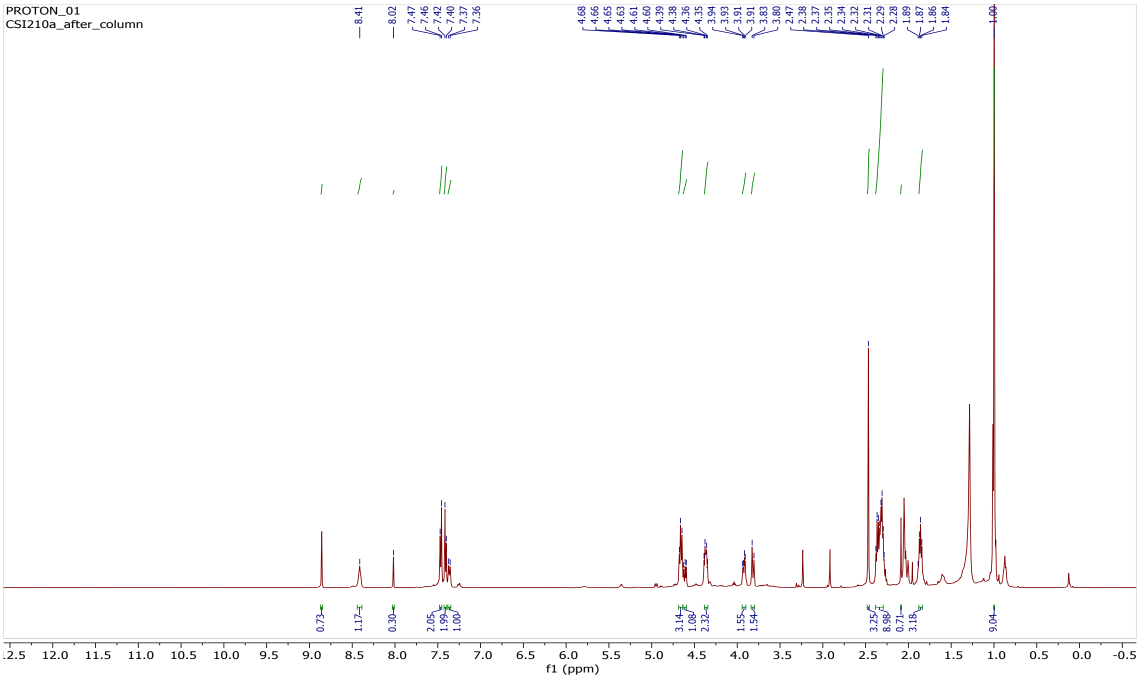

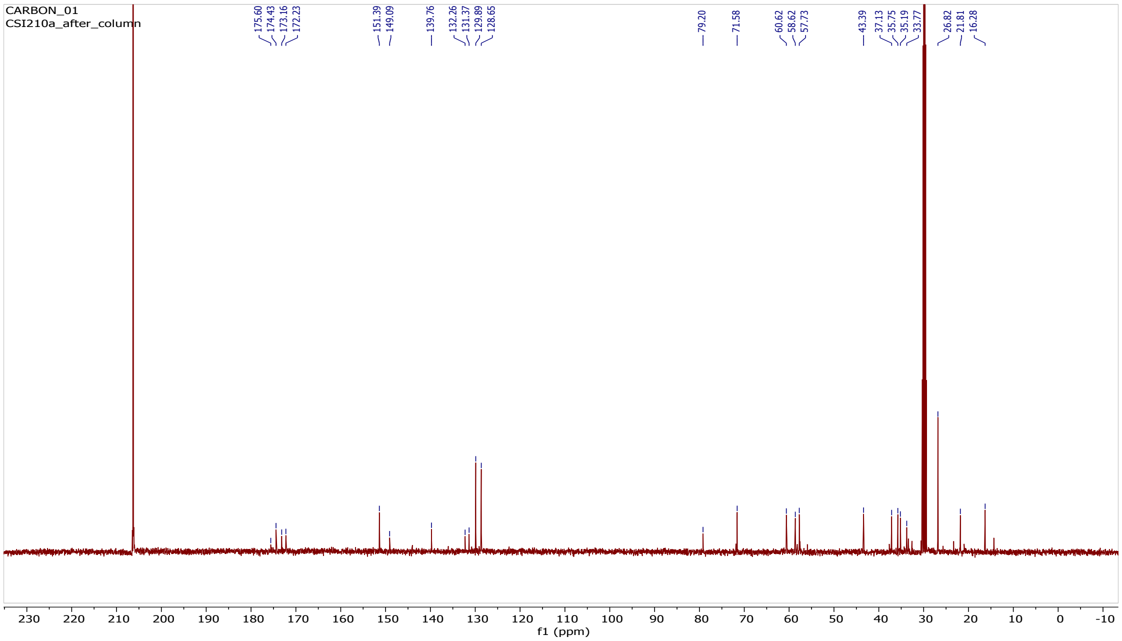

Figure S16

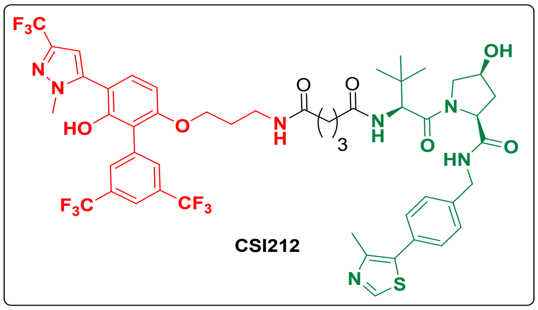

CSI212

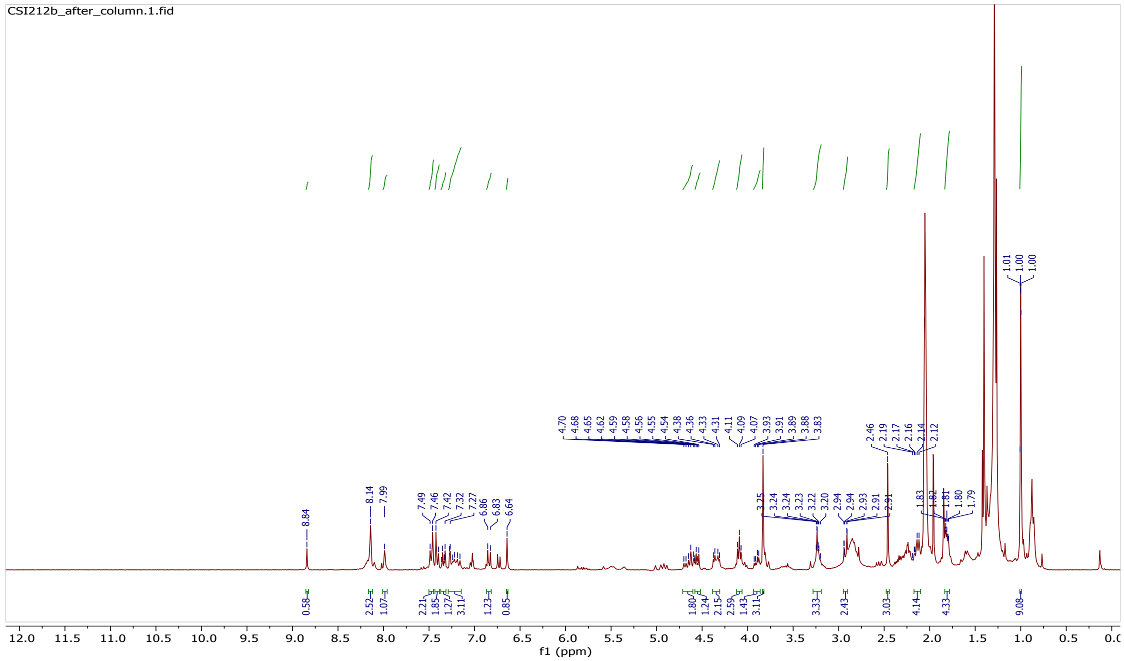

CSI212b_after_column.1.fid
f1 (ppm)

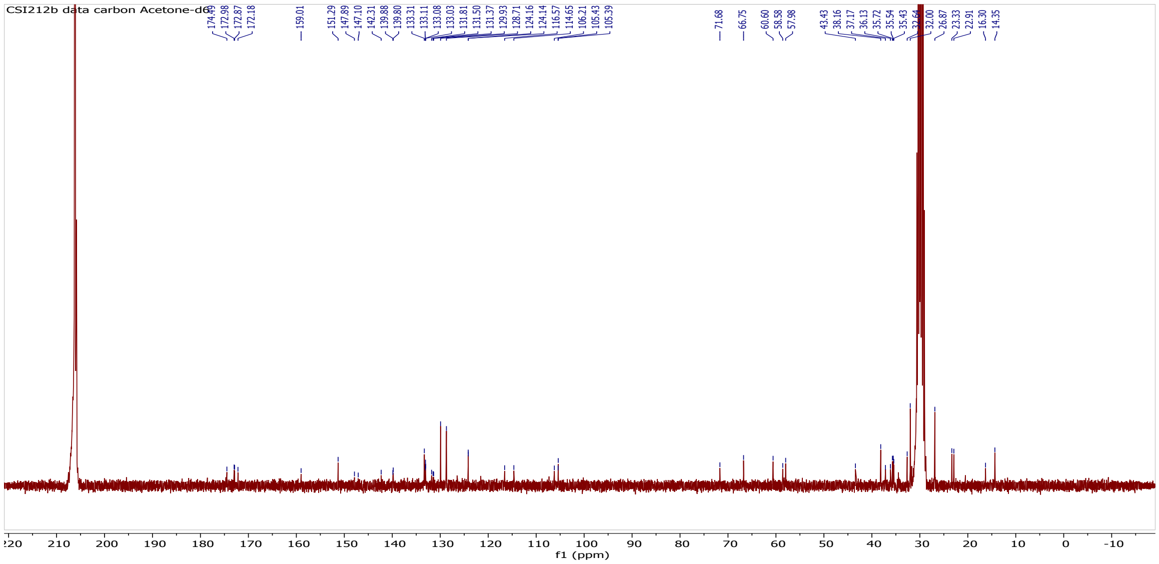

CSI212b data carbon Acetone-d6
f1 (ppm)

Figure S17

Analytical method for the synthesis of Myc degraders and key intermediates

LC/ESI-MS: LC20AD Shimadzu connected to Shimadzu LCMS-2010EV

Mobile Phase A: 0.1% FA in water

Mobile Phase B: 0.1% FA in acetonitrile

Column: SUPELCO Discovery (C18, 25 cm × 4.6 mm, 5 µm)

Flow rate: 0,4 mL/min

Run time: 35 min

Column temperature: 26 °C

UV detector: 254 nm MS detector: 1.65kV

LC/MS method for PROTACs

TIME (MIN)	MOBILE PHASE A (%)	MOBILE PHASE B (%)
**0**	90	10
**3**	90	10
**22**	15	85
**25**	10	90
**29**	10	90
**31**	5	95
**37**	5	95
**39**	90	10

<u>Analytical methods for PROTAC **CSI63**</u>

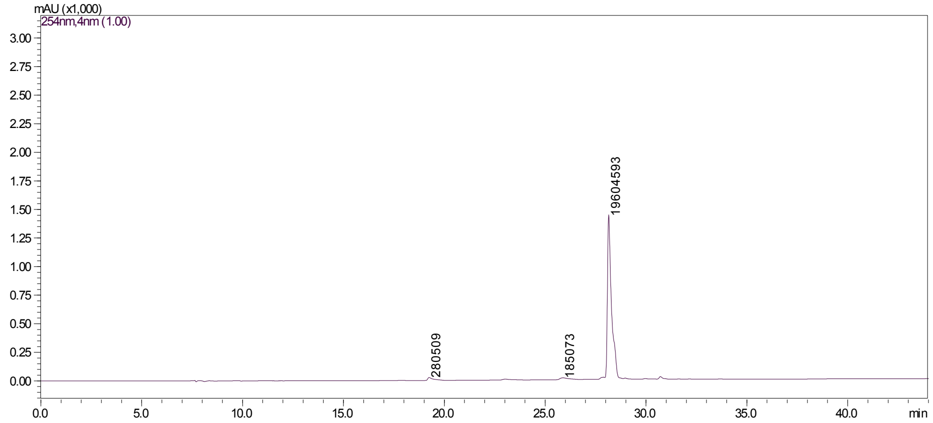

Positive ion mode

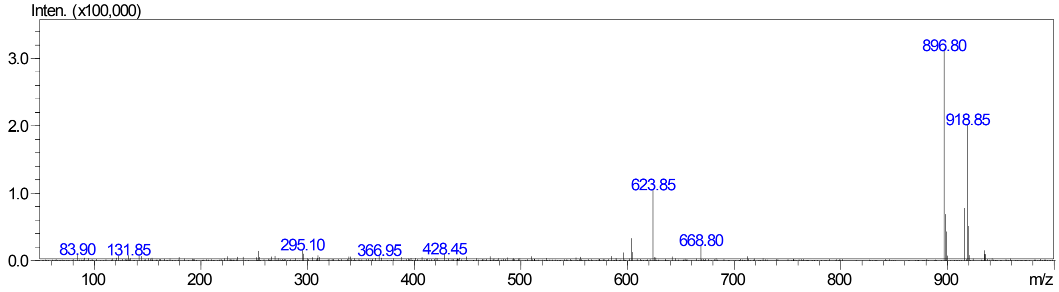

Negative ion mode

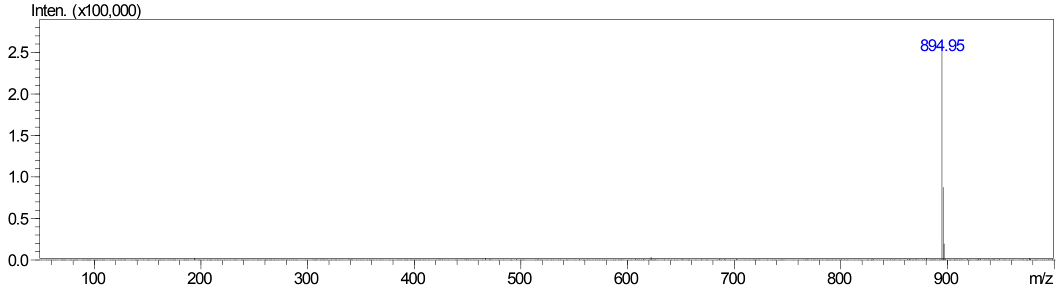

Area: 19604593 mAU*min

Total area: 20070121

Area %: (19604593/20070121)*100%= 97.7%

Figure S18

Analytical methods for PROTAC **CSI90**

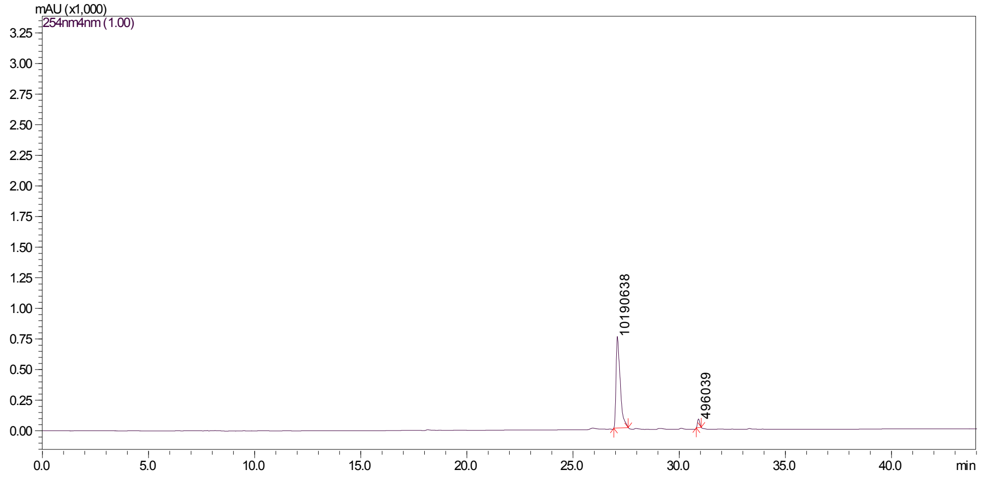

Positive ion mode

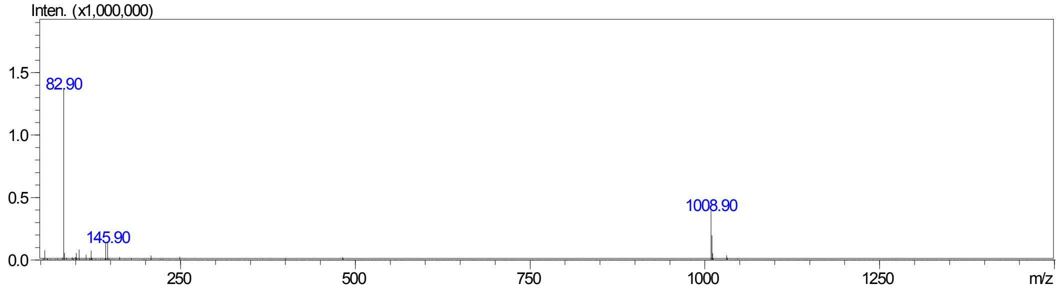

Negative ion mode

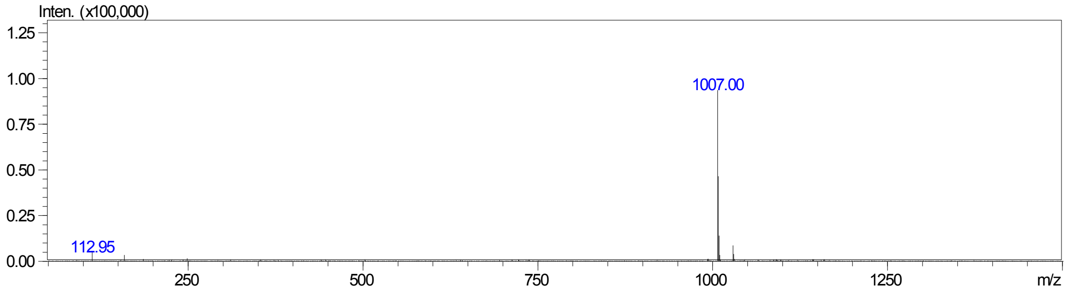

Area: 10190638 mAU*min

Total area: 10686677

Area %: (10190638/10686677)*100%= 95.4%

Figure S19

Analytical methods for PROTAC **CSI86**

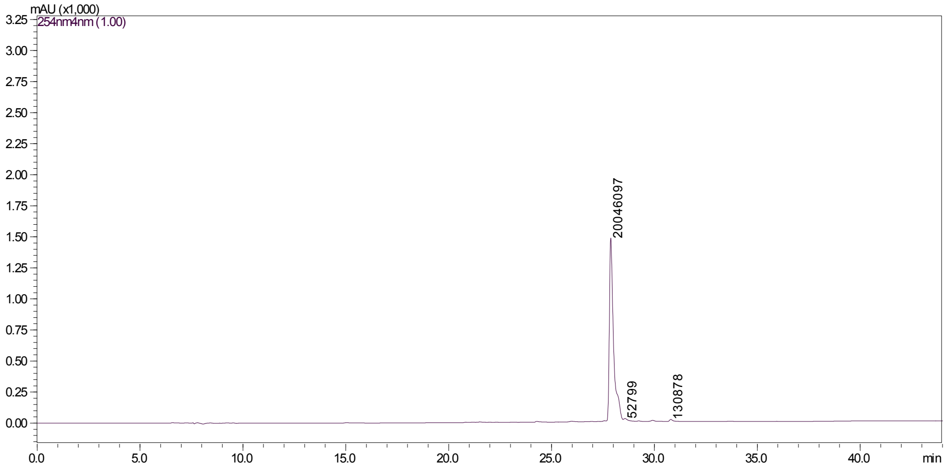

Positive ion mode

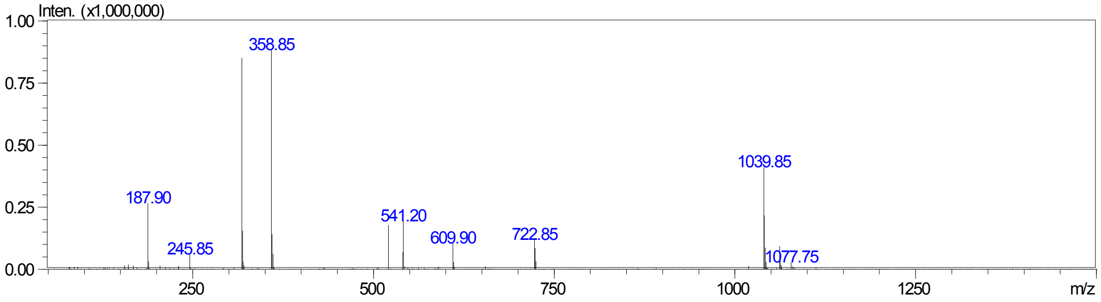

Negative ion mode

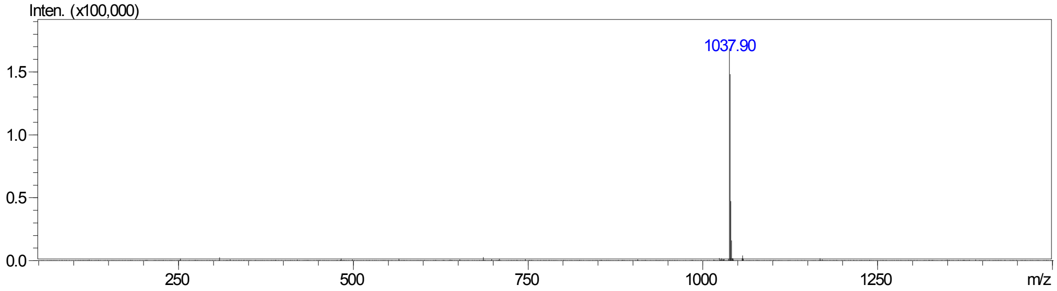

Area: 20046097 mAU*min

Total area: 20229774

Area %: (20046097/20229774)*100%= 99.1%

Figure S20

Analytical methods for PROTAC **CSI95**

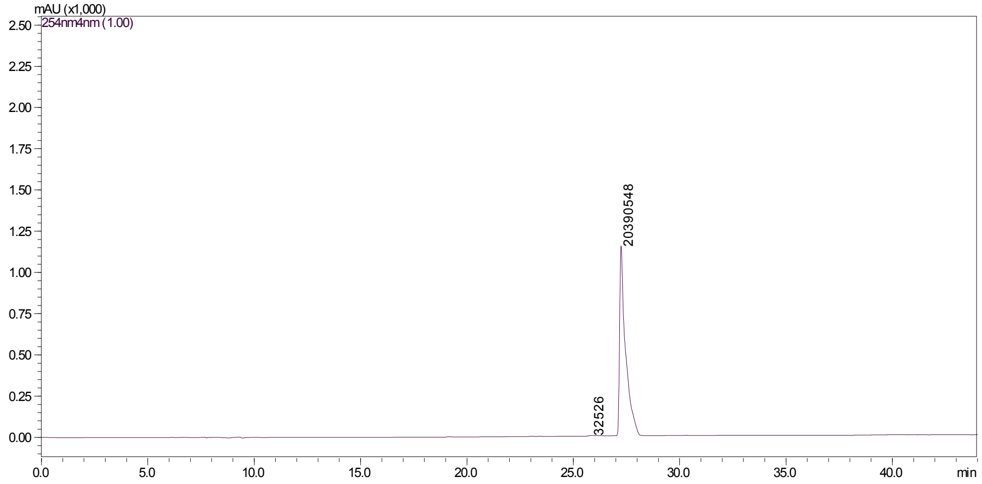

Positive ion mode

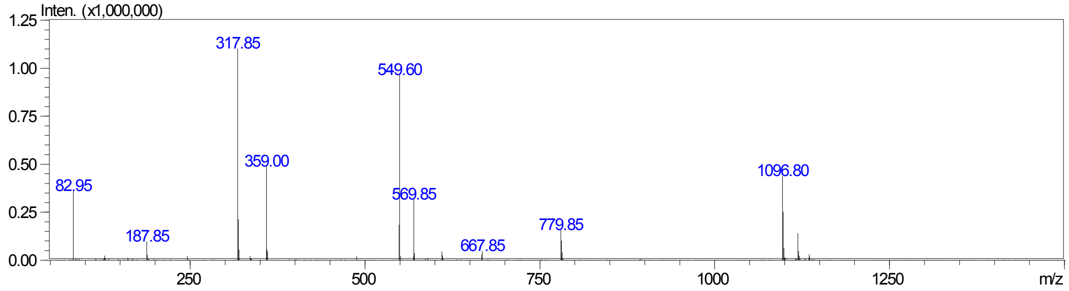

Negative ion mode

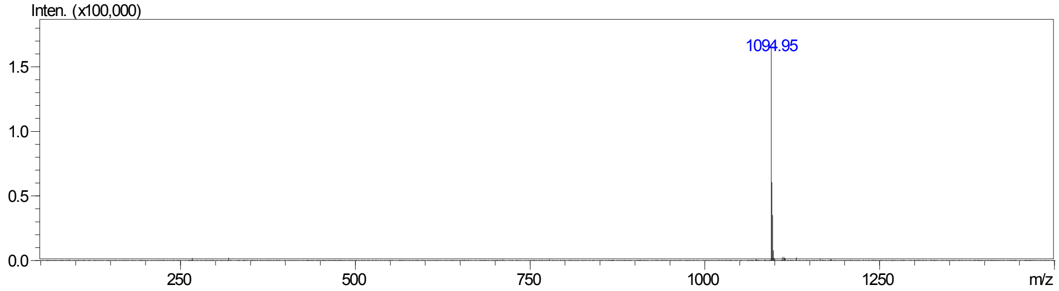

Area: 20390548 mAU*min

Total area: 20423076

Area %: (20390548/20423076)*100%= 99.8%

Figure S21

Analytical methods for PROTAC **CSI107**

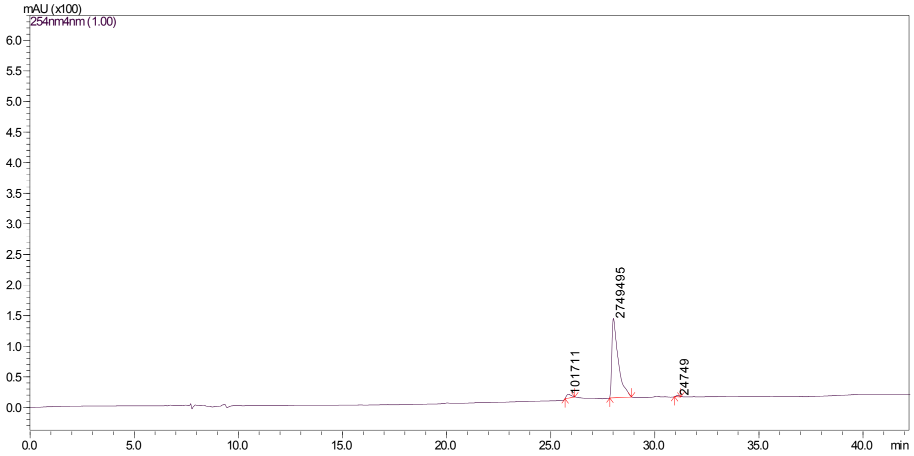

Positive ion mode

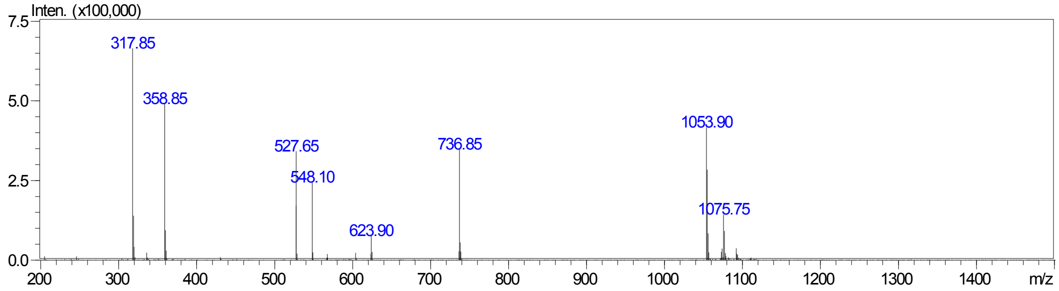

Negative ion mode

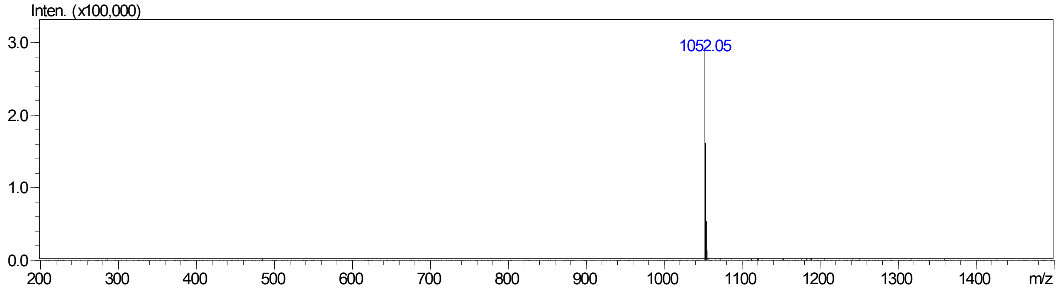

Area: 2749495 mAU*min

Total area: 2875955

Area %: (2749495/2875955)*100%= 95.6%

Figure S22

Analytical method for **4-((3-((6-hydroxy-5-(1-methyl-3-(trifluoromethyl)-1*H*-pyrazol-5-yl)-3',5'-bis(trifluoromethyl)-[1,1'-biphenyl]-2-yl)oxy)propyl)amino)-4-oxobutanoic acid (7)**

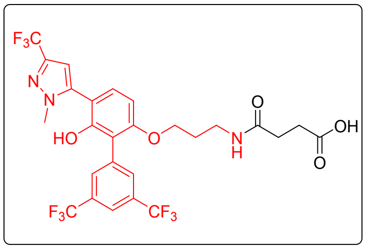

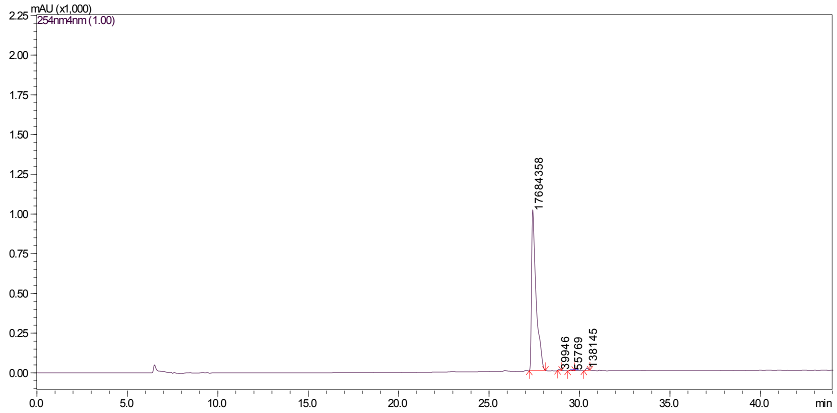

Positive ion mode

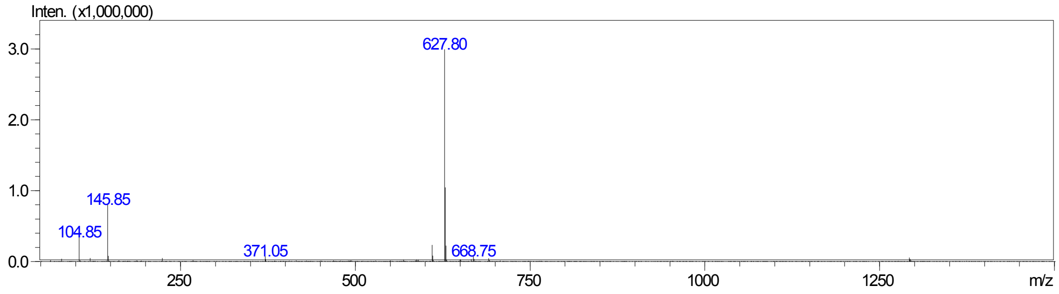

Negative ion mode

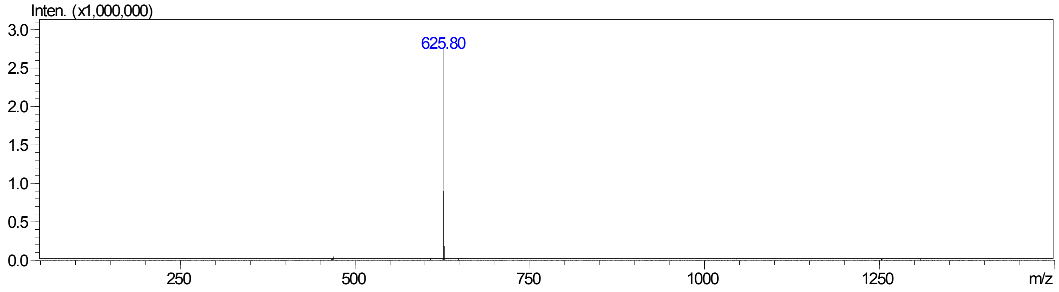

Figure S23

Analytical method for **tert-butyl 2-(2-(2-(((*S*)-1-((2*S*,4*R*)-4-hydroxy-2-((4-(4-methylthiazol-5-yl)benzyl)carbamoyl)pyrrolidin-1-yl)-3,3-dimethyl-1-oxobutan-2-yl)amino)-2-oxoethoxy)ethoxy)acetate (17)**

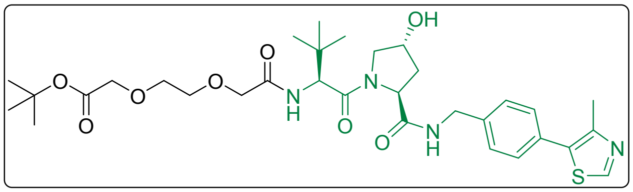

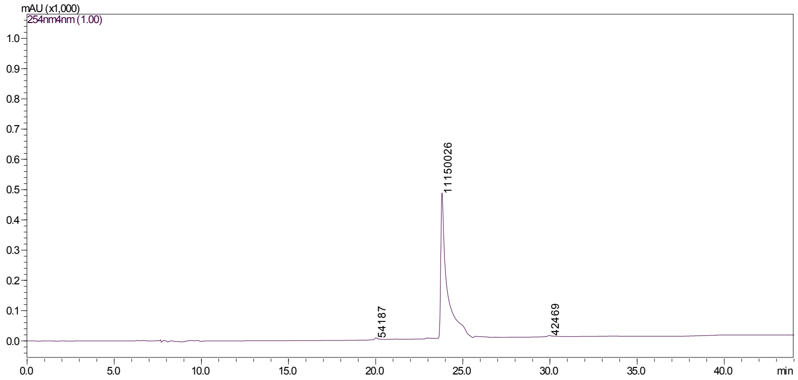

Positive Ion Mode

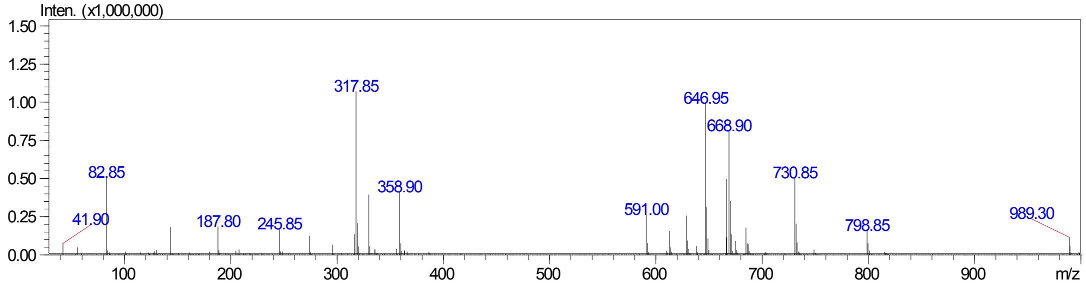

Negative Ion Mode

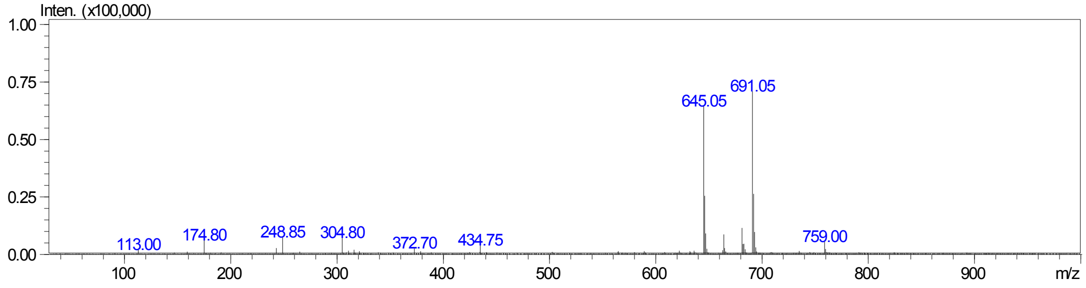

Figure S24

Analytical method for PROTAC **CSI135**

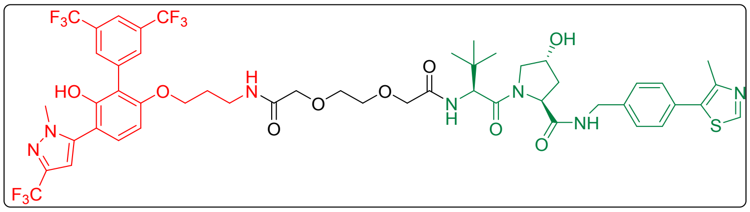

LC-MS for PROTAC CSI135 was taken in 100% MeOH as an eluent

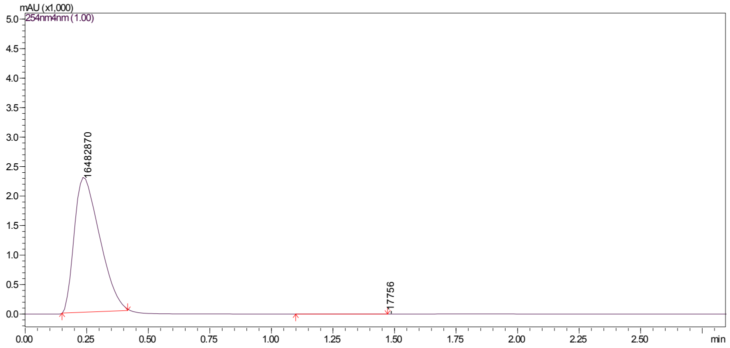

Positive Ion Mode

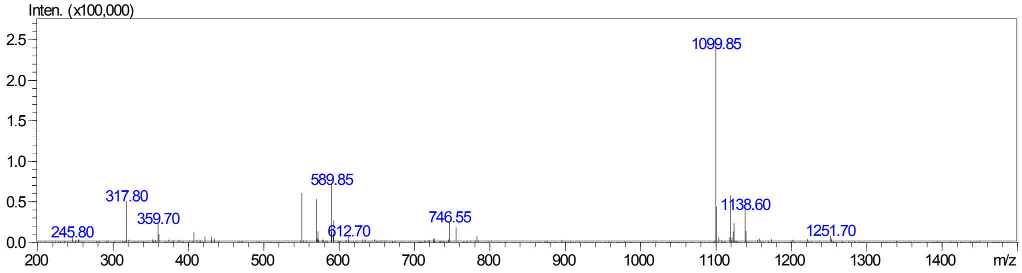

Negative Ion Mode

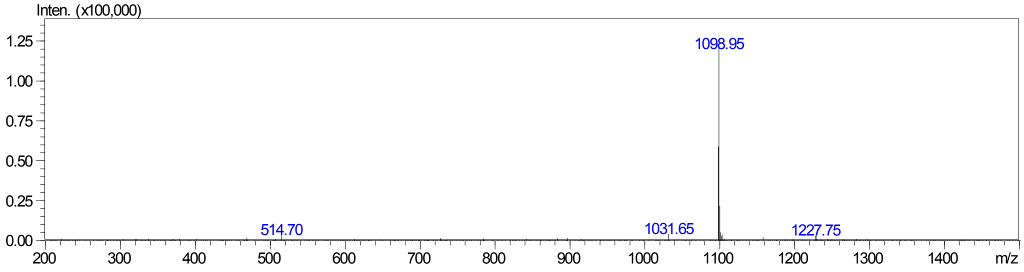

Area: 16482870 mAU*min

Total area: 16500626

Area %: (16482870/16500626)*100% > 98%

Figure S25

Analytical methods for **5-(((*S*)-1-((2*S*,4*S*)-4-hydroxy-2-((4-(4-methylthiazol-5-yl)benzyl) carbamoyl)pyrrolidin-1-yl)-3,3-dimethyl-1-oxobutan-2-yl)amino)-5-oxopentanoic acid** (**19**)

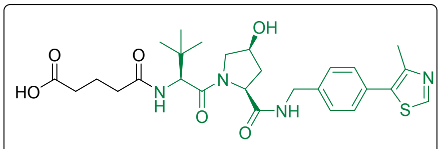

19

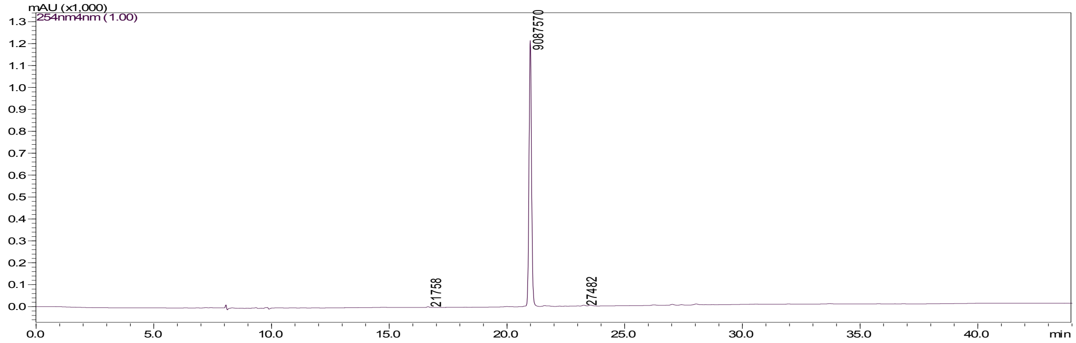

Positive Ion Mode

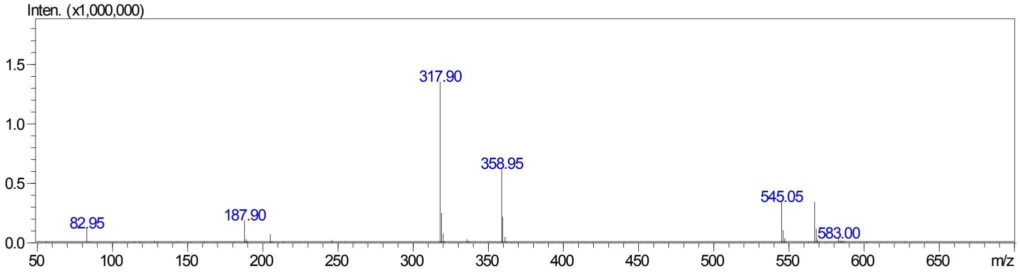

Negative Ion Mode

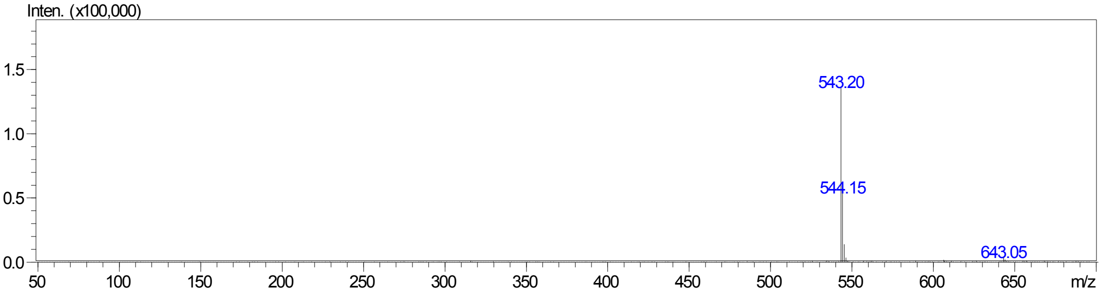

Total Area: 9136810 mAU*min^{-1}

Peak Area: 9087570 mAU*min^{-1}

Purity (%)= (Peak Area/Total Area) *100% = (9087570/9136810)*100% = 99.4%

Figure S26

Analytical methods for **CSI212**

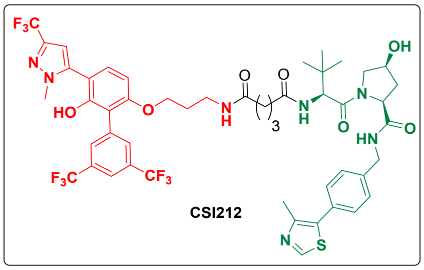

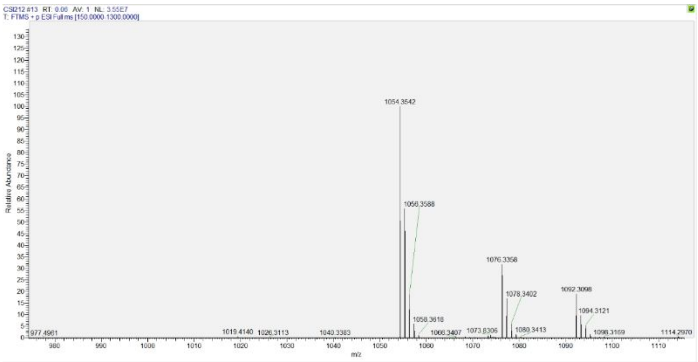

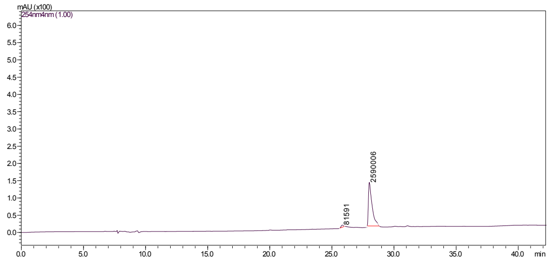

(+)

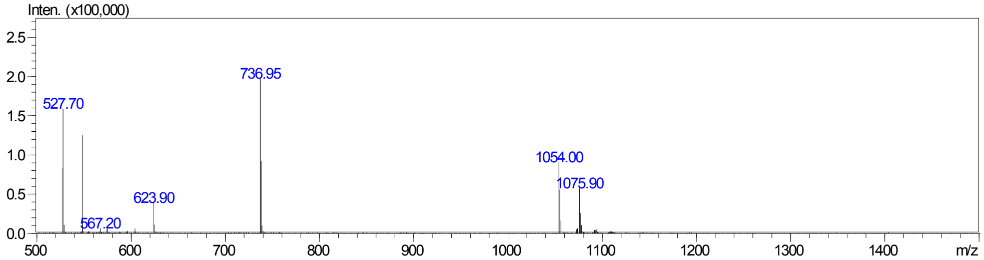

(-)

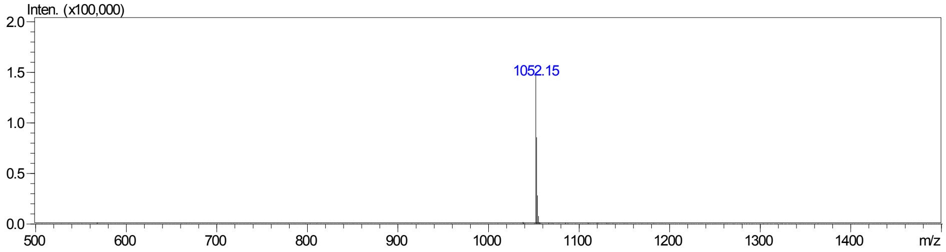

total area 2671597 mAu*min

peak area 2590006

purity (%) = (peak area/total area)*100% = 96.9%

Figure S27

Characterization and Quantitative Analysis of PROTACs by Liquid Chromatography-Mass Spectrometry (LC-MS/MS)

For detection and quantification of the PROTACs in medium and biological samples, LC-MS/MS methodologies were developed. Mass spectrometry was performed on a Triple Quad 5500$^+$ LC-MS/MS system fitted with a TurboIonSpray source and a hybrid triple quadrupole/linear ion trap mass spectrometer. HPLC was performed using an Exion LC AB Sciex system with a temperature-controlled column compartment and an autosampler. A C8 column (SunFire 2.1 x 50 mm, 3.5 µm) (Waters, MA, USA) was used at a flow rate of 0.3 mL/min for the separation of the analytes of interest. The mobile phase consisted of A: 10% ACN, 90% water, 2 mM ammonium acetate, and 0.1% FA and B: 90% ACN, 10% water, 2 mM ammonium acetate, and 0.1% FA, the injection volume was 10 µL. Elution of **CSI86** PROTAC was achieved by a gradient of solvent A – solvent B described as follows: 100% A (0.0 - 0.5 min), 20% A (1.0 – 3.0 min), 100% A (3.1 – 5.1 min). The MS positive ion scan mode revealed the most intense ion at *m/z* 1040.5, for the **CSI86** PROTAC with exact mass of 1039 (g/mol). Elution of **CSI107** PROTAC was achieved by a gradient of solvent A – solvent B described as follows: 80% A (0.0 - 0.5 min), 15% A (1.5 – 3.5 min), 80% A (3.1 – 5.1 min). The MS negative ion scan mode revealed the most intense ion at *m/z* 1052.3, for the **CSI107** PROTAC with an exact mass of 1053 (g/mol).

Table S1: Optimized parameters for the LC-MS/MS analysis.

Compound	Retention time(min)	Q1 MS	MS/MS	DP	CE
**CSI86**	3.08	1040.5	318.3	100	30
		1040.5	610.3	100	26.5
		1040.5	723.3	100	26.0
		520.9	318.1	95.6	26.5
		520.9	723.5	95.6	15.5

Compound	Retention time(min)	Q1 MS	MS/MS	DP	CE
**CSI107**	3.22	1052.3	469.1	-252	-81
		1052.3	425.1	-252	-99
		1052.3	525.9	-252	-83

Detection protein degraders in mouse blood by LC-MS/MS analysis

For the LC-MS/MS analysis of the PK study, sample preparation was performed as follows.

Preparation of samples

Double Blank	Blank + IS	Standards
50 µL blood	50 µL blood	50 µL blood
10 µL ACN: H_2O	10 µL IS	10 µL IS
10 µL ACN: H_2O	10 µL ACN: H_2O	10 µL std

300 µL of cold ACN were added in all samples for protein precipitation, followed by centrifugation at 13000 rpm for 10 min. The supernatant was transferred in glass tubes and evaporated at 55 ^{o}C for approximately 1 h. The samples were reconstituted in Mobile Phase A (90% H_2O-10% ACN, 0.1% FA, 2 mM ammonium acetate) and the circulating amount of PROTAC was determined by LC-MS/MS analysis using a stable internal standard such as Warfarin or BETd-260 respectively. Gradient (20% A – 80% B) LC-MS/MS conditions were applied and the concentration range for the standard curve was 1 - 10.000 ng/mL.

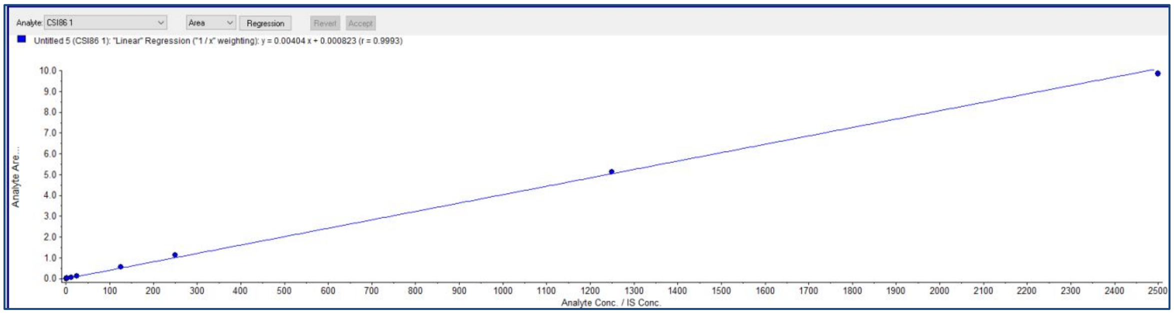

Figure S28

MYC Degradation following treatment with PROTACs in PC3 Cell Lines

CSI107 at 10 µM effectively mediated c-MYC degradation after 3h incubation in PC3 cells. This suggests that CSI107- mediated c-MYC degradation occurred in a concentration (**Figure 3B** of the main manuscript) - and time - dependent manner (** $p < 0.01$).

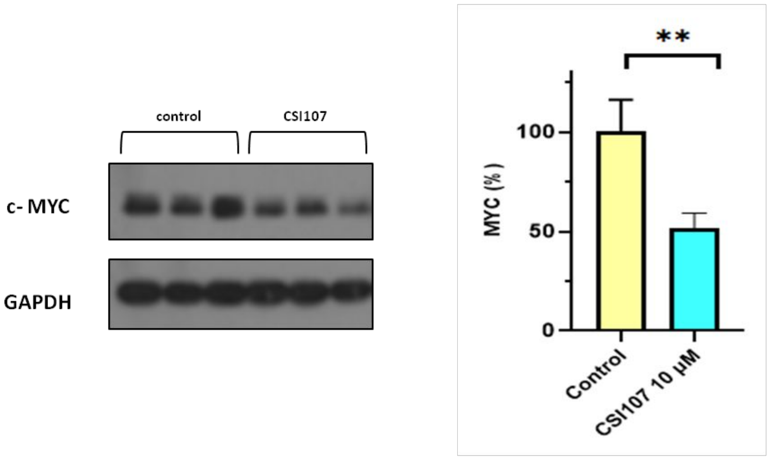

Figure S29

Reference

[1] Han, H.; Jain, AD.; Truica, M.I.; Izquierdo-Ferrer, J.; Anker, J.F.; Lysy, B.; Sagar, V.; Luan, Y.; Chalmers, Z.R.; Unno, K.; Mok, H.; Vatapalli, R.; Yoo, Y.A.; Rodriguez, Y.; Kandela, I.; Parker, J.B.; Chakravarti, D.; Mishra, R.K.; Schiltz, G.E.; Abdulkadir, S.A. Small-Molecule MYC Inhibitors Suppress Tumor Growth and Enhance Immunotherapy. *Cancer Cell.* **2019**, *36*(5), 483-497.e15.

[2] Konstantinidou, M.; Oun, A.; Pathak, P.; Zhang, B.; Wang, Z.; Ter Brake, F.; Dolga, A.M.; Kortholt, A.; Dömling, A. The tale of proteolysis targeting chimeras (PROTACs) for Leucine-Rich Repeat Kinase 2 (LRRK2). *ChemMedChem*. **2021**, *16*(6), 959-965.

[3] Yao, Z.; Luo, C.; Xie, Y.; Yue, L.; Wan, W.; Zhang, Y.; Jiang, H.; Chen, K. A class of isoindolone-imide ring-1,3-dione-2-ene compounds, composition and use thereof, Wigen Biomedicine Technology Shanghai - US2020/71291, 2020, A1.

[4] Crew, A.P.; Raina, K.; Dong, H.; Qian, Y.; Wang, J.; Vigil, D; Serebrenik, Y.V.; Hamman, B.D.; Morgan, A.; Ferraro, C.; Siu, K.; Neklesa, T.K.; Winkler, J.D.; Coleman, K.G.; Crews, C.M. Identification and Characterization of Von Hippel-Lindau-Recruiting Proteolysis Targeting Chimeras (PROTACs) of TANK-Binding Kinase 1. *J Med Chem.* **2018**, *61*(2), 583-598.
